# Cyclic ether and anhydride ring opening copolymerisation delivering new ABB sequences in poly(ester-*alt*-ethers)†

**DOI:** 10.1039/d4sc02051k

**Published:** 2024-06-27

**Authors:** Ryan W. F. Kerr, Alexander R. Craze, Charlotte K. Williams

**Affiliations:** a Department of Chemistry, Chemistry Research Laboratory 12 Mansfield Road Oxford OX1 3TA UK charlotte.williams@chem.ox.ac.uk

## Abstract

Poly(ester-*alt*-ethers) are interesting as they combine the ester linkage rigidity and potential for hydrolysis with ether linkage flexibility. This work describes a generally applicable route to their synthesis applying commercial monomers and yielding poly(ester-*alt*-ethers) with variable compositions and structures. The ring-opening copolymerisation of anhydrides (A), epoxides (B) and cyclic ethers (C), using a Zr(iv) catalyst, produces either ABB or ABC type poly(ester-*alt*-ethers). The catalysis is effective using a range of commercial anhydrides (A), including those featuring aromatic, unsaturated or tricyclic monomers, and with different alkylene oxides (epoxides, B), including those featuring aliphatic, alkene or ether substituents. The range of effective cyclic ethers (C) includes tetrahydrofuran, 2,5-dihydrofuran (DHF) or 1,4-bicyclic ether (OBH). In these investigations, the catalyst:anhydride loadings are generally held constant and deliver copolymers with degrees of copolymerisation of 25, with molar mass values from 4 to 11 kg mol^−1^ and mostly with narrow dispersity molar mass distributions. All the new copolymers are amorphous, they show the onset of thermal decomposition between 270 and 344 °C and variable glass transition temperatures (−50 to 48 °C), depending on their compositions. Several of the new poly(ester-*alt*-ethers) feature alkene substituents which are reacted with mercaptoethanol, by thiol–ene processes, to install hydroxyl substituents along the copolymer chain. This strategy affords poly(ether-*alt*-esters) featuring 30, 70 and 100% hydroxyl substituents (defined as % of monomer repeat units featuring a hydroxyl group) which moderate physical properties such as hydrophilicity, as quantified by water contact angles. Overall, the new sequence selective copolymerisation catalysis is shown to be generally applicable to a range of anhydrides, epoxides and cyclic ethers to produce new families of poly(ester-*alt*-ethers). In future these copolymers should be explored for applications in liquid formulations, electrolytes, surfactants, plasticizers and as components in adhesives, coatings, elastomers and foams.

## Abstract 2

The synthesis of poly(ester-*alt*-ethers) is achieved by controlled ring-opening copolymerizations of commercial anhydrides and epoxides using a Zr(iv) catalyst. The catalysis shows unusual ABB monomer sequence selectivity and polymer properties depend upon both the monomers and the sequence.

## Graphical abstract

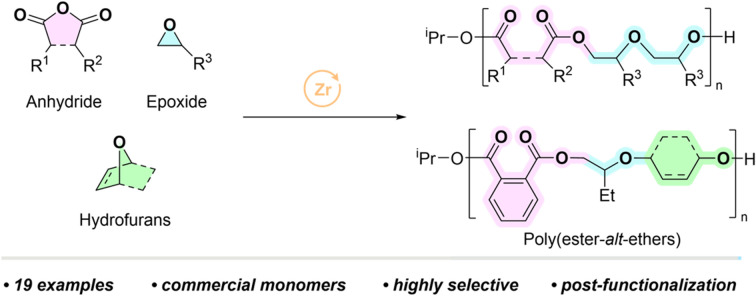

## Introduction

Oligomeric and low molar mass oxygenated copolymers, including polyethers and esters, are useful in formulations for cleaning, beauty, personal care, food, paints, agricultural coatings, energy storage and medicine.^1–7^ They are also widely used to make higher polymers including gels, resins, polyurethane foams, coatings, elastomers, sealants and adhesives.^8–10^ Since copolymers used in formulations tend to become environmentally dispersed after use or are highly diluted in mixed wastes, they should be designed to be degradable. Many current formulations and polyols comprise polyethers, but these structures are usually not degradable.^2,11–13^ In contrast, polyesters used in formulations are degradable by hydrolysis and, in some cases, are also biodegradable but they are generally hydrophobic. Poly(ester-*alt*-ethers) may be able to combine the beneficial properties of each linkage, *i.e.* the degradability of esters with the flexibility and potential to moderate hydrophilicity or functionality of ethers.^14^ However, these interesting alternating copolymers remain under-explored, perhaps due to challenges in accessing appropriate monomers or difficulties in controlling sequences.

One interesting synthesis involves the controlled ring-opening copolymerisation (ROP) of lactones 1,4-dioxane-2-one (PDX) or 3-methyl-1,4-dioxane-2-one (MDO) to produce poly(ester-*alt*-ether)s.^15–18^ However, these cyclic esters are not synthesised very easily at scale and due to the low ceiling temperatures may require careful consideration of the ROP thermodynamics and may show compromised high temperature processing (*T*_c_ = 235 °C for PPDX).^15^ Furthermore, accessing a wide range of other poly(ester-*alt*-ethers) through ROP would require specialist lactone syntheses in each case. Recently, poly(ester-*ran*-ether)s were formed from a two-step melt polycondensation of 2,5-furandicarboxylic acid and ethylene glycol, using a Sc(OTf)_3_ catalyst.^19^ Future studies to access poly(ester–ethers) should prioritise use of inexpensive, readily available starting materials and access a range of structures with control over molar mass and chain length.

In 2022, we reported a new route to poly(ester-*alt*-ethers) exploiting the catalytic copolymerisation of an anhydride and an epoxide.^20^ These new copolymers feature anhydride–epoxide–epoxide or ABB sequences. The copolymerisation required a specific Zr catalyst (1) which exerted its unusual selectivity ensuring that each anhydride insertion is followed by two epoxide insertions. Usually, the ring-opening copolymerisation (ROCOP) of anhydrides (A) and epoxides (B) produces perfectly alternating polyesters, *i.e.*, with AB sequences.^21–24^ There is a need to investigate further the generality of this synthetic approach to poly(ester-*alt*-ethers) and specifically to understand the capability to use the Zr(iv) catalyst with other types of anhydrides and epoxides. In the literature the only related reports concern epoxide and anhydride copolymerisations which produce mixed polymer sequences, including partial ABB-repeats.^25,26^ For example, Phomphrai and co-workers applied Sn(ii) catalysts in epoxide/anhydride ROCOP to produce poly(ester-*ran*-ethers) containing up to 70% ABB sequence selectivity.^26^ Our team also demonstrated that a Sn(octoate)_2_/alcohol catalyst system furnished poly(ester-*ran*-ethers), featuring epoxide : anhydride ratios of ∼3 : 1, and these were used to form block copolymers with l-lactide.^27^ The copolymers were effective as rubber toughening agents for commercial bio-derived plastic, poly(l-lactide), PLLA.

**Scheme 1:**
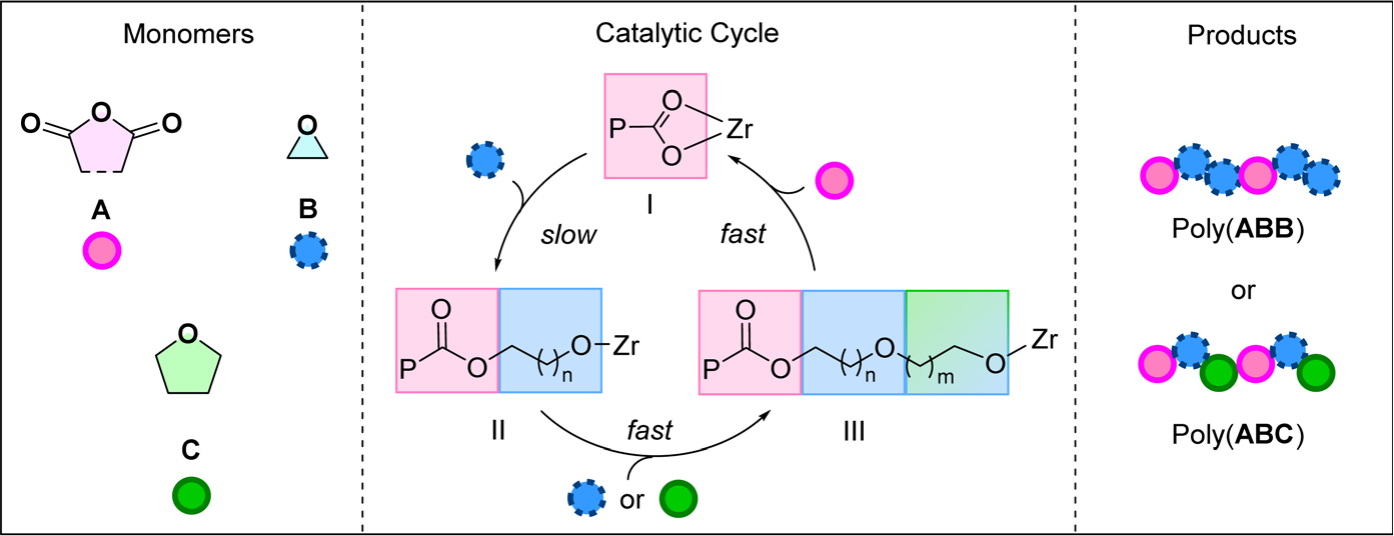
Left: Monomers used within this work, Middle: catalytic cycles for anhydride/cyclic ether ring-opening copolymerisation (ROCOP), Right: ABB or ABC poly(ester-*alt*-ethers) formed by the Zr(iv) catalyst, P: polymeryl chain; Zr: metal active site. All monomers/polymers represented by simplest substitution pattern.

Previously, catalyst (1) was investigated using phthalic anhydride, PA (A), and butylene oxide, BO (B) in the ROCOP.^20^ It yielded poly(ester-*alt*-ethers) with ABB sequences in >95% selectivity (by ^1^H NMR spectroscopy). Further, ROCOP using PA (A), BO (B) and THF (C), catalysed by 1, afforded copolymers with mixtures of –ABB– and –ABC– sequences (>95% by ^1^H NMR spectroscopy). The new poly(ester-*alt*-ethers) showed faster degradation than analogous perfectly alternating polyesters with –AB– sequences in an alkaline aqueous environment.^20^ Analysis of the copolymerisation kinetics revealed a rate-law that was first order in both catalyst (1) and epoxide (B) concentrations and zero order in anhydride (A) concentration and, where THF (C) was present it was also zero order. The kinetic data were rationalised by a proposed catalytic cycle and mechanism (Scheme 1). The polymerisation is initiated by the reaction between the Zr-alkoxide complex and the anhydride to form a Zr-carboxylate species (I). The rate limiting step in the catalysis is proposed as the Zr-carboxylate intermediate, I, attacking the epoxide. This reaction generates a Zr-alkoxide intermediate II, which reacts rapidly with either another epoxide (B) or a cyclic ether (C) to form a second, chain lengthened, Zr-alkoxide, III. Species III rapidly inserts the anhydride to (re)generate the Zr-carboxylate intermediate, I, which is the catalyst resting state. Control experiments revealed no reaction between the anhydride, A, and cyclic ether, C. This finding indicates the cyclic ether is only enchained in the second cyclic ether insertion step. Also, by systematically changing the concentration of epoxide:THF, B:C, and understanding the composition of the resulting copolymers, it was proposed that THF reacts around 2× faster than BO in the second B/C insertion step.

In addition to delivering useful properties for polymers, it's important to address sustainability concerns throughout the polymer life cycle. Current trajectories predict that CO_2_ emissions from the plastic industries could exceed 6.5 Gt per annum by 2050, threatening global net-zero targets.^28^ One option to help manage greenhouse gas emissions is the use of bio-based monomers.^29^ Anhydrides and epoxides can be made from biomass and those most readily produced should prioritised as monomers (see Section S3 and Schemes S1–S14†). This work focusses on understanding the generality of the novel catalysis using a range of different epoxides, anhydrides and cyclic ethers, which have the potential to be sourced from biomass. The objective is to understand whether the copolymerisation operates equivalently with other monomers and, if appropriate, to exploit the sequence selectivity to deliver low molar mass, functionalised ABB/ABC poly(ester-*alt*-ethers) for future use in formulations.

## Results and discussion

First the monomer scope was systematically investigated using a series of 6 anhydrides and 8 epoxides (Table 1). Monomer selection was driven by either current commercial availability or by future potential for becoming bio-derived/waste sourced (see ESI, Section 3†). The copolymerisation reactions were conducted under standard conditions to allow systematic comparisons between different monomer combinations. For the experiments where the anhydride was varied, butylene oxide (BO) was applied at [1] : [A] : [BO] = 1 : 50 : 1150, at 50 °C.^4^ These conditions are consistent with our earlier report and, given that both the iso-propoxide ligands initiate, should lead to a degree of polymerisation of 25 at complete anhydride conversion.

**Table 1:** Ring-opening copolymerisation (ROCOP) of anhydrides and epoxides with catalyst 1^a^

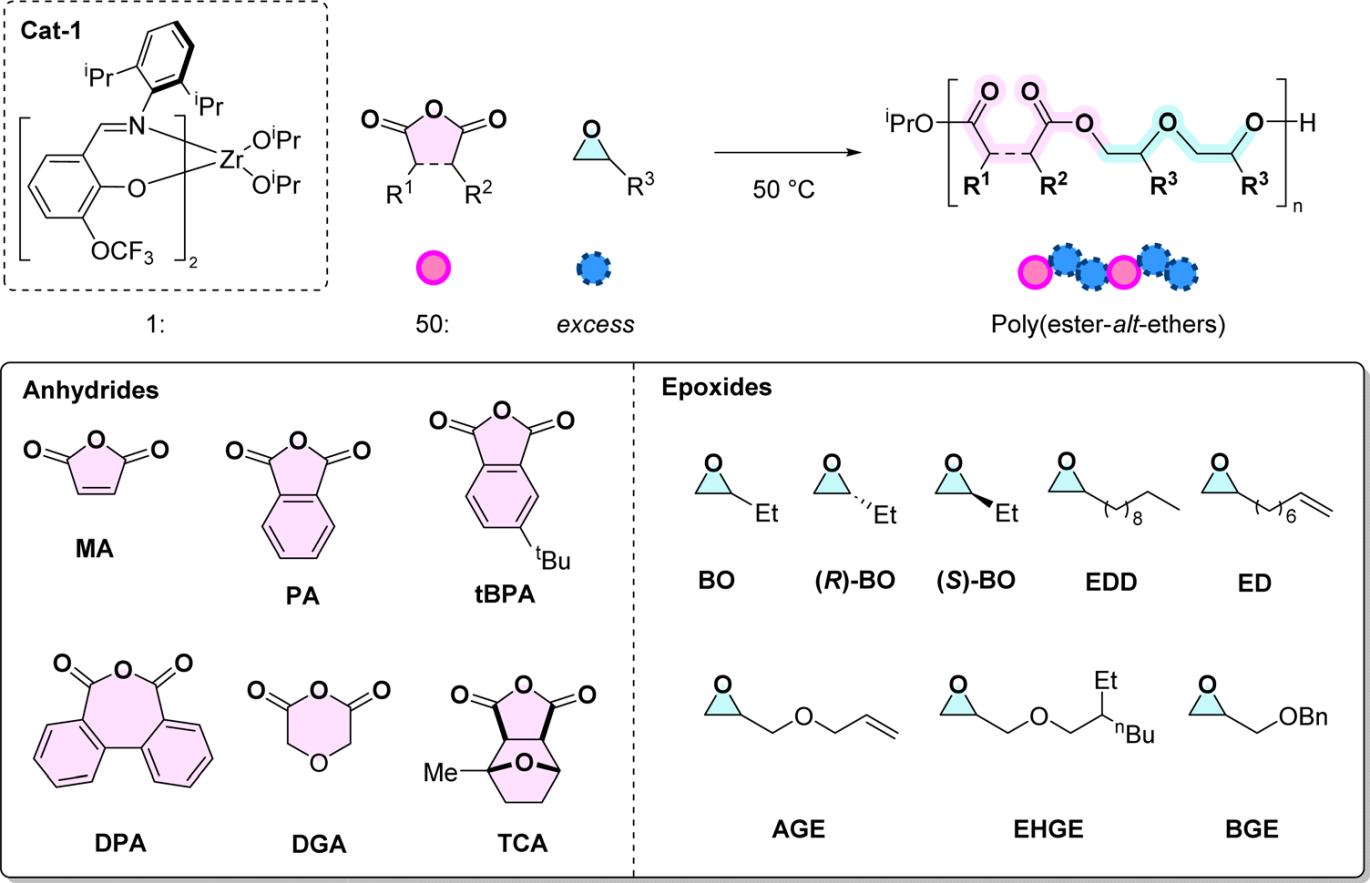								
Polymer (#)	Anhyd.	Epoxide	Degrees of polymerisation (DP): [anhyd.] : [epoxide]^b^	ABB selectivity^c^ (%)	*M*_n_ (*Đ*)^d^ [kg mol^−1^]	*M*_n_ (theo.)^e^	*k*_obs_ f (×10^−5^ M s^−1^)	*T*_g_ g (°C)
P1	MA	BO	25 : 58	95	8.4 (1.16)	6.6	9.6	−19
P2	*t*BPA	BO	25 : 58	95	6.8 (1.21)	9.2	6.4	15
P3^h^	DPA	BO	25 : 56	97	6.5 (1.36)	9.6	32.7	15
P4	DGA	BO	25 : 50	>99	9.3 (1.16)	6.6	6.1	−24
P5^i^	TCA	BO	25 : 56	96	5.4 (1.28)	8.7	1.0	9
P6	PA	BO	25 : 57	96	6.7 (1.11)	7.9	11.7	4 (ref. 20)
P7	PA	(*R*)-BO	25 : 58	95	6.1 (1.16)	7.9	14.0	−1
P8	PA	(*S*)-BO	25 : 57	96	6.4 (1.12)	7.8	14.4	−1
P9	PA	EDD	25 : 54	98	9.1 (1.18)	13.6	13.5	−37
P10	PA	ED	25 : 58	95	8.4 (1.21)	12.8	7.8	−42
P11	PA	AGE	23 : 50	97	6.0 (1.24)	9.1	0.6	−26
P12	PA	EHGE	25 : 54	98	8.8 (1.33)	13.7	2.1	−44
P13	PA	BGE	25 : 51	>99	10.3 (1.64)	12.1	0.3	0
P14	MA	ED	25 : 56	96	10.5 (1.04)	11.2	13.6	−50

a ROCOP conditions: [1] = 10 mM, [anhydride] = 0.5 M, epoxide = 1 mL, 50 °C.

b DP of monomer measured by integration of the polymer in the ^1^H NMR spectra of crude polymers against 2 iso-propoxide initiators.

c Determined as the theoretical percentage of perfect ABB epoxide selectivity (66.67%) against calculated epoxide selectivity (range = 67–70%) (Fig. S1–S66).

d Determined by gel permeation chromatography (GPC), using THF as the eluent. P1–12 and P14 calibrated using narrow MW polystyrene standards, P13 calibrated using triple detection (Fig. S67–S69).

e Theoretical *M*_n_ are calculated from the monomer conversion data and assume both iso-propoxides initiate.

f Determined from the gradient of [PA] *vs.* time (s), (Fig. S70–S72).

g Glass transition temperature obtain from differential scanning calorimetry (DSC, second heating cycle, 10 °C min^−1^ heating rate) (Fig. S73–S76).

h ROCOP conditions: [1] = 10 mM, [DPA] = 0.5 M, BO = 1 mL, 110 °C.

i ROCOP conditions: [1] = 10 mM, [TCA] = 0.5 M, BO = 1 mL, 80 °C.

To exemplify the methods used to monitor copolymerisation catalysis and characterization of the resulting copolymers, the reaction between maleic anhydride (MA) and BO is discussed here and, subsequently, the equivalent experimental data for all new copolymers is presented in the ESI† (Table 1, #1). The MA/BO copolymerisation was conducted with regular removal of aliquots which were quenched by exposure to air and the crude product conversion determined. Complete anhydride consumption was observed over 65 minutes. The new copolymer P1 showed an ABB repeat unit composition (Table 1, #1). For comparison, the perfectly alternating polyester, poly(MA-*alt*-BO), P1′ with AB sequence was also synthesised (see ESI for details, Section 4.6†). The two copolymers showed comparable molar mass values and both featured a degree of polymerisation of 25 (with respect to MA), P1*M*_n_ = 8.4 kg mol^−1^*vs.*P1′*M*_n_ = 5.4 kg mol^−1^ (Fig. S67 and S82†). It should be noted that the GPC traces for both P1, P1′ and many of the polymers in this work show a low intensity high molecular weight series due to trace quantities of diols/diacids present in the monomers. This phenomenon has been discussed in detail for AB ROCOP and does not change in ABB ROCOP.^30^

**Fig. 1:**
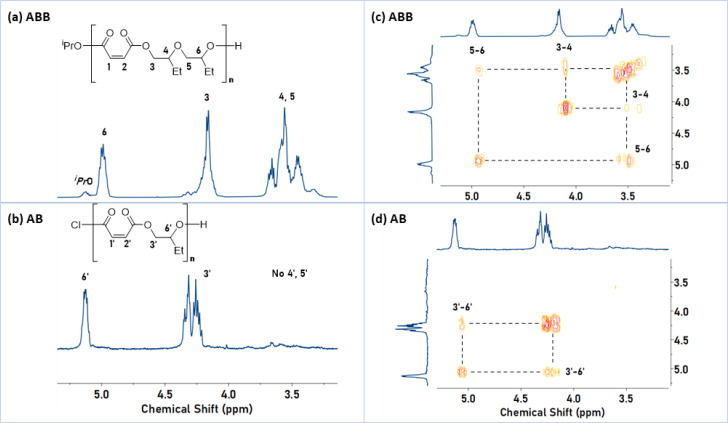
Comparison of ^1^H and COSY NMR spectra for P1 (ABB) and P1′ (AB). Left: Selected regions of the ^1^H NMR spectra (400 MHz, CDCl_3_ referenced at 7.26 ppm), illustrating the methine and methylene resonances for P1 (ABB) (a) and P1′ (AB) (b). Right: Magnified ^1^H COSY NMR spectrum of P1 (ABB) (c) and P1′ (AB) (d). Full spectra are available in Fig. S1, S3, S80 and S81.†

P1 was characterized using ^1^H NMR spectroscopy and showed diagnostic methine-ester and methylene-ester resonances at *δ*_H_ = 4.99 (proton 6) and 4.16 ppm (proton 3), respectively (Fig. 1A). It also showed signals at *δ*_H_ = 3.18–3.88 ppm (protons 4 and 5) which are in the expected region for the methylene and methine ether resonances. The ^1^H–^1^H COSY NMR spectrum showed correlations between these ester and ether protons (Fig. 1C). On the other hand, the ^1^H NMR spectrum of P1′ (AB-sequence) shows only the methine and methylene protons for the ester linkages, without any ether resonances (Fig. 1B). Its ^1^H COSY NMR spectrum shows only correlations between the two ester regions (Fig. 1D). The ^1^H and COSY NMR spectra for P1 (ABB) and P1′ (AB) exemplify how the selectivity for the ABB sequences was determined. The epoxide selectivity was determined, in each case, by comparing integrals for P(MA), *δ*_H_ = 6.20–6.30 *vs.* protons 3–6 for P1 or 3′ and 6′ for P1′. From these relative integrals (P(MA) *vs.* P(BO)) the copolymer selectivity for epoxide was calculated to be ∼70% for P1 (and around 50% for P1′). The overall ABB sequence selectivity was determined by normalising the experimental epoxide selectivity against the theoretical maximum, *i.e.* 66.67% for ABB polymers and 50% AB type polymers, resulting in a sequence selectivity for ABB linkages of 95% for P1. Selective ABB insertions, rather than random formation of ether linkages, is supported by the observation that throughout the reaction the A : B selectivity was 1 : 2 and there was no further epoxide conversion after complete anhydride consumption (Fig. S5†). These findings rule out the formation of random or block copolymers. The ^1^H DOSY NMR spectrum of P1 shows a single diffusion coefficient for all resonances, indicative of the formation of a single structure and ruling out the formation of copolymer mixtures (Fig. S6†). GPC analysis of P1 gives a molar mass of 8.4 kg mol^−1^ with a dispersity of 1.16. The calculated molar mass from ^1^H NMR spectroscopy is 7.2 kg mol^−1^ (Fig. S1†). Assuming that catalyst 1 initiates from both its iso-propoxide ligands the theoretical molar mass is 6.6 kg mol^−1^, a value which compares favourably with the experimental results. ^31^P{^1^H} NMR spectroscopy was used to characterize the chain-end groups after treatment of P1 with tetramethylethylene chlorophosphite (Fig. S7†). A set of diagnostic resonances for primary and secondary alcohol end-groups were observed. The presence of multiple resonances for both the primary and secondary alcohol is rationalised by the formation regio- and diastereoisomers of the ABB chain-end, where both epoxides are opened regio-randomly (Chart S1†). If the reaction proceeded regioselectively or formed AB polymers, only a single resonance would be expected.^31^ It is also possible that small quantities of additional ether linkages could cause multipeaks.

Both copolymers P1 and P1′ were analysed by DSC, confirming they both have amorphous structures. P1 has a lower *T*_g_ of −19 °C, whereas for P1′ the *T*_g_ is 5 °C (Fig. S73 and S83†). These values are consistent with the expected increase in chain segmental motion (backbone flexibility) for the poly(ester-*alt*-ether), P1.

Finally, a deliberately low-weight variant of P1, produced using 1 (*M*_n_ = 4.4 kg mol^−1^, see ESI Section 4.7,† polymer SP1 for further details), was analysed by matrix assisted laser desorption ionization time-of-flight spectroscopy (MALDI-ToF) (Fig. S85 and S86†). The data is fully consistent with ABB polymer sequences and there was no evidence for any AB enchainment. Further, only iso-propoxide chain end initiation was observed. Additional ether linkages were observed, and a series consistent with the composition of [^i^PrO–((MA-*alt*-BO_2_)_*n*_-*ran*-(BO)_*m*_)–H + K^+^], where *m* ranged between 0 and 4 per polymer chain. Analysis of the MALDI-ToF data indicates an approximate ABB selectivity of 94–100% consistent with the selectivity data from ^1^H NMR spectroscopy.

### Anhydrides

Following from MA/BO ROCOP, a series of other anhydrides were selected for investigation: phthalic anhydride (PA), diphenic anhydric (DPA) and 3-*tert*-butyl-phthalic anhydride (*t*BPA), diglycolic anhydride (DGA) and a tricyclic anhydride, *rac*-(3*aS*,4*R*,7*S*,7*aR*)-4-methylhexahydro-4,7-epoxyisobenzofuran-1,3-dione (TCA) (Table 1). These were selected either for interesting chemical features or for their potential future production from wastes/biomass (see ESI Section 3†). As previously described, MA and PA can be prepared from biomass, and are commonly used in ROCOP, providing unsaturation in the polymer backbone.^32^ Polycyclic monomers, including aromatic DPA and *t*BPA were examined to probe the effects of aromaticity, steric bulk and ring-size on both the catalysis and the properties of the resulting copolymers. DGA was chosen to install a fully saturated, flexible linkage into the copolymer, and delivers another ether functionality. Another potentially future bio-derived anhydride is the TCA, prepared by the reaction between MA and 2-methylfuran, followed by hydrogenation (Scheme S5†).^33^TCA provides a direct comparison to DGA as both are fully saturated but the former provides rigidity.

The reactions with these different anhydrides were each conducted using a molar ratio of [1] : [anhydride, A] of 1 : 50. Copolymerisations were conducted in neat BO, such that the overall concentration of 1 was 10 mM and the anhydride was 0.5 M. Considering the performances of the different anhydrides, *t*BPA reacted nearly equivalently to PA. The low solubility of DPA, in BO, required the reaction to be heated to 110 °C before copolymerisation occurred. Under these conditions, complete copolymerisation was achieved within 30 minutes and even at this significantly higher temperature the copolymer ABB sequence was retained. The DGA and the TCA monomers were also both successfully copolymerized with BO. The former proceeded at a comparable rate to PA, while the latter required higher temperatures (80 °C for 12 h) to reach full conversion, perhaps due to its steric hindrance.

In all cases, the catalysis was selective and furnished copolymers with ABB structures (Table 1, P1–P6). All the new copolymers were fully characterized, including by GPC and NMR spectroscopy (Fig. S1–S34, S67 and S68†). The copolymer molar mass values were in good agreement with theoretical values and all showed narrow dispersity, monomodal molar mass distributions (Table 1). ^1^H NMR spectroscopy confirmed that, in all cases, the copolymers show ABB linkage selectivity of >95%. All the copolymers have amorphous structures, as confirmed by DSC, and show *T*_g_ values which increase in the order DGA < MA < TCA ≈ PA < *t*BPA ≈ DPA, from −24 to 15 °C (Fig. S73†). The thermal stability of P1–P5, determined by TGA analysis, show degradation onsets, *T*_d,5_, from 270 to 327 °C. These copolymers therefore show relatively high thermal stabilities and broad processing temperature ranges (Table S2 and Fig. S77†).

When comparing the catalysis using the different monomers, the most reliable method to compare activity values is to determine the propagation rate constants. Pseudo zero order rate constants were determined as the gradients of linear fits to anhydride concentration *vs.* time plots. The anhydride conversions were determined by NMR spectroscopy of crude reaction aliquots (Fig. S70 and S71†). All copolymerisations showed a linear decrease in anhydride concentration over time, consistent with its zero-order dependence in the rate law. Linear fits to the data gave rate constants, *k*_obs_, which allowed for understanding of the influences on catalytic activity using the different monomers. For the series of anhydrides, the rates decrease in the order MA > *t*BPA ≈ PA ≈ DGA. Since the tricyclic monomers, DPA and TCA, were copolymerized at higher temperatures direct rate comparisons are not appropriate.

The regioselectivity of the ABB repeating unit of P6 was determined (Table S1 and Fig. S23–S34†). Using a combination of ^1^H and ^13^C, 1D and 2D NMR techniques it was found that the ring-opened PA (A) was adjacent to both methine and methylene protons in approximately equal proportions (Fig. S29 and S30†). Thus, the two ring-opened epoxides within each ABB unit (*i.e.*BB) are inserted randomly, *i.e.* [H–T] : [H–H] : [T–T] is 50 : 30 : 20 (theoretical ratios are 50 : 25 : 25). When this result is combined with the end-group analysis of P1 (Fig. S7†), it reveals that the epoxide enchainment is regiorandom.

### Epoxides

In comparing the influence of the epoxides, 8 representative monomers were evaluated: butylene oxide (BO),^20^ (*R*)-butylene oxide ((*R*)-BO), (*S*)-butylene oxide ((*S*)-BO), 1,2-epoxydodecane (EDD), 1,2-epoxydecene (ED), allyl glycidyl ether (AGE), 2-ethyl hexyl glycidyl ether (EHGE) and benzyl glycidyl ether (BGE) (Table 1, P6–P14). Experiments were conducted using the standard conditions outlined earlier and with phthalic anhydride (PA) as the co-monomer. The epoxides were selected to deliver copolymers with aliphatic and flexible backbones, targeting materials with low glass transition temperatures since these are well matched for applications in liquid formulations, as plasticizers, electrolytes and inks.^27,34^ Further, to deliver functionalised copolymers, monomers featuring alkene substituents were explicitly targeted. Such alkene groups are already known to be useful in post-polymerisation functionalization, for example copolymers from AGE are common in adhesives and sealants due to the ability to cross-link the alkene groups.^35^ The reactions with enantiopure monomers were undertaken to evaluate the potential, if any, to deliver semi-crystalline copolymers.^36^ Although at present the epoxides employed in this study are all petrochemicals, there are routes to produce them from renewables in future (see Schemes S6–S12† for further details).

In all cases, anhydride (A) and epoxide (B) ROCOP yielded copolymers with ABB sequences (>95% by NMR spectroscopy). The copolymers showed consistent degrees of copolymerisation of 25, with molar mass values from 3.7 to 9.1 kg mol^−1^ and narrow dispersity distributions. The copolymerisation using BO, (*R*)-BO or (*S*)-BO showed equivalent rates and selectivity to the racemic epoxide (BO), producing P6, P7 and P8, respectively (Table 1). The copolymers of P7 and P8 show specific rotations for P7 [*α*]^25^_D_ = −1.59, *c* 0.061 and for P8 [*α*]^25^_D_ = +1.43, *c* 0.065 (in CDCl_3_). In both cases, the copolymers were amorphous, DSC did not show any melting or crystallization transitions, and *T*_g_ values were very close to the value for racemic P6 (*T*_g_ for P6 = 4 °C, P7 = −1 °C and P8 = −1 °C, Fig. S74†). The copolymer from EDD, P9, was also amorphous with a low *T*_g_ value of −37 °C (Table 1, #9). The related copolymer, P10, prepared using ED, also showed an even lower glass transition temperature (−42 °C) and features an alkene moiety (Table 1, #10).^37^ All the glycidyl epoxides, *i.e.*, AGE, EHGE and BGE, were successfully copolymerized to yield ABB copolymers, P11–P13. These glycidyl ethers showed significantly lower rates than equivalent reactions using alkylene oxides, with complete conversion requiring 8–96 hours instead of ∼2 hours. Within the series of poly(ester-*alt*-ethers) prepared from different epoxides (with PA as anhydride), all materials were amorphous, the *T*_g_ values increase in the order EHGE ≈ EDD ≈ ED < AGE < (*S*)-BO/(*R*)-BO ≈ BO ≈ BGE (−44 to 4 °C). In addition, all of the polymers showed *T*_d,5_ values from 283 to 344 °C (Table S2, Fig. S78 and S79†).

Comparing the rate constants, *k*_obs_, for the copolymerisation (determined as gradients of linear fits to anhydride conversion *vs.* time data) allows insight into monomer structure–activity effects. The glycidyl epoxides show significantly lower rates than the alkylene oxides (Fig. S71 and S72†). For example, P6 shows a rate which is around 20× higher than AGE (P6*k*_obs_ = 11.7 × 10^−4^ M s^−1^; P11*k*_obs_ = 0.6 × 10^−4^ M s^−1^). The slower rates are tentatively attributed to electronic deactivation of the glycidyl epoxide rings *vs.* the aliphatic epoxide as reported by others.^38,39^EHGE shows slightly higher rates than both BGE and AGE but still lower than that of BO suggesting a potential steric and electronic interplay (*k*_obs_ in the order of aliphatic epoxides < EHGE < AGE ≈ BGE). Unlike the rest of the series, P13, derived from BO and BGE was analysed using a triple-detection GPC to obtain an accurate molar mass (*M*_n,GPC_ = 10.2, *Đ* = 1.64, see ESI† for details). It's possible that BGE contains a higher quantity of protic impurities accounting for the broader molecular weight distribution.^40^

To target ABB copolymers featuring alkene substituents on both monomers, MA and ED were polymerized (Table 1, #14). The copolymer, P14, shows the desired ABB sequence and has a low *T*_g_ of −50 °C and *T*_d,5_ = 344 °C (Fig. S76 and S79†).

### Catalyst tolerance

The previously reported yield for the synthesis of catalyst 1 was 22% over two steps, with the low isolated yield resulting from two recrystallisation steps.^20^ In efforts to improve its yield, HL_1_ was purified by column chromatography which allowed its isolation in 89% yield (see ESI Section 4.8†). Catalyst 1 was isolated after complete conversion of HL_1_ by evaporating the reaction mixture to dryness (1 × 10^−3^ mbar overnight) and this approach yielded catalyst 1 in >99% conversion and 89% isolated yield. This ‘crude’ catalyst 1 was used to synthesis polymer P6 in an analogous manner to Table 1 (Table S4,† polymer SP2). In both cases the ABB selectivity was ∼96% and the polymer molar mass for SP2 (*vs.*P6) was 4.9 kg mol^−1^ (*vs.* 6.7). This result indicates that for comparisons of catalyst rate and selectivity it is best to use the recrystallized catalyst 1. However, to produce larger quantities of ABB polymer, “crude” catalyst 1 is quite suitable and recommended.

In order to test the tolerance of the catalyst at lower loadings, 3 reactions to target higher weight polymers were undertaken (Table S5,†SP3–5). Monomers PA and BO were used at loadings of [1] : [BO] : [PA] at 1 : 250–500 : 2925–5557, where [1] = 0.25–0.5 mM, an order of magnitude more dilute than the standard testing conditions (Table 1). In this regime the catalysts still operated with excellent ABB selectivity (∼97%) and reached full conversion of anhydride between 18 and 48 hours. The polymers show high molar masses, *M*_n_ = 31–46 kg mol^−1^ (Fig. S88†). In each case, the theoretical molar mass was higher than the observed molar mass, calculated against polystyrene standards, giving bimodal GPC traces. This is likely due to the effect of residual diols/diacids in the monomers exerting a more significant influence at lower catalyst loadings.^41^

### Cyclic ethers

**Scheme 2:**
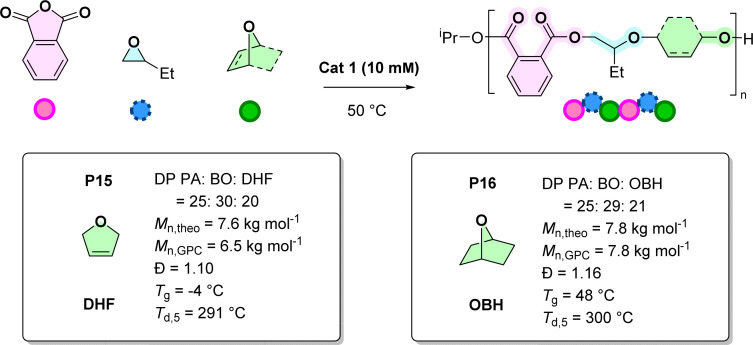
Ring-opening copolymerisation (ROCOP) of PA, BO and cyclic ethers with catalyst 1. ROCOP conditions for P15: [1] = 10 mM, [PA] = 0.5 M, BO = 0.25 mL, 2.8 M, DHF = 0.75 mL, 9.9 M, total volume of BO + OBH = 1 mL, 50 °C. ROCOP conditions for P16: [1] = 10 mM, [PA] = 0.5 M, BO = 0.25 mL, 2.8 M, OBH = 0.75 mL, 7.4 M, total volume of BO + OBH = 1 mL, 50 °C.

Our prior report demonstrated that catalyst 1 could also enchain some tetrahydrofuran (THF) or bio-derived 2-methyl tetrahydrofuran (MeTHF) into poly(ester-*alt*-ethers).^20^ In PA (A), BO (B) and THF (C) ROCOP the resulting copolymers showed mixtures of ABB and ABC sequences. Preceding this discovery, there was only a single example of successful anhydride/THF ROCOP using high loadings of a super-acid, bistriflimidic acid, catalyst operating at high temperatures (TOF = 3–10 h^−1^, [cat.] : [THF] : [anhydride] 1 : 20 : 20, 130 °C).^42^ Here, the goal is to evaluate the Zr-catalyst in anhydride (A)/epoxide (B)/cyclic ether (C) ROCOP. The copolymerisations were conducted using [1] : [PA] : [BO] : [cyclic ether] of 1 : 50 : 288 : 740–992, where catalyst and anhydride concentration were 10 mM and 0.5 M, respectively, (BO = 0.25 mL, cyclic ether = 0.75 mL and total volume = 1 mL) (Scheme 2). In testing the cyclic ether monomer scope, first, 2,5-dihydrofuran (DHF) was targeted since it has an internal alkene group which should facilitate post-polymerisation functionalization and can be synthesised from sugars (Scheme S13†).^43^ The copolymerisation of PA, BO and DHF was successful producing copolymer P15 with a molar mass value of 6.5 kg mol^−1^ and a narrow dispersity (*Đ* = 1.10, Fig. S97†). ^1^H NMR spectroscopy reveals that the ether linkages comprise 60% BO and 40% DHF units (Fig. S89†). These ether linkage compositions are similar to those reported for PA, BO and THF ROCOP using the Zr-catalyst.^20^ The other spectroscopic data are also fully consistent with the formation of a poly(ester-*alt*-ethers) with ABB/ABC sequences. The new copolymer, P15, is amorphous, with a *T*_g_ = −4 °C, and shows reasonable thermal stability, with *T*_d,5_ of 291 °C (Fig. S99 and S100†). Next, 7-oxabicyclo-[2.2.1] heptane (OBH), which is a strained bicyclic ether, was investigated (Schemes 2 and S14†). OBH, was previously used in cationic ether (co)-polymerisations but has not been previously applied in anhydride/epoxide ROCOP.^44,45^ The copolymerisation of PA, BO and OBH yielded the new poly(ester-*alt*-ether) featuring ABB/ABC sequences, P16. The copolymer shows 58% BO and 42% OBH linkages (from ^1^H NMR spectroscopy, Fig. S93†). It has an amorphous structure with a glass transition temperature of 48 °C (Fig. S99†), consistent with the rigidity imparted by the cyclohexane units, as well as good thermal stability, with *T*_d,5_ of 300 °C (Fig. S100†).

### Functionalized copolymers

**Scheme 3:**
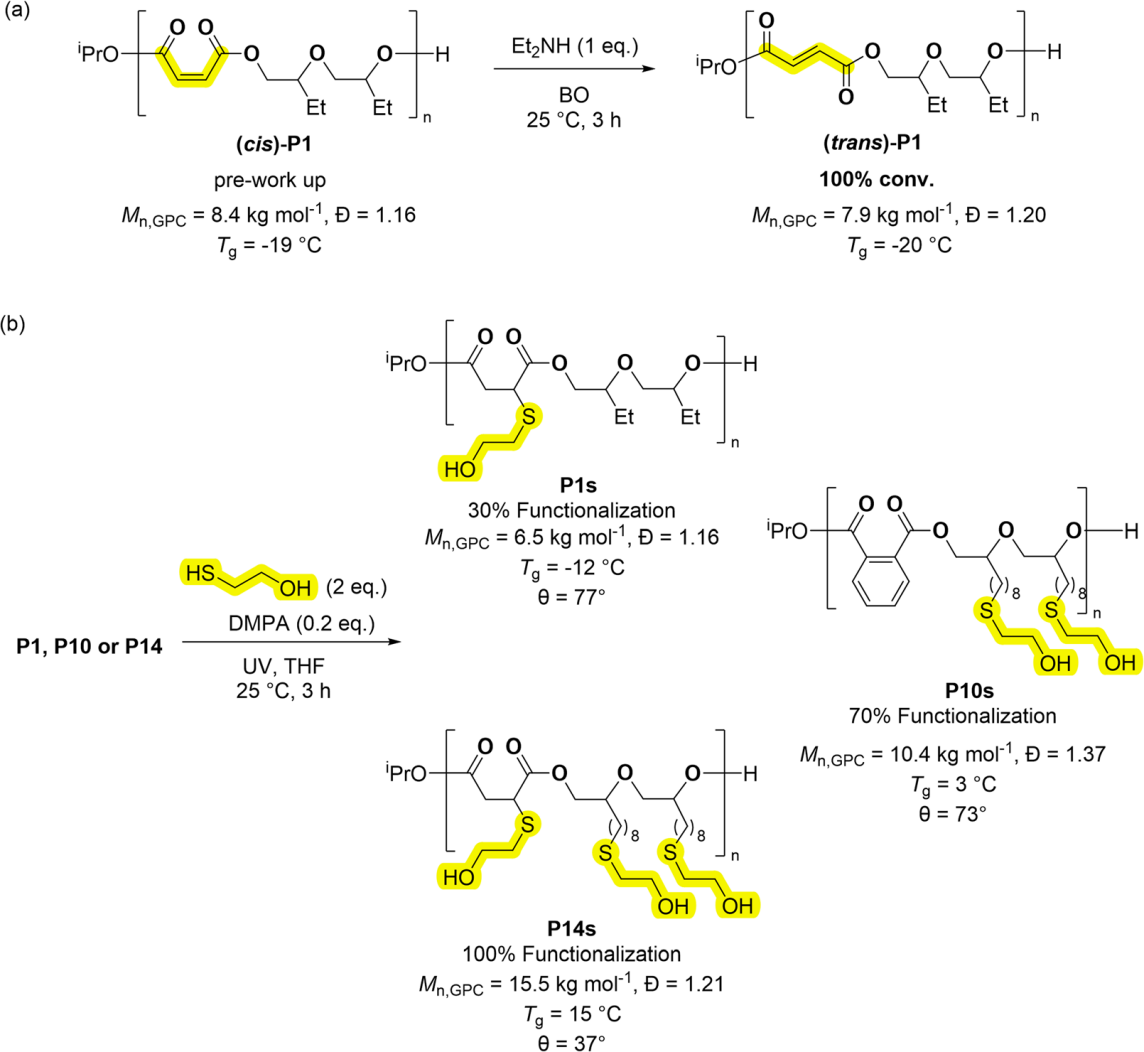
Post-copolymerisation functionalization of poly(ester-*alt*-ethers) (ABB). (a) Isomerization of the alkene moieties in P1; (b) processes to install hydroxyl groups, using thiol–ene reactions, onto the polymer repeat units. These apply the copolymers, P1, P10, P14, to achieve 30, 70 and 100% hydroxyl substitution, respectively (where % refers to the extent of hydroxyl substitution of both A and B repeat units). Note that the thiol–ene reactions are regio-random.

Many applications would benefit from the ability to install functional substituents, for example to moderate solubility, impart pH or chemical responses or to control degradation rates. The series of poly(ester-*alt*-ethers) feature several polymers with alkene substituents. Copolymers derived from maleic anhydride feature *cis*-alkene groups in the copolymer backbone, if these are isomerized, they produce repeat units of fumaric acid (FA). Alternatively copolymers containing FA require the use of step-growth copolymerisation which is not typically very well controlled.^46^ Coates and co-workers pioneered the epoxide/maleic anhydride ROCOP, followed by alkene isomerization, to access polyesters featuring FA repeat units.^47^ In a similar manner, copolymer P1 was treated with an equivalent of HNEt_2_ (per alkene moiety) to yield FA (*trans*)-P1 in quantitative yield (Scheme 3A).^47^ The new copolymer showed an amorphous structure and nearly equivalent *T*_g_ to P1 (P1 = −19 °C, (*trans*)-P1 = −20 °C, Fig. S83†). One attraction of fumaric acid repeat units is the future potential for these polymers in biomedical applications. Fumaric acid has a considerably higher lethal dose, when administered orally to rats, than maleic acid (FA, LD_50_ = 10.1 g kg^−1^; MA, LD_50_ = 0.7 g kg^−1^).^48,49^ Further, poly(ethylene fumarate) was more readily biodegraded than poly(ethylene maleate).^50^ Alkene groups can also be further reacted, by additions, to install functional substituents and the thiol–ene reaction is interesting due to its high yield.^37^ The investigation into this process focused on three copolymers, P1, P10, P14, featuring different numbers of alkenes in the repeat units, 30, 70 and >99% unsaturation as defined by the % alkene groups in each repeat unit (Scheme 3). Each copolymer was reacted with 2-mercaptoethanol (2 equivalents per alkene) and with the photo-initiator DMPA (2,2-dimethoxy-2-phenylacetophenone, 0.2 equivalents), in THF at 25 °C and using UV irradiation. The copolymers were all successfully functionalised with alcohol groups, as determined from the ^1^H NMR spectra which showed complete alkene resonance consumption (*δ*_H_ ≈ 6.50–5.50 ppm) from MA and/or ED (Fig. S105–S107†). The spectra also showed new resonances consistent with the thio–ether protons (*δ*_H_ ≈ 2.40–2.75 ppm). GPC analyses showed a slight increase in the molar mass for P10s and P14s, consistent with the addition of the hydroxyl groups, the *T*_g_ values changed from −50 to −12 °C for P1–P1s, −42 to 3 °C for P10–P10s, and −48 to 15 °C for P14–P14s (Fig. S108 and S109†). The functionalised polymers showed reduced water contact angles (*θ*), by 19, 25 and 51° for P1s, P10s and P14s respectively (Table S9†). These results demonstrate the expected increase in hydrophilicity as hydroxy functionalisation increases. This property is useful both for surfactant application and to increase degradation rates.

## Conclusions

A Zr(iv) catalyst was able to produce novel poly(ester-*alt*-ethers) from mixtures of several different epoxides, anhydrides and cyclic ethers. This catalysis was used to produce a series of 19 new copolymers which were fully characterised using spectroscopy, GPC and thermal methods. By selecting the various monomer combinations, it was possible to control the copolymer's glass transition temperatures and, through post-functionalization reactions, to install pendant hydroxyl groups at regular sites along the copolymer backbone to increase the hydrophilicity of the polymers. The new polymerisation catalysis should be exploited, in future, to prepare degradable polymers for liquid formulations, electrolytes, elastomers, coatings and adhesives.^6^

## Data availability

The data supporting this article have been included as part of the ESI.†

## Author contributions

RWFK: conceptualization, investigation, methodology, writing original draft, writing editing and review, AC: investigation, methodology, funding acquisition, writing original draft, CKW: conceptualization, investigation, methodology, funding acquisition, writing original draft, writing review and editing, supervision.

## Conflicts of interest

There are no conflicts to declare.

## Supplementary Material

SC-015-D4SC02051K-s001
