# Supplementary material for: Cyclic ether and anhydride ring opening copolymerisation delivering new ABB sequences in poly(ester-alt-ethers)
Source: Chem Sci. 2024 Jun 27;15(29):11617–25. doi: 10.1039/d4sc02051k (PMC11268503; doi:10.1039/d4sc02051k)
Supplement: SC-015-D4SC02051K-s001 [file SC-015-D4SC02051K-s001.pdf]

Electronic Supplementary Material (ESI) for Chemical Science.
This journal is © The Royal Society of Chemistry 2024

Supporting Information

Cyclic Ether and Anhydride Ring Opening Copolymerizations Delivering New ABB Sequences in Poly(Ester-*alt*-Ethers)

Ryan W. F. Kerr,[§] Alexander R. Craze,[§] Charlotte K. Williams*

Department of Chemistry, University of Oxford, Chemistry Research Laboratory, 12 Mansfield Road, Oxford OX1 3TA, UK

Contents

1.0 General Methods 4

1.1 Materials 4

1.2 Methods 4

2.0 Ring-Opening Copolymerizations (ROCOP) of Anhydrides, Epoxides and Cyclic Ethers 6

2.1 Copolymerization Methods 6

2.1.1 ROCOP of Anhydrides and Epoxides: 6

2.1.2 ROCOP of Anhydride, Epoxide and Cyclic Ethers: 6

3.0 Potential Routes from Biomass to the Monomers 7

3.1 Phthalic Anhydride and Maleic Anhydride 8

3.2 4-*tert*-Butylphthalic Anhydride 8

3.3 Diphenic Anhydride 8

3.4 Diglycolic Anhydride 9

3.5 *rac*-(3a*S*,4*R*,7*S*,7a*R*)-4-Methylhexahydro-4,7-epoxyisobenzofuran-1,3-dione (TCA) 9

3.6 Butylene Oxide 9

3.7 1,2-Epoxydodecane 10

3.8 1,2-Epoxy-9-dodecene 10

3.9 Epichlorohydrin 10

3.10 Allyl Glycidyl Ether 11

3.11 2-Ethylhexyl Glycidyl Ether 11

3.12 Benzyl Glycidyl Ether 11

3.13 2,5-Dihydrofuran 11

3.14 7-Oxabicyclol-[2.2.1] heptane 12

4.0 Characterization Data for Polymers Described in Table 1 13

4.1 ^{1}H NMR Spectra for Polymers Described in Table 1 13

4.1.1 NMR Spectra for P1 13

4.1.1.1 End-group Analysis of P1 16

4.1.2 NMR Spectra for P2 17

4.1.3 NMR Spectra for P3 19

4.1.4 NMR Spectra for P4 21

4.1.5 NMR Spectra for P5 23

4.1.6 NMR Spectra for P6 25

4.1.6.1 Regioselectivity of P6 28

4.1.7 NMR Spectra for P7 33

4.1.8 NMR Spectra for P8 35

4.1.9 NMR Spectra for P9 37

4.1.10 NMR Spectra for P10 39

4.1.11 NMR Spectra for P11 41

4.1.12 NMR Spectra for P12 43

4.1.13 NMR Spectra for P13 45

4.1.14 NMR Spectra for P14 ... 47
4.2 GPC Data for Polymers Described in Table 1 ... 49
4.3 Kinetic Data for Polymers Described in Table 1 ... 52
4.4 DSC Data for Polymers Described in Table 1 ... 55
4.5 TGA Data for Polymers Described in Table 1 ... 57
4.6 Data for P1' ... 61
4.7 MALDI-ToF Data for SP1 ... 64
4.7.1 Synthesis of SP1 ... 64
4.8 Comparison of P6 Synthesized from Recrystallized and "Crude" Catalyst 1 ... 67
4.8.1 Alternative Synthesis of 1 ... 67
4.9 Synthesis of Higher Weight P6 ... 69
5.0 Characterization Data for Polymers Described in Scheme 2 ... 70
5.1 NMR Spectra for Polymers Described in Scheme 2 ... 70
5.1.1 NMR Spectra for P15 ... 70
5.1.2 NMR Spectra for P16 ... 72
5.2 GPC Data for Polymers Described in Scheme 2 ... 74
5.3 Kinetic Data for Polymers Described in Scheme 2 ... 74
5.4 DSC Data for Polymers Described in Scheme 2 ... 75
5.5 TGA Data for Polymers Described in Scheme 2 ... 75
6.0 Characterization Data for Polymers Described in Table 1 ... 76
6.1 Data for Polymers Described in Scheme 3 – Post-Polymerization Functionalization ... 76
6.1.1 Procedure for the Synthesis of (*trans*)-P1 ... 76
6.1.2 General Procedure for 2-Mercaptoethanol Functionalization ... 76
6.2 NMR Spectra for (*trans*)-P1 ... 77
6.3 NMR Spectra for Polymers Described in Table S3 and Scheme 3 – Post-Polymerization Functionalization ... 79
6.3.1 NMR Spectra for P1s ... 79
6.3.2 NMR Spectra for P10s ... 80
6.3.3 NMR Spectra for P14s ... 81
6.4 GPC Data for Polymers Described in Table S3 and Scheme 3 – Post-Polymerization Functionalization ... 82
6.5 DSC Data for Polymers Described in Scheme 3 and Table S3 ... 83
6.6 TGA Data of P1s, P10s and P14s ... 84
6.6 Data of P1, P10, P14, P1s, P10s and P14s ... 85

1.0 General Methods

1.1 Materials

All air-sensitive and moisture-sensitive reactions (synthesis of **1**, monomer purifications, and polymerizations) were carried out under inert conditions, using standard Schlenk techniques and in a N_2-filled glovebox. Catalyst **1** was synthesised using literature procedures unless otherwise stated.[1] Butylene oxide (BO), 1,2-epoxydodecane (EDD), 1,2-epoxy-9-decene (ED), allyl glycidyl ether (AGE), 2-ethyl hexyl glycidyl ether (EHGE) and benzyl glycidyl ether (BGE) were dried by stirring them over CaH_2, followed by fractional distillation. Next, the epoxides were stirred over BuLi, followed by a second and third series of fractional distillations. The monomers were de-gassed, by freeze-pump-thaw in triplicate, and stored under an inert (N_2) atmosphere. (*R*)-Butylene oxide ((*R*)-BO) and (*S*)-butylene oxide ((*S*)-BO), were dried by stirring over CaH_2, followed by a fractional distillation. The monomers were de-gassed, by freeze-pump-thaw in triplicate, and stored under an inert (N_2) atmosphere. Phthalic anhydride (PA) was purified by extraction using toluene, followed by recrystallization from a chloroform solution and sublimation (three times). It was stored under an inert (N_2) atmosphere. *tert*-Butyl phthalic anhydride (tBPA) and diphenic anhydride (DPA) were isolated by sublimation and stored under an inert (N_2) atmosphere. Tricyclic anhydride, *rac*-(3aS,4R,7S,7aR)-4-methylhexahydro-4,7-epoxyisobenzofuran-1,3-dione (TCA) was synthesised using literature procedures, purified by sublimation and stored under an inert (N_2) atmosphere.[2] Diglycolic anhydride (DGA) was purified by two recrystallizations from acetic anhydride and sublimation in triplicate, being stored under an inert (N_2) atmosphere. Maleic anhydride (MA) was purified by recrystallization from chloroform and sublimation in triplicate and stored under an inert (N_2) atmosphere.

1.2 Methods

NMR Spectroscopy: ^{1}H, ^{13}C and ^{31}P spectra were obtained using Bruker AV 400 MHz and 500 MHz instruments. In order to establish reproducible analysis of crude and pure polymers by ^{1}H NMR spectroscopy, sample **P1** was measured with a D_1 = 1 s, 15 s and 60 s. No differences between relative aryl/aliphatic integrals were found, therefore D_1 = 1 s was used for all further ^{1}H NMR spectroscopy.

Gel Permeation Chromatography (GPC):

Polymer analysis was carried out using a Shimadzu LC-20AD instrument, equipped with PSS SDV 5 µm pre-column and two PSS SDV 5 µm linear M columns and a Refractive Index (RI) detector. Samples were dissolved in HPLC grade THF, filtered through 0.2 µm PTFE filters (VWR) and measurements were determined at 1 mL min^{-1} flow rate, at 30 °C. Monodisperse polystyrene standards were used to calibrate the instrument.

Polymer analysis of **P13** was carried out using a Agilent LC1260 Infinity II System fitted with a PLgel 5 µm (50 x 7.5 mm) guard column and two PLgel 5 µm MIXED-C (300 x 7.5 mm) analytical columns and equipped with a multi-detector suite (MDS) comprising a dual-angle light scattering detector (LS, 15 & 90 degrees), refractive index detector (RI), and viscometer (VS). THF (FisherScientific, GPC grade stabilized with 0.025% BHT) was used as the eluent with a flow rate of 1 mL min^{-1} at 35 °C. The system was calibrated using a set of narrow polystyrene standards (Agilent EasiVial PS-H 2 mL) for standard GPC calibration and a narrow polystyrene standard (M_p =

29,510 g/mol, Mw,LS = 29,810 g/mol, dn/dc = 0.185, M_w/M_n = 1.02, [η] = 0.1777 dL/g) for system calibration and triple detection.

Differential Scanning Calorimetry (DSC): was performed using a DSC25 (TA Instruments). A sealed, empty crucible was used as a reference, and the instrument was calibrated using zinc and indium samples. Polymer samples were heated from 40 °C to 100 °C, at a rate of 10 °C min^{-1} and under a N_2 flow (80 mL min^{-1}). Samples were subsequently cooled to -100 °C, at a rate of 10 °C min^{-1}, and kept at -100 °C for a further 5 minutes, followed by a heating-cooling cycle from -100 °C to 100 °C, at a rate of 10 °C min^{-1}. Each sample was analyzed over two heating-cooling cycles. Glass transition temperatures (T_g) are reported as the midpoint of the transition taken from the second heating cycle.

Thermal Gravimetric Analysis (TGA): Experiments were conducted using a TGA5500 System (TA Instruments), equipped with the TRIOS software package. Polymer samples were heated from 40 to 100 °C, at a rate of 10 °C min^{-1}, under a N_2 flow (100 mL min^{-1}). Samples were held at 100 °C for 30 mins, to remove any minor quantities of residual solvent, and cooled to 40 °C. Polymer samples were analysed by heating from 40 to 600 °C, at a rate of 10 °C min^{-1}, under a N_2 flow (100 mL min^{-1}).

MALDI-ToF Mass Spectrometry: MALDI-TOF analyse was carried out on a Bruker Autoflex Speed MALDI-TOF. Spectra were acquired in positive-linear mode.

Water Contact Angle Measurements: Static water contact angles were measured using a Drop Shape Analysis System (Tracker, ITConcept, france). A 5 µL drop of ultra-pure water (MilliQ water, Millipore, MA, USA) was placed on the polymer film surfaces and the static water contact angle was measured. The measurements were performed on multiple different areas of each slide and these values were averaged and the standard deviation of these measurements was recorded.

2.0 Ring-Opening Copolymerizations (ROCOP) of Anhydrides, Epoxides and Cyclic Ethers

2.1 Copolymerization Methods

2.1.1 ROCOP of Anhydrides and Epoxides:

Typical ROCOP procedure: In a glovebox, catalyst **1** (9.4 mg, 0.01 mol) was weighed into a vial, then dissolved/suspended in 1 mL of epoxide. Anhydride (0.5 mmol) was added to the reaction mixture, the vial sealed with electric tape, then parafilm, removed from the glovebox and heated to the stipulated temperature (generally 50 °C, or higher for reactions involving TCA and DPA). After the desired time, samples were cooled to 0 °C, taken into the glovebox for removal of aliquots (20 µL), used to determine conversion data, or exposed to air to quench and evaporated to dryness. The crude polymer was characterized at ~10 mg mL^{-1} (THF solution) for GPC and ~10 mg mL^{-1} ($CDCl_3$ solution) for ^{1}H NMR spectroscopy. The pure polymers were obtained by precipitation from methylene chloride solutions, using methanol or pentane as non-solvents, and by drying under vacuum at 60 °C, overnight.

2.1.2 ROCOP of Anhydride, Epoxide and Cyclic Ethers:

Typical polymerization procedure: In a glovebox, catalyst **1** (9.4 mg, 0.01 mol) was weighed into a vial, then dissolved/suspended in 0.25 mL of butylene oxide and 0.75 mL of the selected cyclic ether. Phthalic anhydride (74 mg, 0.5 mmol) was added to the reaction mixture, the vial sealed with electric tape, then parafilm, removed from the glovebox and heated to 50 °C. After the desired time, samples were cooled to 0 °C, taken into the glovebox for removal of aliquots (20 µL) used for conversion data, or exposed to air to quench and evaporated to dryness. The crude polymer was characterized at ~ 10 mg mL^{-1} (THF solution) for GPC and ~ 10 mg mL^{-1} ($CDCl_3$ solution) for ^{1}H NMR spectroscopy. The pure polymers were obtained by precipitation from methylene chloride solutions, using methanol or pentane as the non-solvents, and after drying under vacuum, at 60 °C, overnight.

3.0 Potential Routes from Biomass to the Monomers

The following synthetic routes exemplify the potential to source the monomers from biomass. Each reaction is substantiated using a literature report or patent with references given therein. In circumstances where a particular reaction is not yet reported, a related synthesis is referenced.

Previous reports describe MA and PA preparation from biomass, including cellulose (Scheme S1).[3] Polycyclic monomers, including aromatic diphenic anhydride (DPA) and 4-*tert*-butylphthalic anhydride (tBPA) can potentially be sourced from cellulose, glycerol and other biomass materials (Scheme S2-3). The precursor of DGA, diglycolic acid, has been detected in very low quantities as a by-product in the enzymatic production of lysine (Scheme S4),[4] nevertheless it is currently synthesised at scale by multiple oxidation steps starting from ethylene via ethylene oxide and diethylene glycol.[5-7] Recent advances in producing ethylene glycol from cellulose, combined with established routes to ethene from carbohydrates, could allow for a renewable route to DGA in the future.[8] Another future bio-derived anhydride is the TCA, prepared by the reaction between MA and 2-methylfuran, followed by hydrogenation (Scheme S5).[9]

The industrial precursor to butylene oxide (BO), 1,2-butanediol,[10] has the potential to synthesised from the naturally occurring sugar erythritol,[11] which itself can be sourced from glycerol (Scheme S6).[12] The further long chain aliphatic epoxides, 1,2-epoxydodecane (EDD)[13-16] and 1,2-epoxydecene (ED)[17-21] could be sourced in multi-step processes from vegetable or castor oil, respectively (Scheme S7-8). Epichlorohydrin (ECH), the industrial glycidyl oxide precursor, could be sourced from glycerol (Scheme S9).[22] The glycidyl oxides would be prepared by treatment of ECH with a suitable alcohol.[23] The relevant alcohol precursors to this study, allyl alcohol (for AGE),[24] 2-ethyl hexanol (for EGHE)[25] and benzyl alcohol (for BGE)[26] could also be derived from renewable feedstocks (Schemes S10-12).

Hydrofurans are also capable of being sourced from bio-renewable routes. For example, 2,5-dihydrofuran (DHF), which contains a valuable alkene moiety, can be directly formed from the naturally occurring sugar erythritol (Scheme S13).[27] 1,4-Dimethoxybenzene, a precursor to bicyclic compound, 7-Oxabicyclol-[2.2.1] heptane (OBH), could be sourced from willow (Scheme S14).[28] The isolated 1,4-dimethoxybenzene could then undergo a series of hydrogenation steps, *via* the cyclization of 1,4-cyclohexanediol to access OBH.[29-31]

3.1 Phthalic Anhydride and Maleic Anhydride

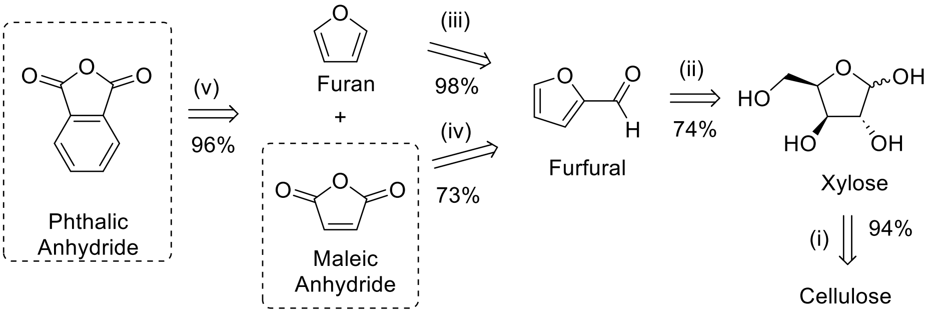

Scheme S1: Potential renewable synthesis of phthalic anhydride (PA) and maleic anhydride (MA). (i) Mäki-Arvela *et al.*[32] (ii) Gokhale *et al.*[33] (iii) Stevens *et al.*[34] (iv) Alonso-Fagúndez *et al.*[35] (v) Mahmoud *et al.*[36]

3.2 4-*tert*-Butylphthalic Anhydride

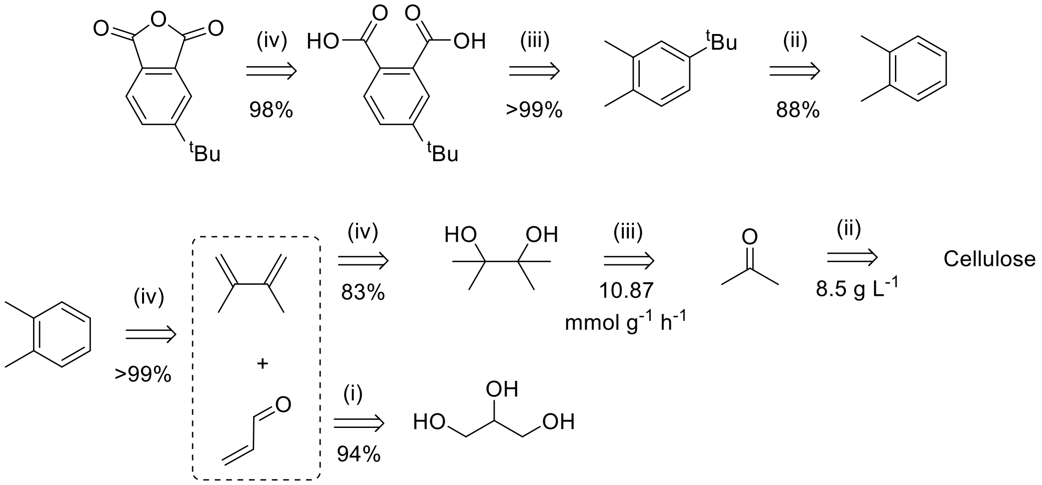

Scheme S2: Potential renewable synthesis of 3-tert-Butyl-phthalic anhydride (tBPA). (i) Alhanash *et al.* [37] (ii) Luo *et al.*[38] (iii) Cao *et al.*[39] (iv) Hu *et al.*[40] (v) Pan *et al.*[41] (vi) Libing *et al.*[42] (vii) Hanack *et al.*[43]

3.3 Diphenic Anhydride

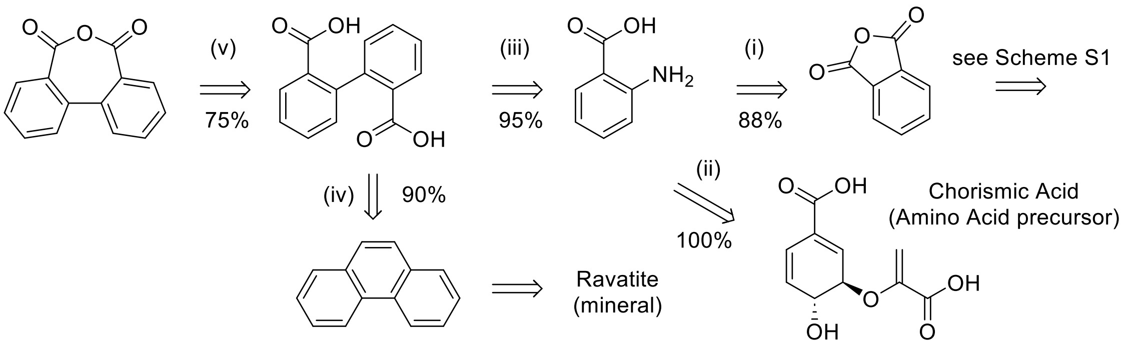

Scheme S3: Potential renewable synthesis of Diphenic Anhydride (DPA) (i) Maki *et al.*[44] (ii) Ziebart *et al.*[45] (iii) Samadi *et al.*[46] (iv) Han *et al.*[47] (v) Tarasenko *et al.*[48]

3.4 Diglycolic Anhydride

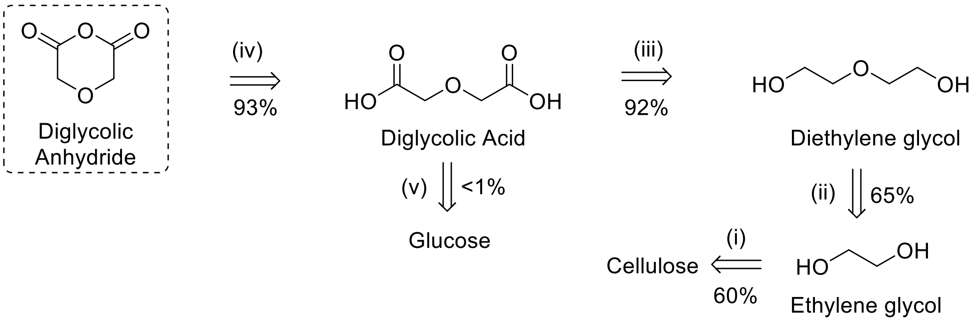

Scheme S4: Potential renewable synthesis of diglycolic anhydride (DGA). (i) Wang *et al.*[8] (ii) Rohand *et al.*[49] (iii) Gur'eva *et al.*[50] (vi) Kantin *et al.*[51] (v) Kind *et al.* It is possible that future bio-technology routes could also be developed since diglycolic acid was a by-product in the enzymatic synthesis of lysine from D-glucose.[4]

3.5 *rac*-(3a*S*,4*R*,7*S*,7a*R*)-4-Methylhexahydro-4,7-epoxyisobenzofuran-1,3-dione (TCA)

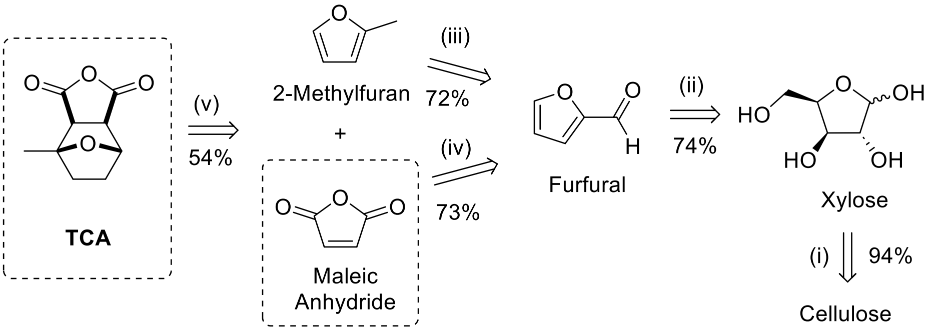

Scheme S5: Potential renewable synthesis of (4*R*,7*S*)-4-methylhexahydro-4,7-epoxyisobenzofuran-1,3-dione (TCA) and maleic anhydride (MA). (i) Mäki-Arvela *et al.*[32] (ii) Gokhale *et al.*[33] (iii) Niu *et al.*[52] (iv) Alonso-Fagúndez *et al.*[35] (v) Zhang *et al.*[9]

3.6 Butylene Oxide

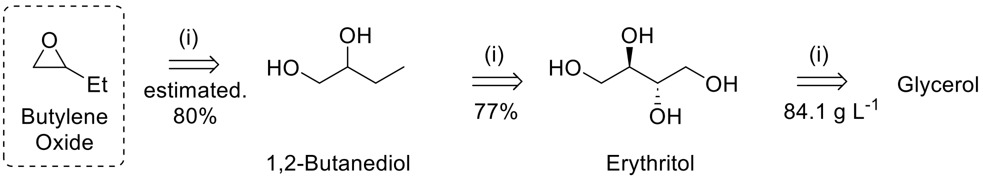

Scheme S6: Potential renewable synthesis of butylene oxide (BO). (i) Tomaszewska *et al.*[12] (ii) Nakagawa *et al.*[11] (iii) Chowdhury *et al* – based on the related synthesis of 1,2-epoxyhexane from 1,2-hexanediol.[10]

3.7 1,2-Epoxydodecane

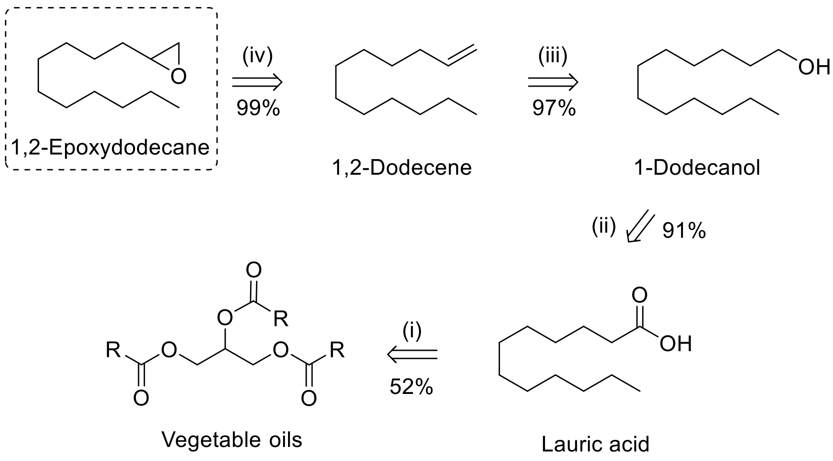

Scheme S7: Potential renewable synthesis of 1,2-epoxydodecane (EDD). (i) Ng *et al.*[13] (ii) Suseela *et al.*[15] (iii) Walker *et al.*[16] (iv) Villo *et al.*[14]

3.8 1,2-Epoxy-9-dodecene

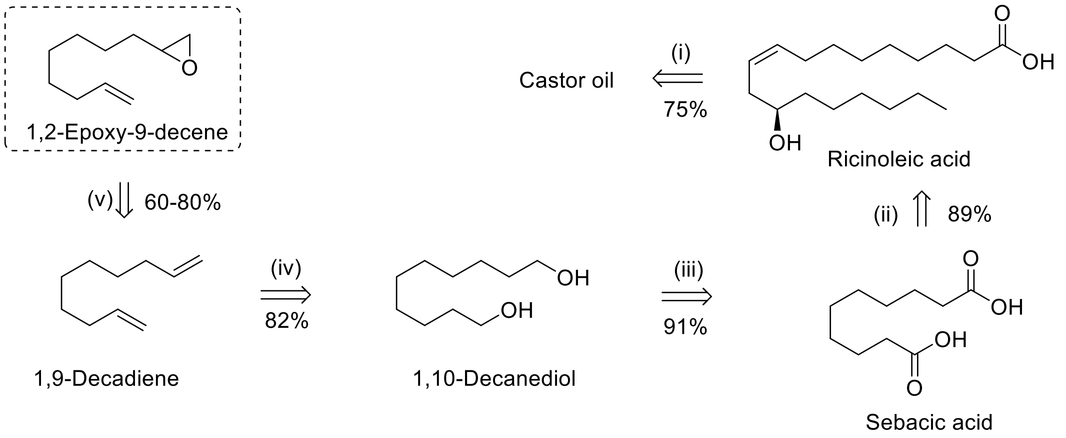

Scheme S8: Potential renewable synthesis of 1,2-epoxydecene (ED). (i) Borsotti *et al.*[20] (ii) Atapalkar *et al.*[17] (iii) Hojabri *et al.*[19] (iv) Xinzhi *et al.*[18] (v) Alt *et al.*[21]

3.9 Epichlorohydrin

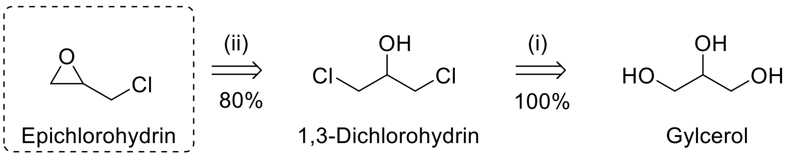

Scheme S9: Potential renewable synthesis of epichlorohydrin (ECH). (i) Tesser *et al.*[53] (ii) Lari *et al.*[22]

3.10 Allyl Glycidyl Ether

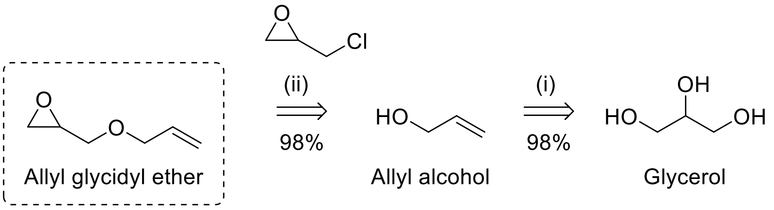

Scheme S10: Potential renewable synthesis of allyl glycidyl ether (AGE). (i) Li *et al.*[24] (ii) Hau *et al.*[54]

3.11 2-Ethylhexyl Glycidyl Ether

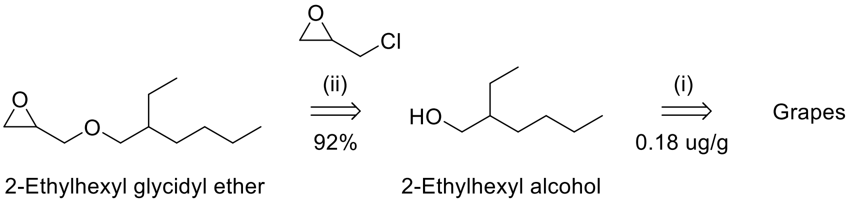

Scheme S11: Potential renewable synthesis of 2-ethylhexyl glycidyl ether (EHGE). (i) Deng *et al.*[25] (ii) Duk *et al.*[55]

3.12 Benzyl Glycidyl Ether

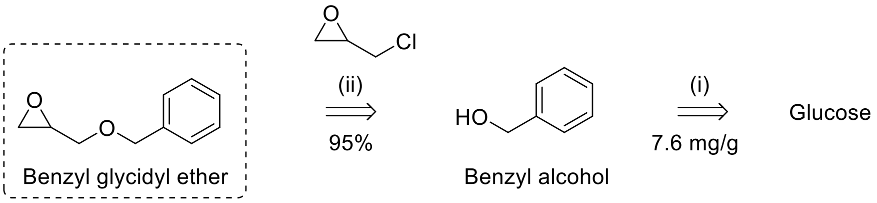

Scheme S12: Potential renewable synthesis of 2-ethylhexyl glycidyl ether (EHGE). (i) Pugh *et al.*[26] (ii) Miyano *et al.*[56]

3.13 2,5-Dihydrofuran

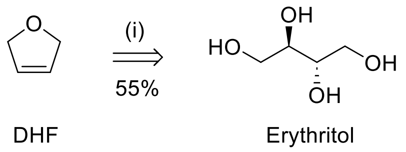

Scheme S13: Potential renewable synthesis of 2,5-dihydrofuran (DHF). (i) Arceo *et al.*[27]

3.14 7-Oxabicyclol-[2.2.1] heptane

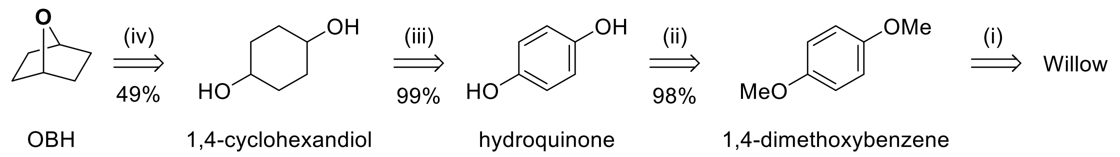

Scheme S14: Potential renewable synthesis of 7-oxabicyclol-[2.2.1] heptane (OBH). (i) Dötterl *et al.*[28] (ii) Bomon *et al.*[29] (iii) Shi *et al.*[30] (iv) Fehnel *et al.*[31]

4.0 Characterization Data for Polymers Described in Table 1

4.1 ^{1}H NMR Spectra for Polymers Described in Table 1

4.1.1 NMR Spectra for P1

P1 - P(MA,BO,BO)

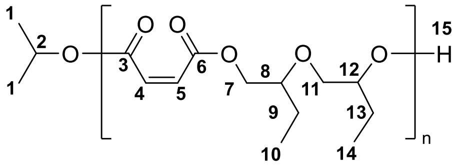

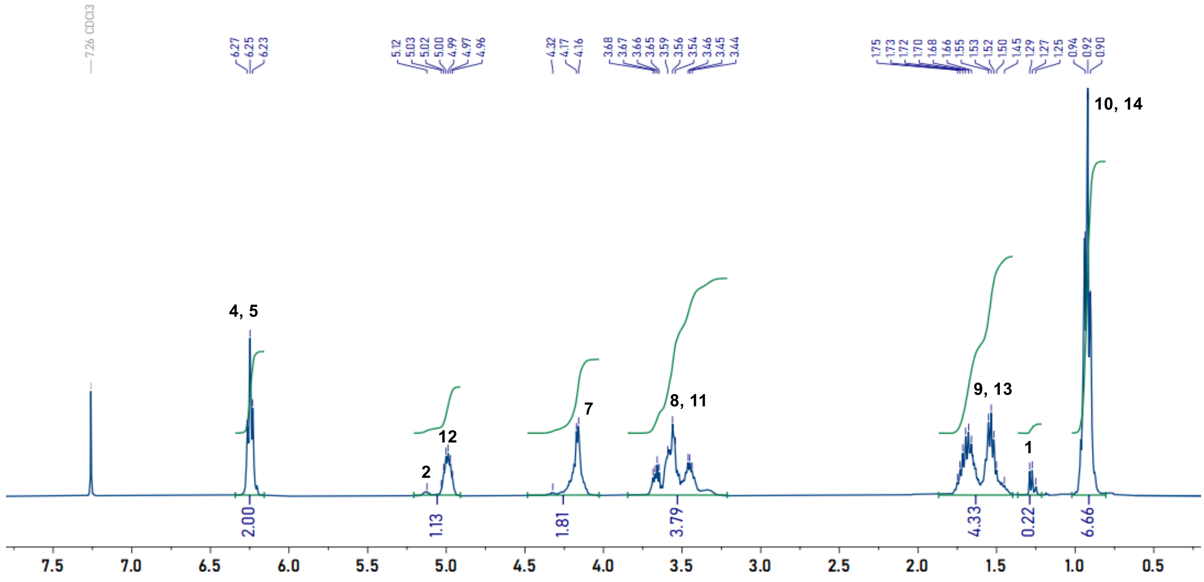

Figure S1: ^{1}H NMR spectrum from the reaction of [Cat]:[MA]:[BO] = 1: 50: 1150, (work up in MeOH/CH_2Cl_2). Spectrum corresponds to Table 1, **P1** (400 MHz, $CDCl_3$). $M_{n,NMR}$ = 7.2 kg mol^{-1}.

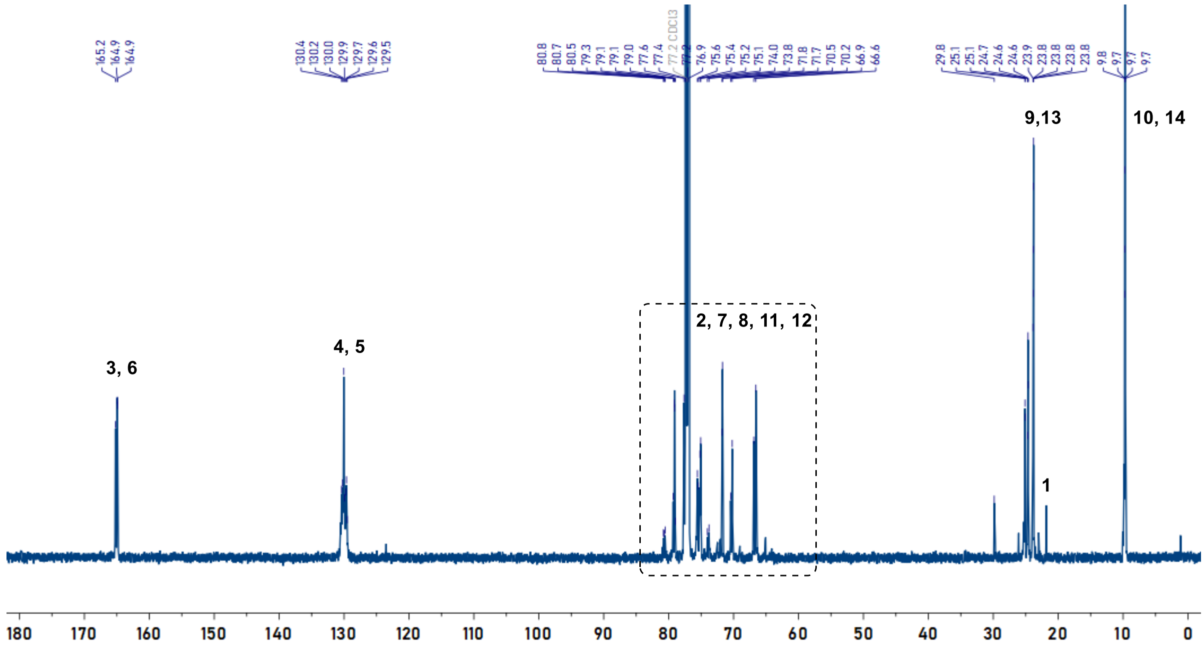

Figure S2: ^{13}C{^{1}H} NMR spectrum from the reaction of [Cat]:[MA]:[BO] = 1: 50: 1150. Spectrum corresponds to Table 1, **P1** (151 MHz, $CDCl_3$).

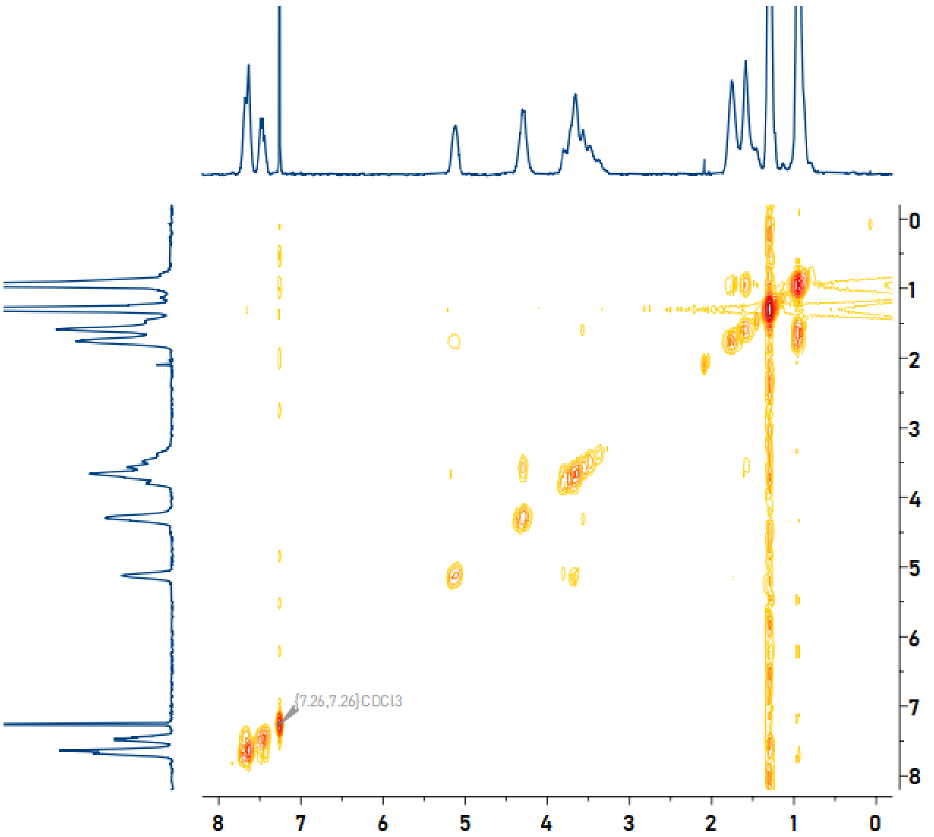

Figure S3: ^{1}H COSY NMR spectrum from the reaction of [**1**]:[MA]:[BO] = 1: 50: 1150, from Table 1, **P1** (400 MHz, $CDCl_3$).

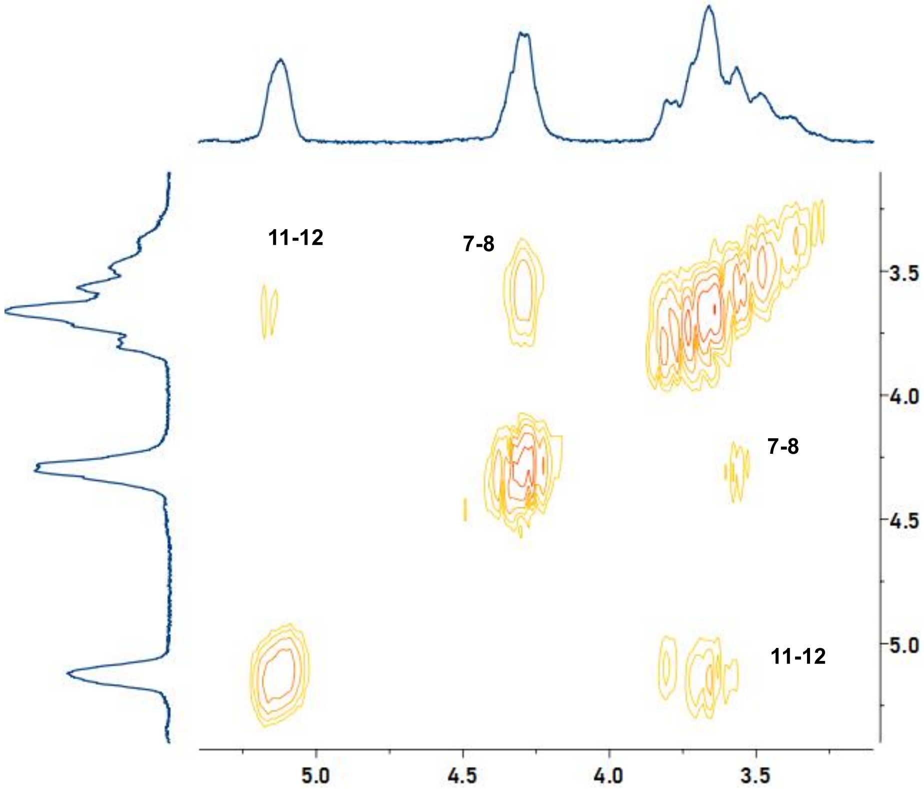

Figure S4: Magnified ^{1}H COSY NMR spectrum from the reaction of [**1**]:[MA]:[BO] = 1: 50: 1150, from Table 1, **P1** (400 MHz, $CDCl_3$).

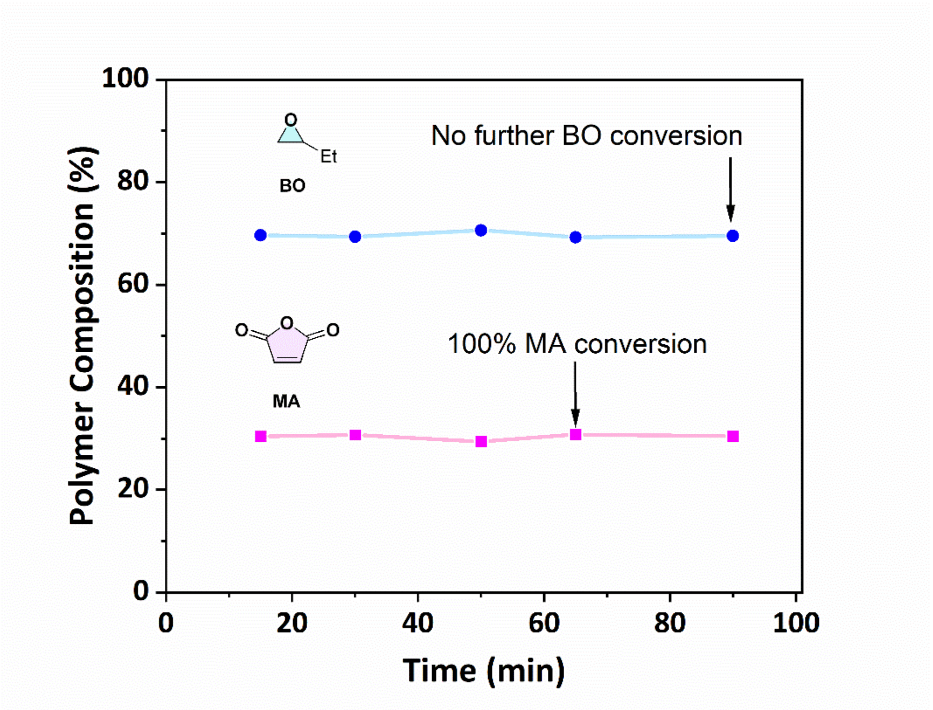

Figure S5: Plot of polymer composition vs time from aliquots of the reaction of [**1**]:[MA]:[BO] = 1: 50: 1150, from Table 1, **P1** (400 MHz, $CDCl_3$). Plot confirms that the anhydride/epoxide selectivity of the reaction is maintained throughout and after the complete consumption of MA no further BO is polymerized.

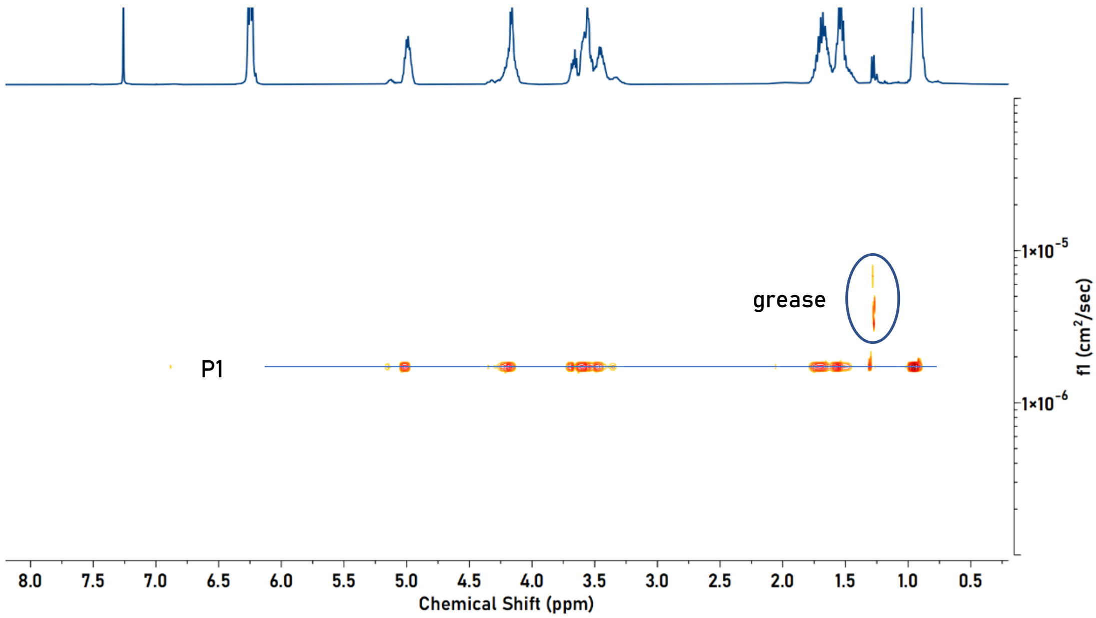

Figure S6: DOSY NMR spectrum from the reaction of [**1**]:[MA]:[BO] = 1: 50: 1150, from Table 1, **P1** (500 MHz, $CDCl_3$).

4.1.1.1 End-group Analysis of P1

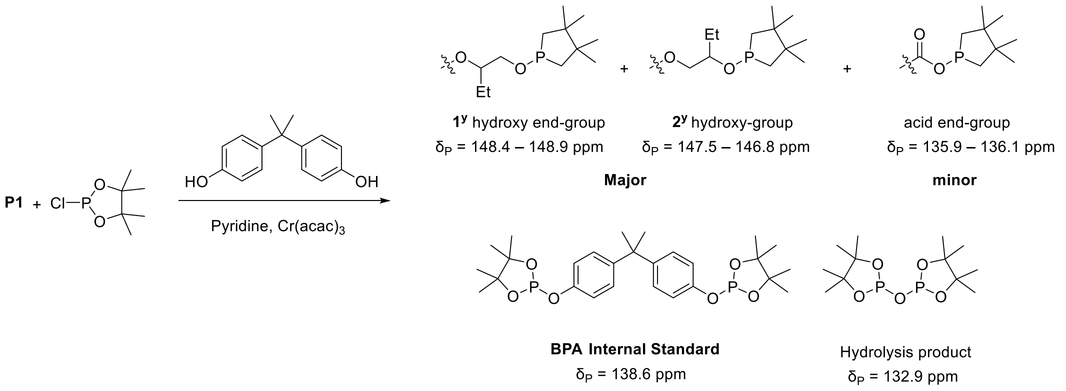

Scheme S15: End group analysis was performed according to literature.[57] **P1** (20 mg) was dissolved in $CDCl_3$ (0.5 mL) and a solution (40 µL) containing $Cr(acac)_3$ (5.5 mg) and internal standard, bisphenol A (400 mg) in pyridine (10 mL) was added followed by 40 µL of tetramethylethylene chlorophosphite. The polymer was then analysed by $^{31}P\{^1H\}$ NMR.

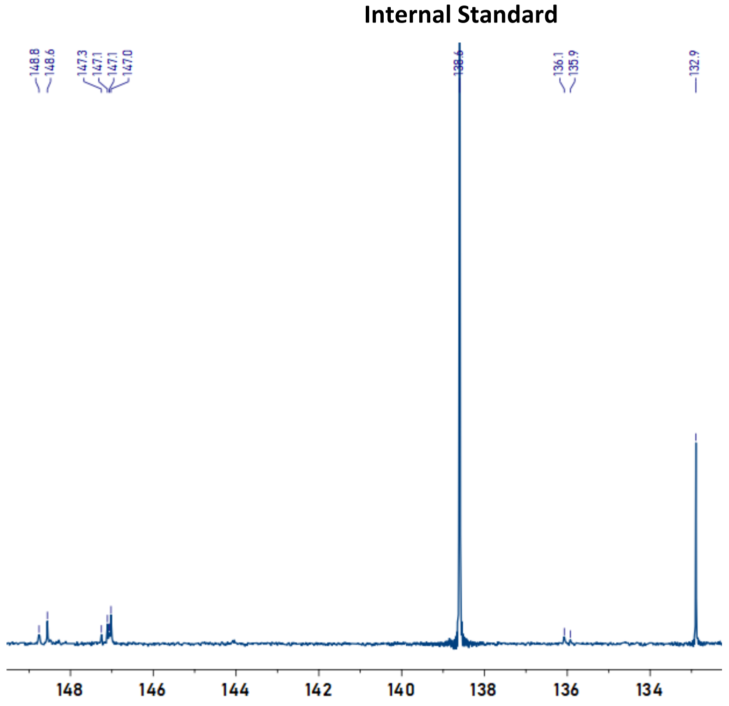

Figure S7: $^{31}P\{^1H\}$ NMR spectrum from the reaction **P1** with tetramethylethylene chlorophosphite (162 MHz, $CDCl_3$). The major polymer resonances at 148.1-148.9 and 147.5-146.8 ppm are characteristic of a mixture of primary and secondary alcohol polymer end-groups, respectively (>90%).[58] The minor polymer resonances at 135.9-136.1 ppm are characteristic of carboxylic acid end groups (<10%). The standard at 138.6 ppm is Bisphenol-A and the resonance at 132.9 ppm is the hydrolysis product of the chlorophosphite reagent.

4.1.2 NMR Spectra for P2

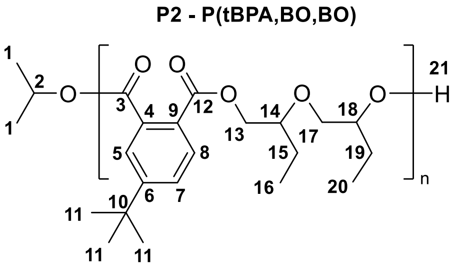

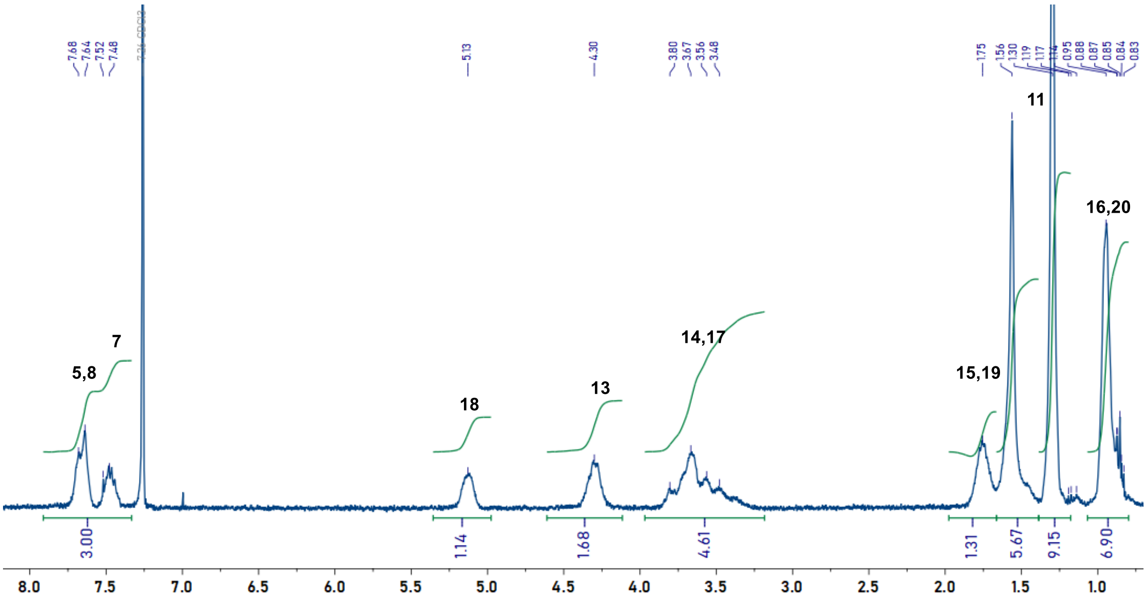

Figure S8: ^{1}H NMR spectrum from the reaction of [Cat]:[tBPA]:[BO] = 1: 50: 1150 (work-up in MeOH/CH_2Cl_2). Spectrum corresponds to Table 1, **P2** (400 MHz, $CDCl_3$).

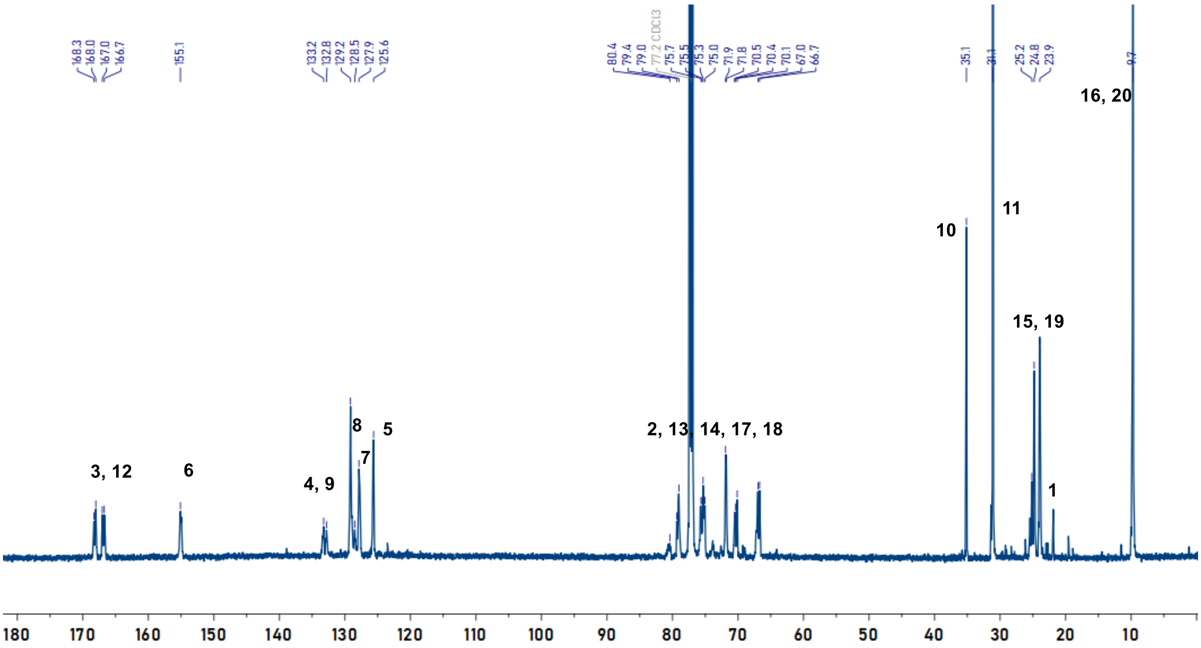

Figure S9: ^{13}C{^{1}H} NMR spectrum from the reaction of [Cat]:[MA]:[BO] = 1: 50: 1150, Table 1, **P2** (151 MHz, $CDCl_3$).

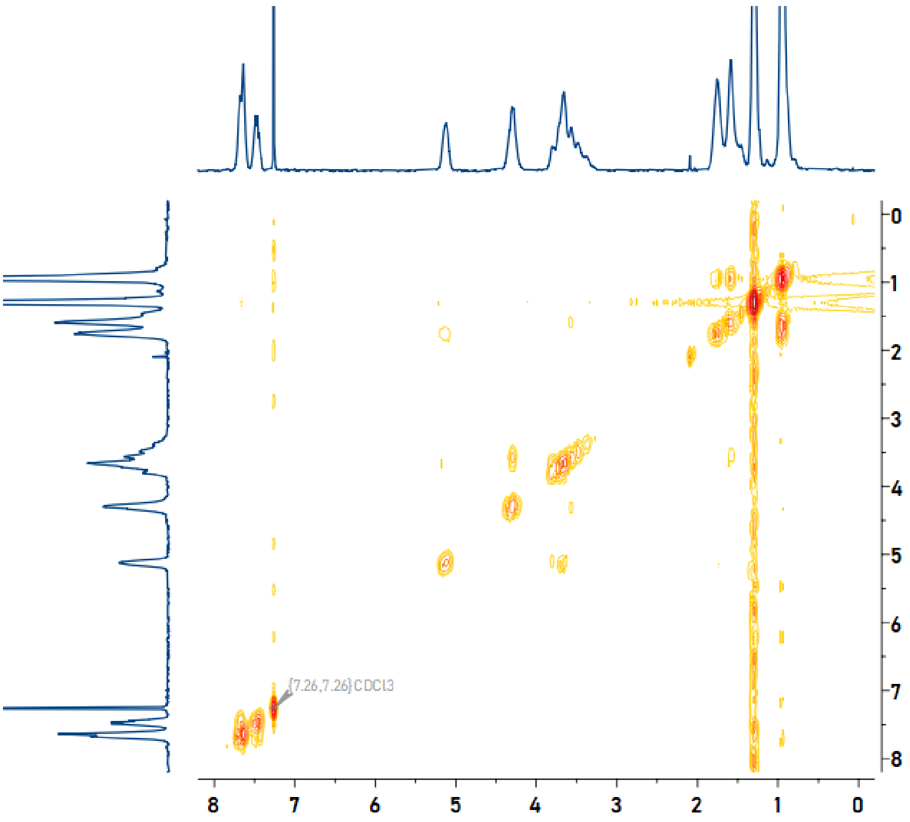

Figure S10: ^{1}H COSY NMR spectrum from the reaction of [**1**]:[tBPA]:[BO] = 1: 50: 1150, Table 1, **P2** (400 MHz, $CDCl_3$).

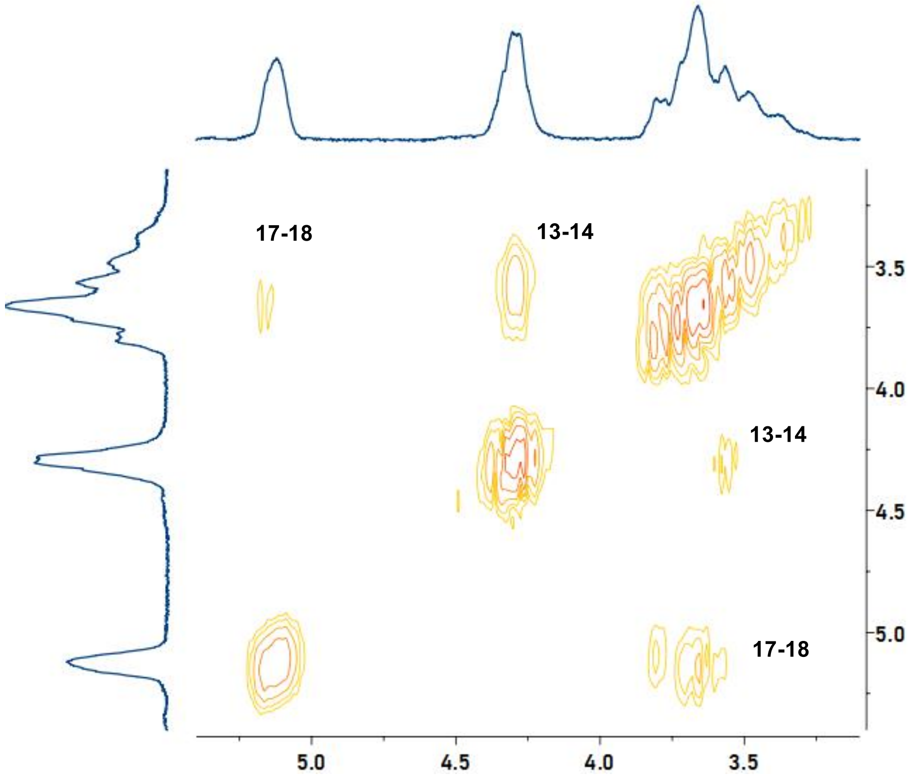

Figure S11: Magnified ^{1}H COSY NMR spectrum from the reaction of [**1**]:[tBPA]:[BO] = 1: 50: 1150, Table 1, **P2** (400 MHz, $CDCl_3$).

4.1.3 NMR Spectra for P3

P3 - P(DPA,BO,BO)

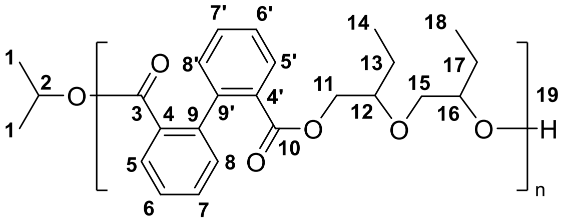

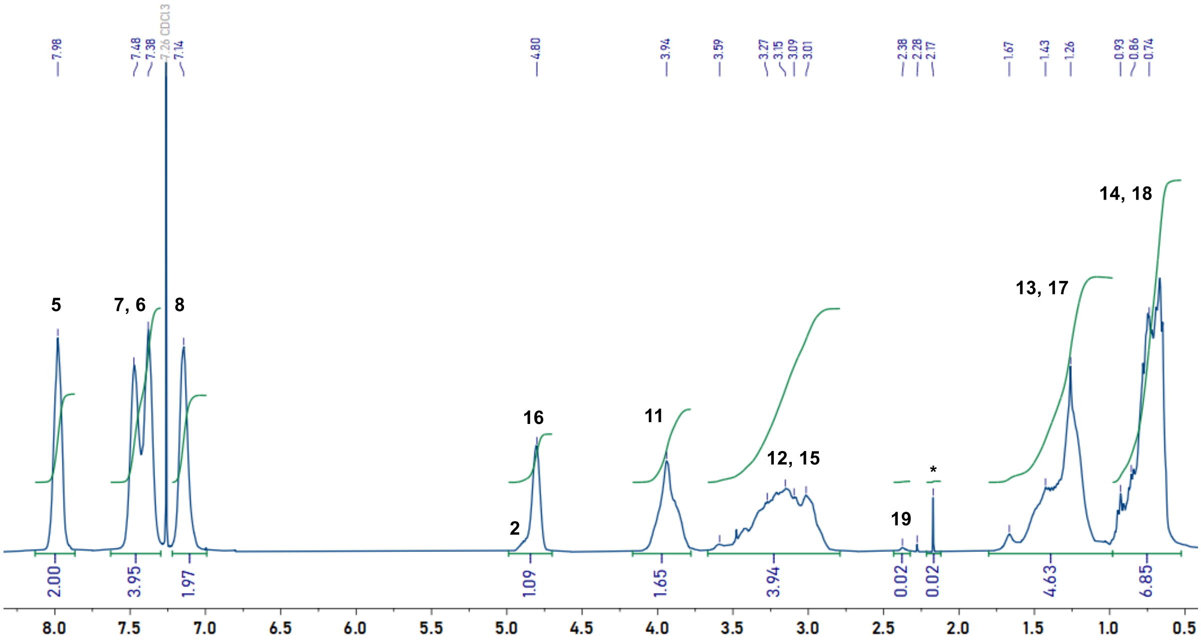

Figure S12: ^{1}H NMR spectrum from the reaction of [Cat]:[DPA]:[BO] = 1: 50: 1150, (work-up in MeOH/ CH_2Cl_2). Spectrum corresponds to Table 1, **P3** (400 MHz, $CDCl_3$). *Acetone

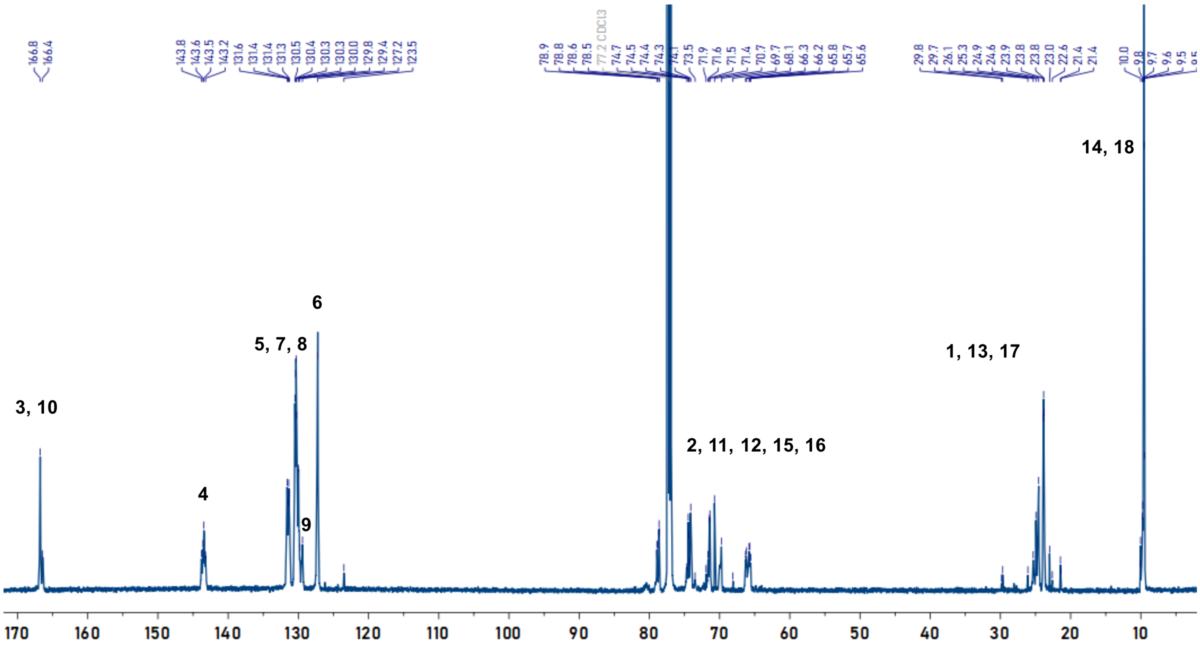

Figure S13: $^{13}C\{^{1}H\}$ NMR spectrum from the reaction of [Cat]:[DPA]:[BO] = 1: 50: 1150, Table 1, **P3** (151 MHz, $CDCl_3$).

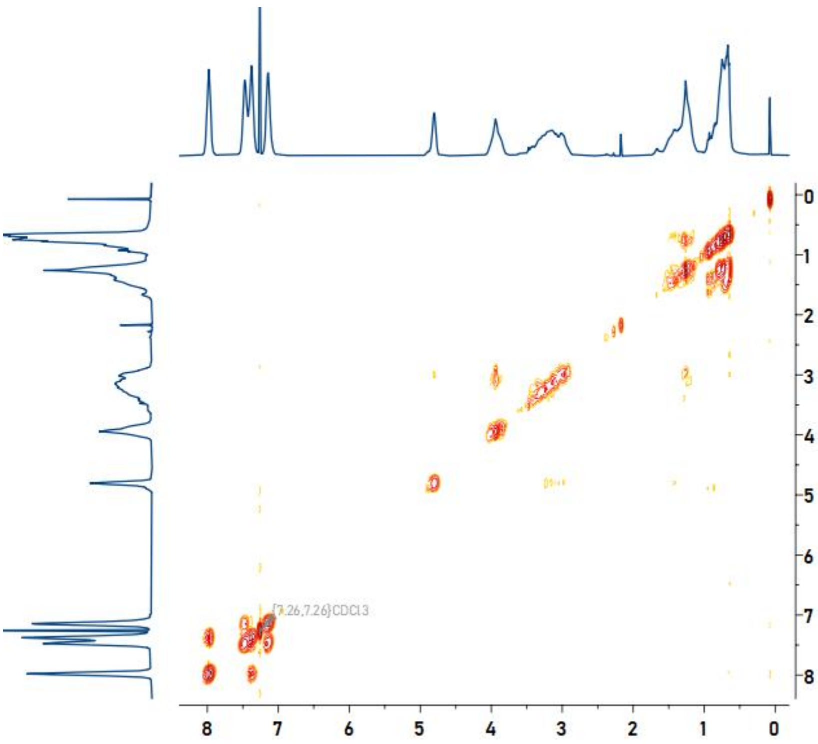

Figure S14: ^{1}H COSY NMR spectrum from the reaction of [**1**]:[DPA]:[BO] = 1: 50: 1150, Table 1, **P3** (400 MHz, $CDCl_3$).

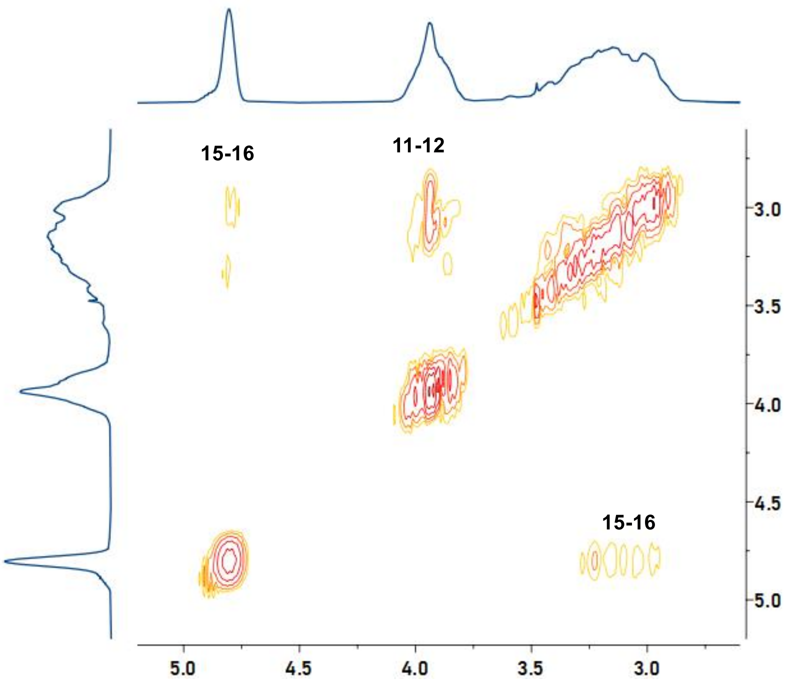

Figure S15: Magnified ^{1}H COSY NMR spectrum from the reaction of [**1**]:[DPA]:[BO] = 1: 50: 1150, Table 1, **P3** (400 MHz, $CDCl_3$).

4.1.4 NMR Spectra for P4

P4 - P(DGA,BO,BO)

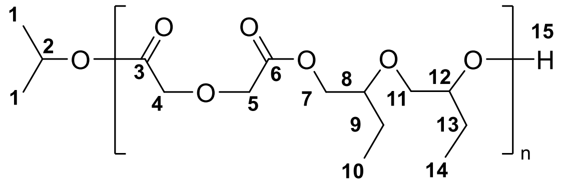

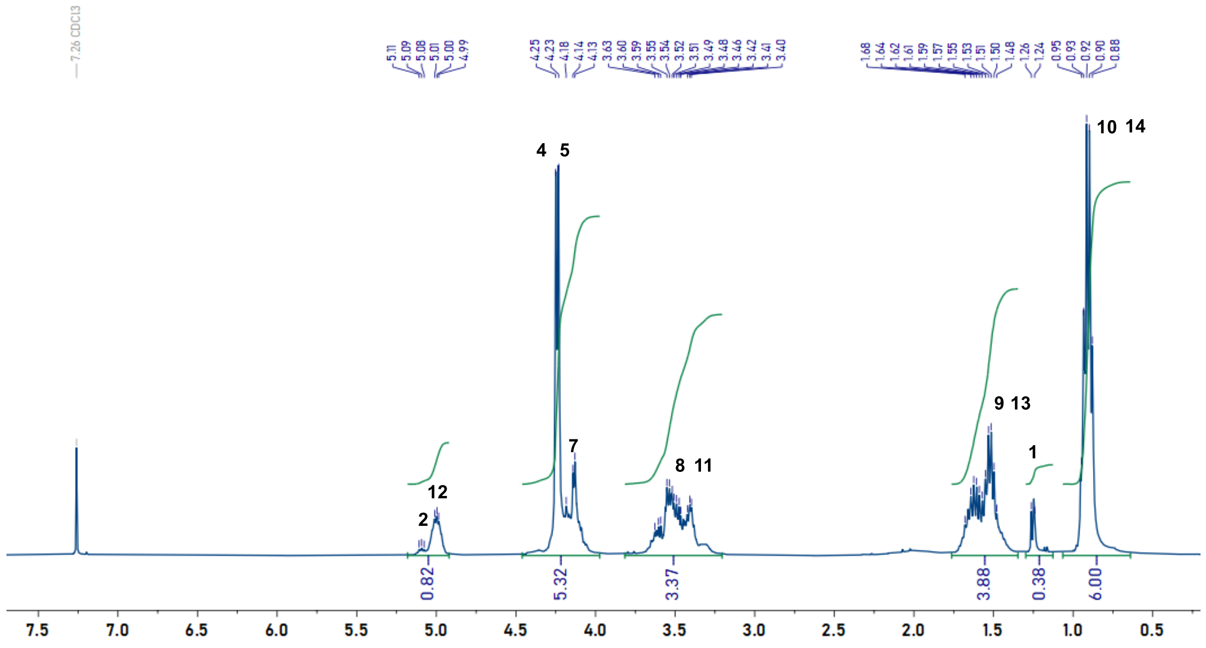

Figure S16: ^{1}H NMR spectrum from the reaction of [Cat]:[DGA]:[BO] = 1: 50: 1150, (work-up in MeOH/Pentane). Spectrum corresponds to Table 1, **P4** (400 MHz, $CDCl_3$). $M_{n,NMR}$ = 3.7 kg mol^{-1}.

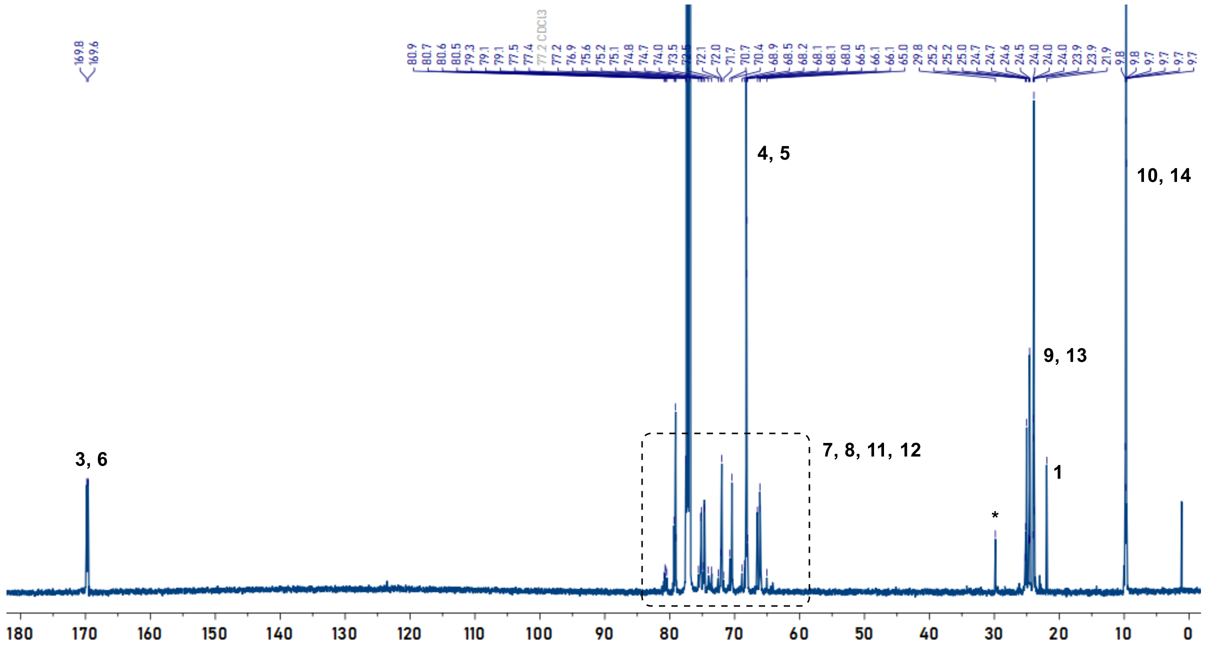

Figure S17: $^{13}C\{^{1}H\}$ NMR spectrum from the reaction of [Cat]:[DGA]:[BO] = 1: 50: 1150, Table 1, **P4** (151 MHz, $CDCl_3$). * = 'H' - grease

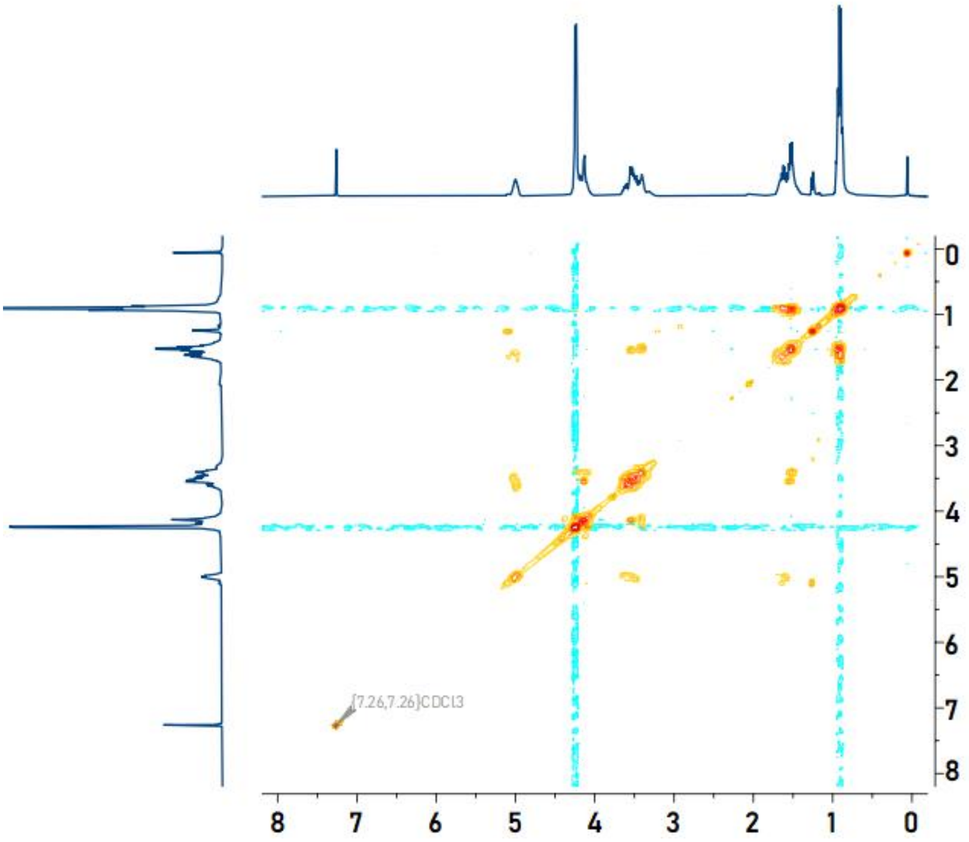

Figure S18: ^{1}H COSY NMR spectrum from the reaction of [**1**]:[DGA]:[BO] = 1: 50: 1150, Table 1, **P4** (400 MHz, $CDCl_3$).

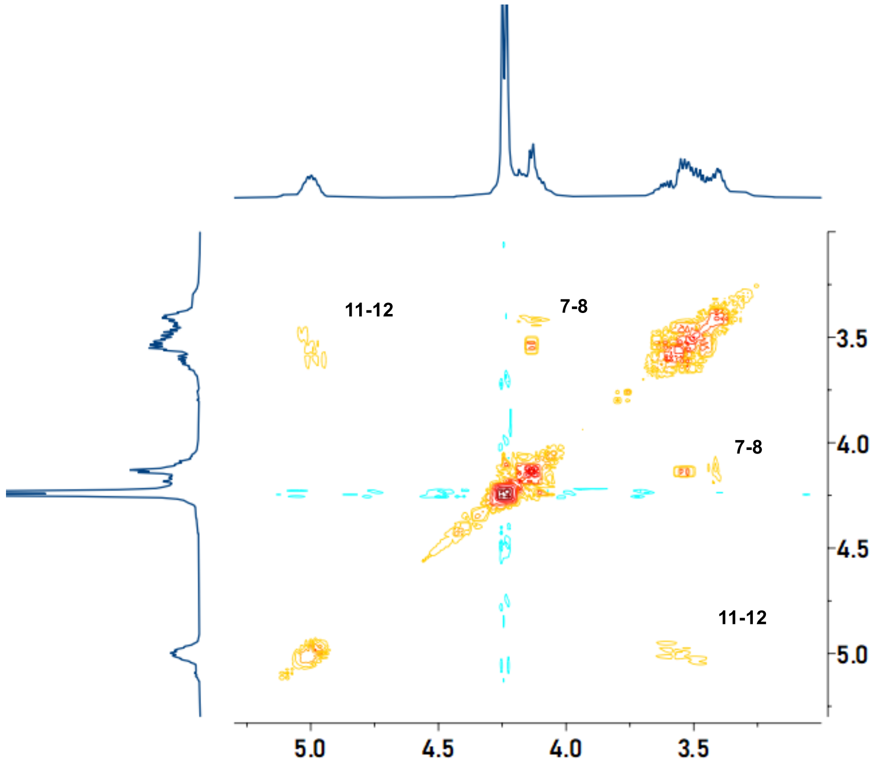

Figure S19: Magnified ^{1}H COSY NMR spectrum from the reaction of [**1**]:[DGA]:[BO] = 1: 50: 1150, Table 1, **P4** (400 MHz, $CDCl_3$).

4.1.5 NMR Spectra for P5

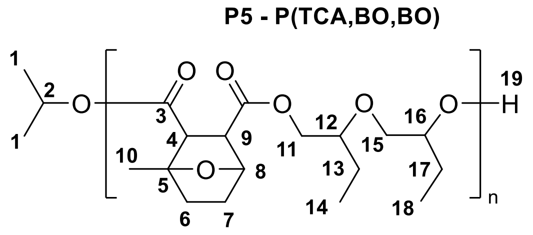

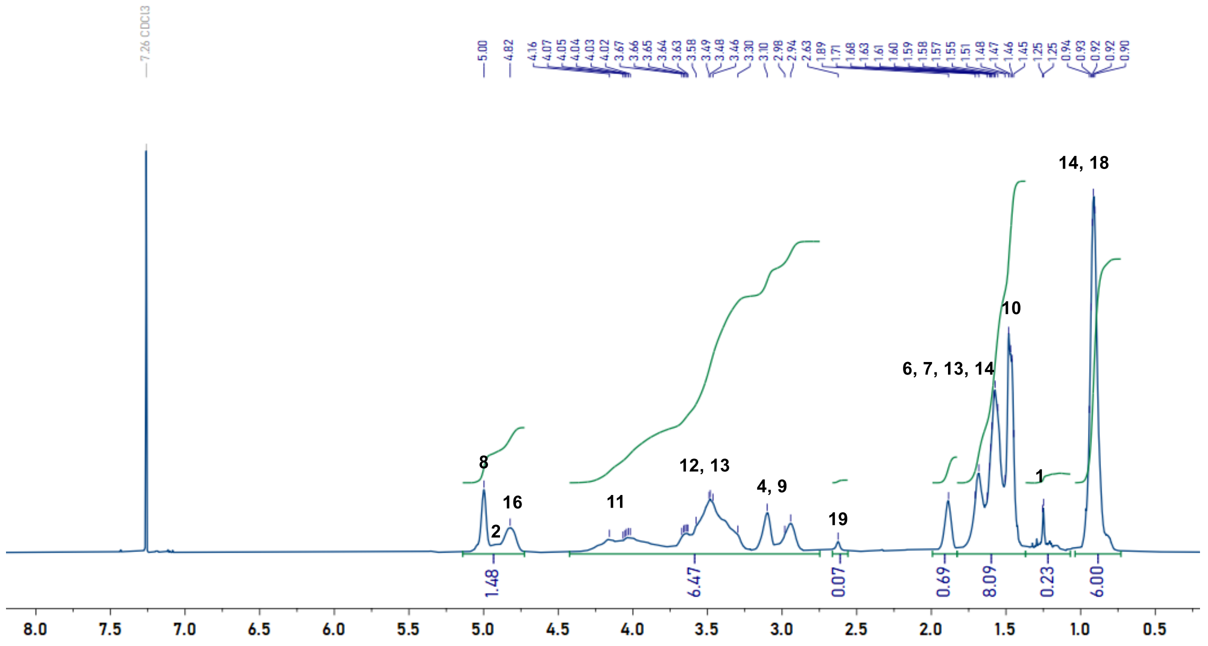

Figure S20: ^{1}H NMR spectrum from the reaction of [Cat]:[TCA]:[BO] = 1: 50: 1150, (work-up in MeOH/pentane). Spectrum corresponds to Table 1, **P5** (400 MHz, $CDCl_3$). $M_{n,NMR}$ = 7.8 kg mol^{-1}.

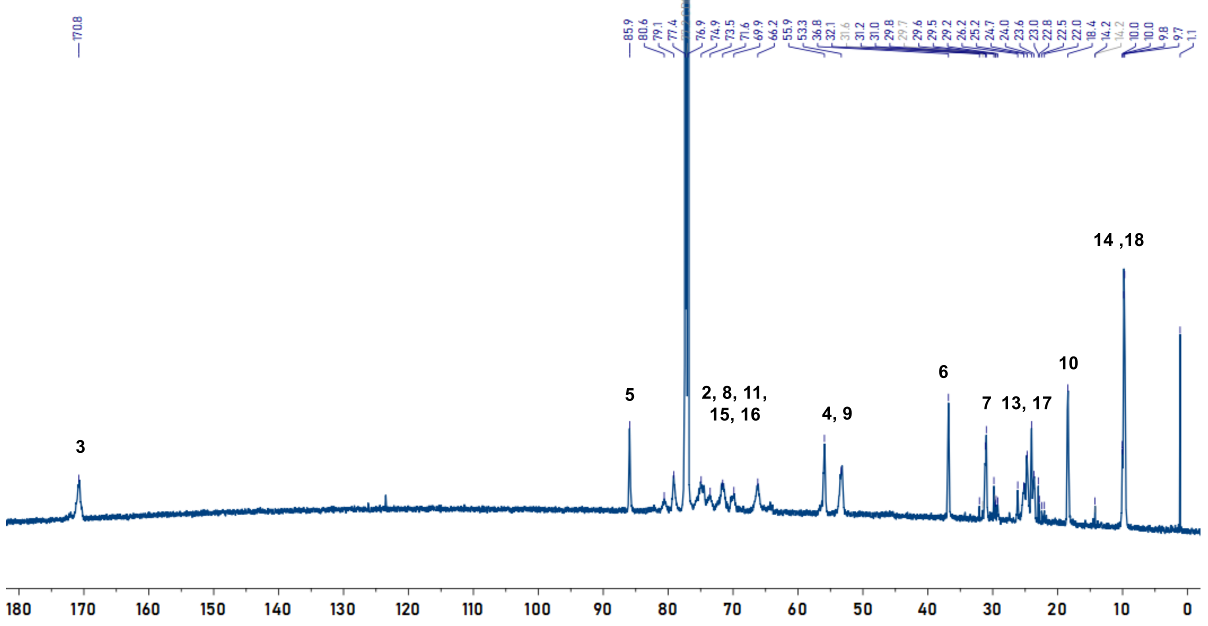

Figure S21: $^{13}C\{^{1}H\}$ NMR spectrum from the reaction of [Cat]:[TCA]:[BO] = 1: 50: 1150, Table 1, **P5** (151 MHz, $CDCl_3$).

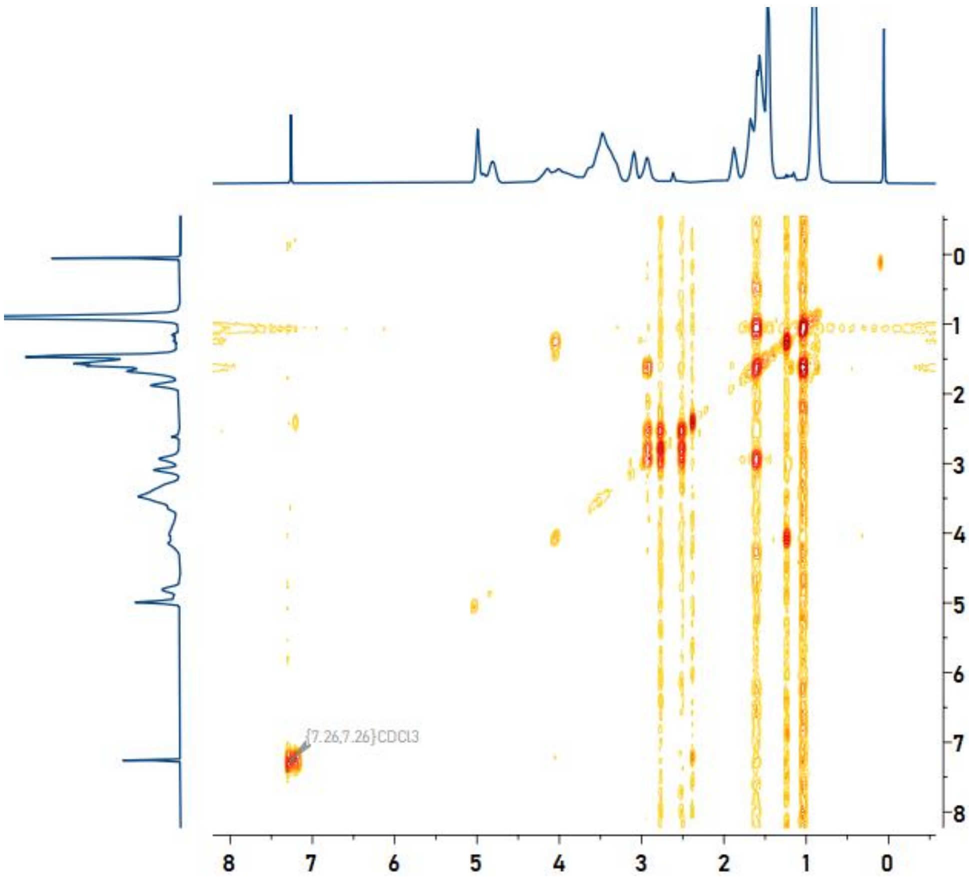

Figure S22: ^{1}H COSY NMR spectrum from the reaction of [Cat]:[TCA]:[BO] = 1: 50: 1150, Table 1, **P5** (400 MHz, $CDCl_3$).

Note, no correlations between protons 11-12, and 15-16 were observed in the ^{1}H COSY NMR spectrum. Reassuringly, in accordance with our structure determination, no correlations were observed relating to a perfectly alternating polymer, ie, P(TCA-*alt*-BO). Therefore, this structure is proposed by analogy to the other polymers **P1-16**.

4.1.6 NMR Spectra for P6

P(PA,BO,BO)

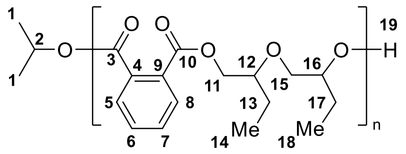

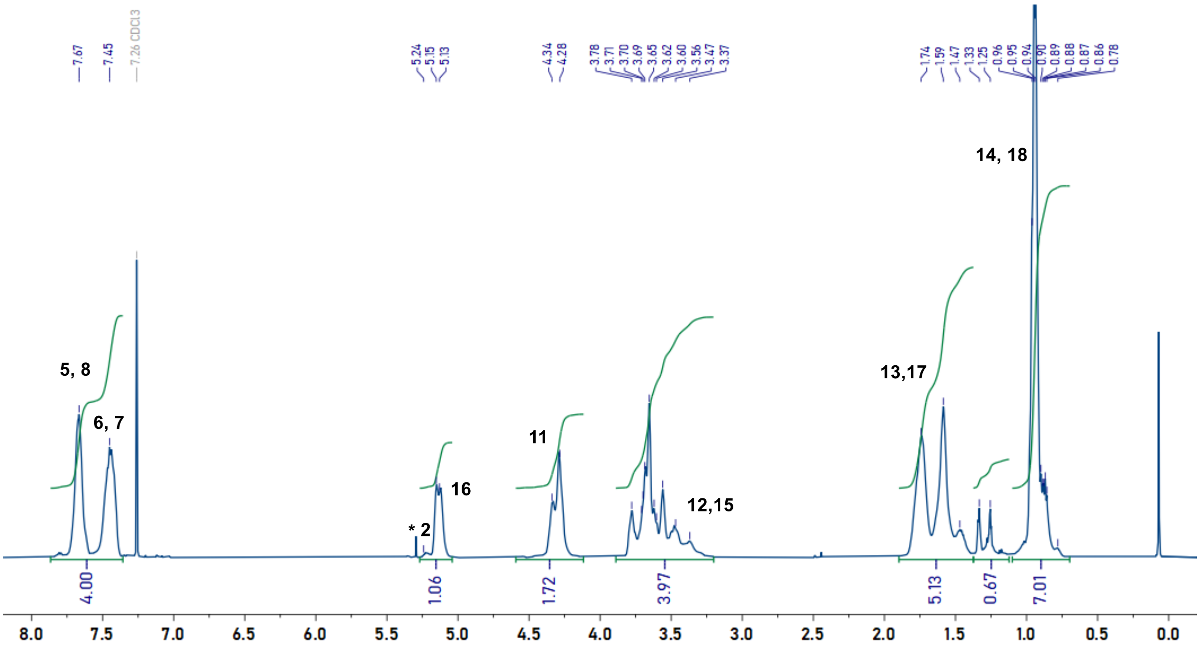

Figure S23: ^{1}H NMR spectrum from the reaction of [Cat]:[PA]:[BO] = 1: 50: 1150, (work-up in MeOH/CH_2Cl_2). Spectrum corresponds to Table 1, **P6** (400 MHz, $CDCl_3$). * = CH_2Cl_2.

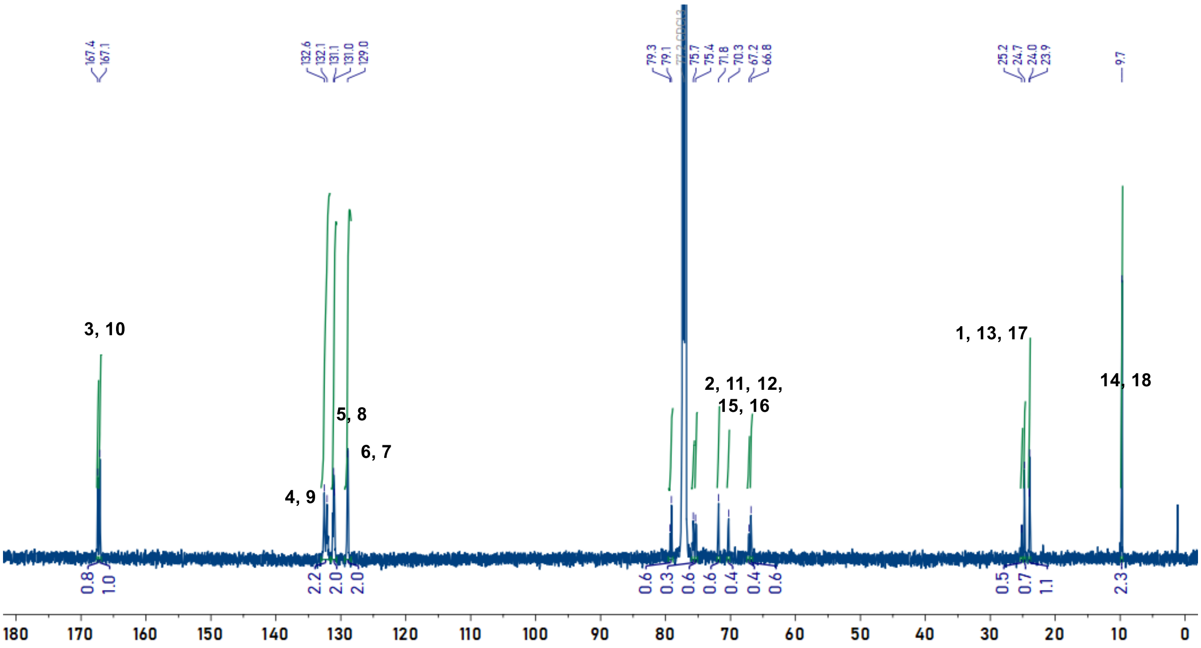

Figure S24: Quantitative $^{13}C\{^{1}H\}$ NMR spectrum from the reaction of [Cat]:[PA]:[BO] = 1: 50: 1150, Table 1, **P6** (151 MHz, $CDCl_3$).

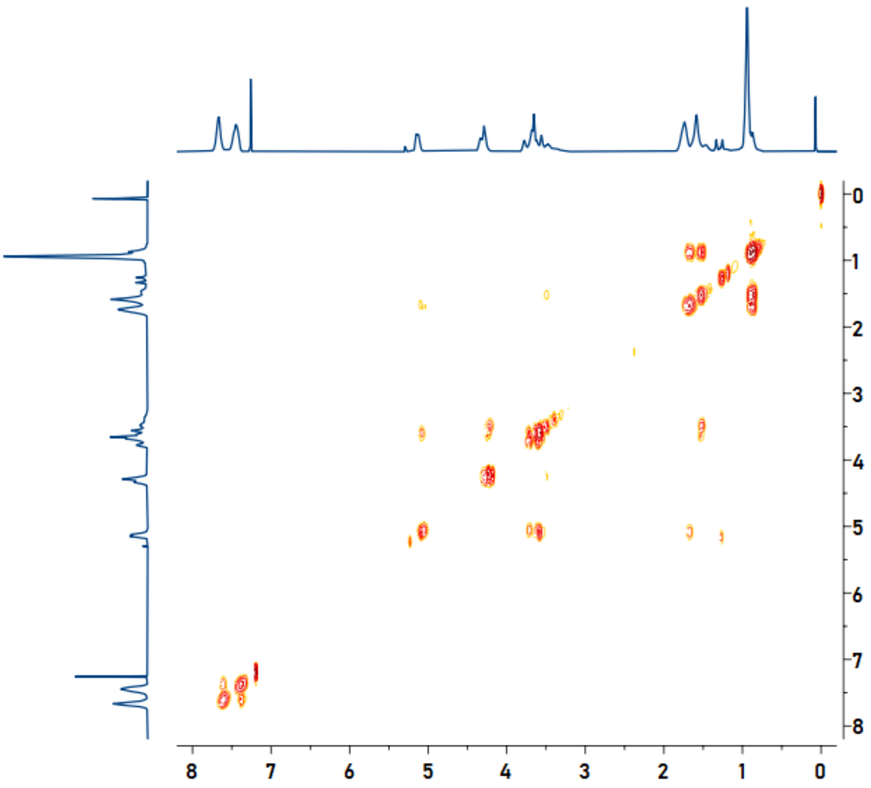

Figure S25: ^{1}H COSY NMR spectrum from the reaction of [Cat]:[PA]:[BO] = 1: 50: 1150, Table 1, **P6** (400 MHz, $CDCl_3$).

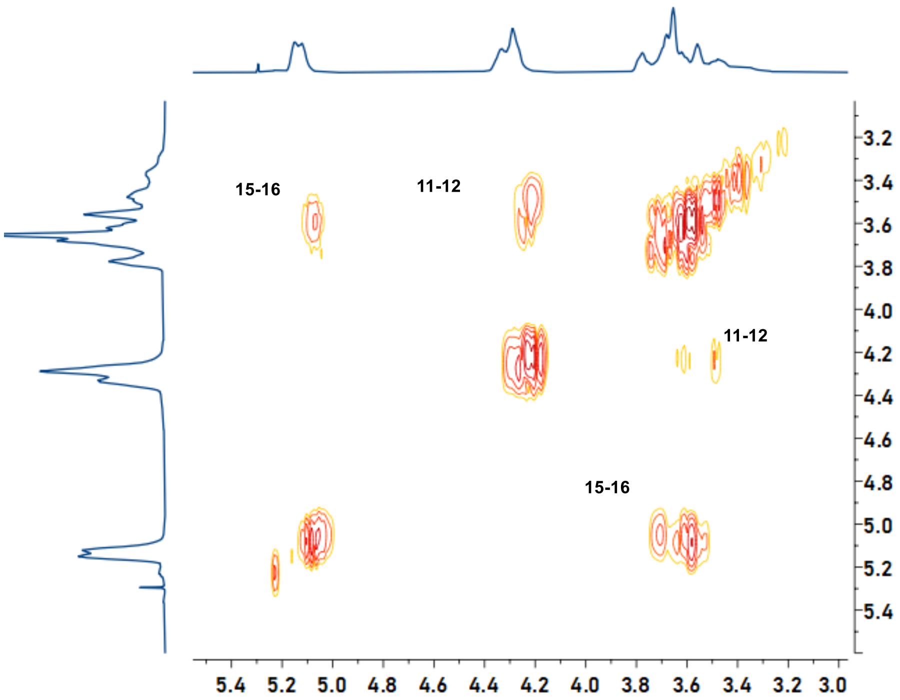

Figure S26: Magnified ^{1}H COSY NMR spectrum from the reaction of [Cat]:[PA]:[BO] = 1: 50: 1150, Table 1, **P6** (400 MHz, $CDCl_3$).

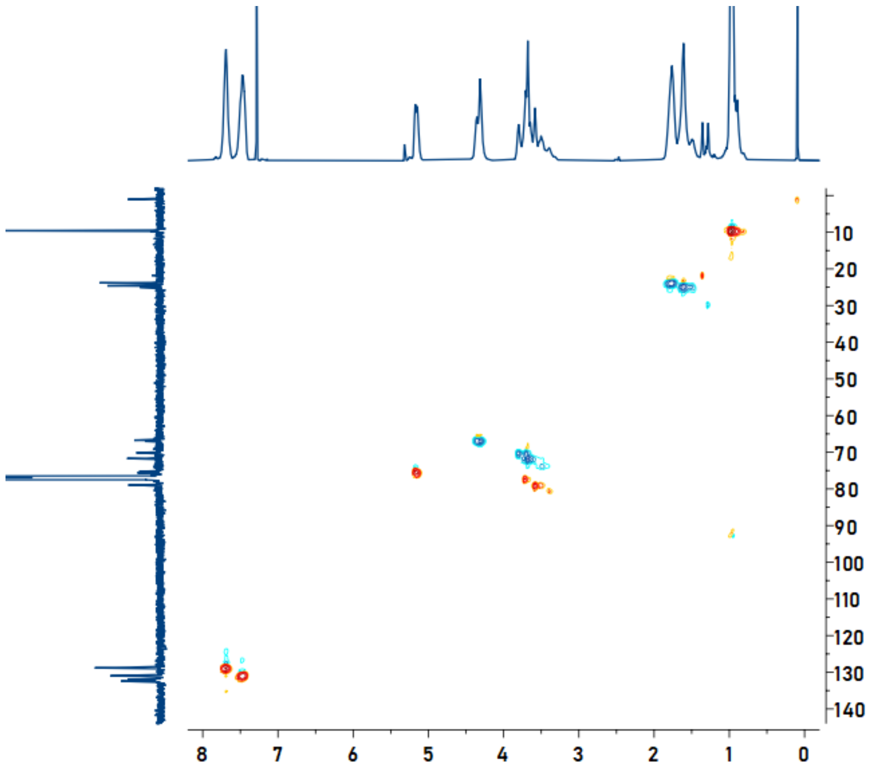

Figure S27: (^{1}H,^{13}C)-HSQC NMR spectrum from the reaction of **P6**, from Table 1.

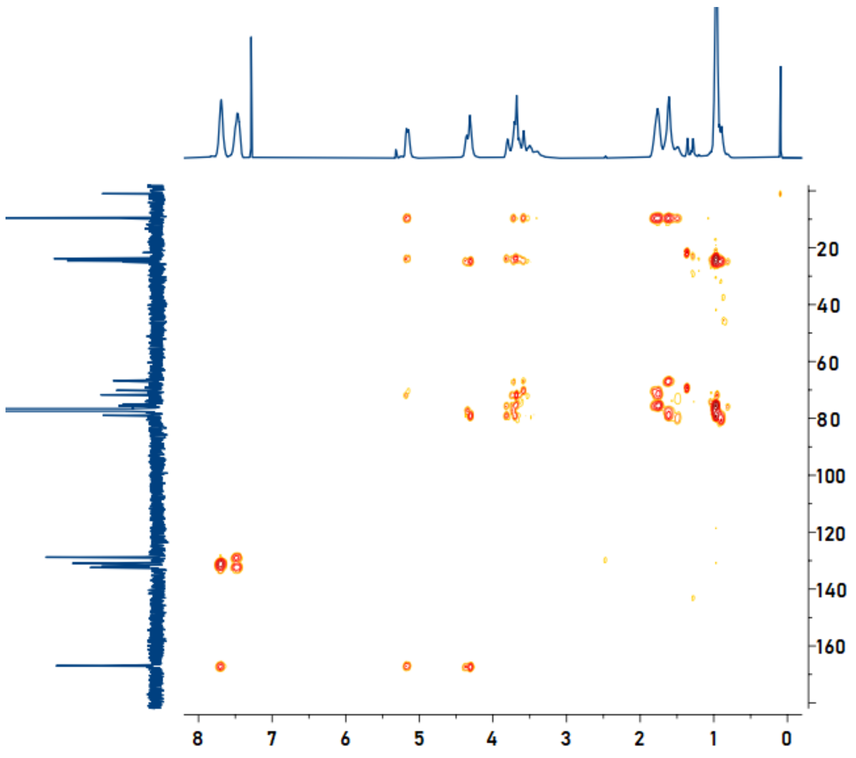

Figure S28: (^{1}H,^{13}C)-HMBC NMR spectrum from the reaction of **P6**, from Table 1.

4.1.6.1 Regioselectivity of P6

To determine the regioselectivity of an **ABB** repeating unit all combinations epoxide (**B**) insertion must be considered, Chart **S1**. Using **P6** as an exemplar, it can be seen that there 12 different combinations of regio-isomers of the **BB** insertion, Chart **S1A**. This also includes diastereomers and enantiomers, these were not observed over all NMR techniques. It must also be considered that irregularities will exist across the chain, chart **S1B**, such as an **AB** linkage and extended ether linkage, **AB$_n$**, where n > 2 and any combination of regioisomer for these must be also be considered.

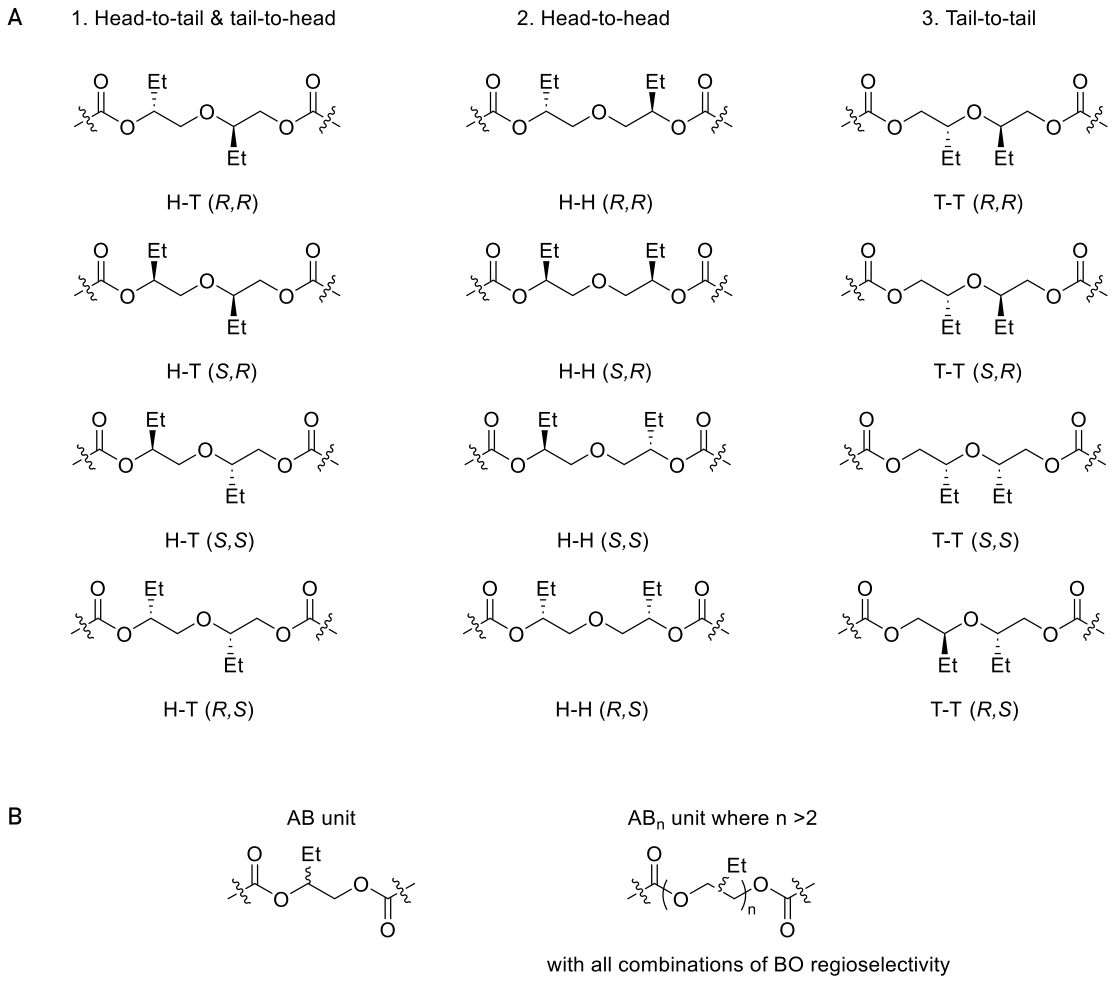

Chart S1: A) All possible combinations of regioisomers, enantiomers and diastereomers of the repeat unit of polymers **P6**-**P14**. B) All possible chain "error" combinations.

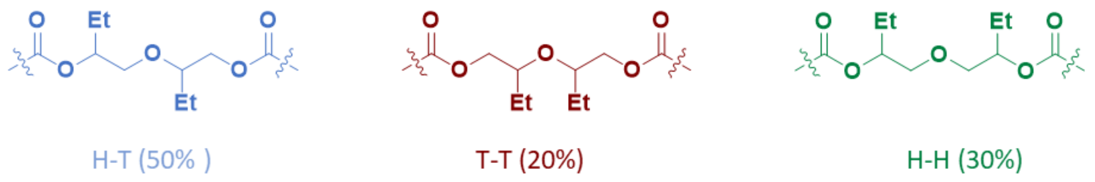

Chart S2: Final regioselectivity of **ABB** repeating unit of **P6** determined by ^{1}H, quantitative ^{13}C, DEPT-135, HSQC and HMBC (Figures S29-34). The regioselectivity suggests insertion step of epoxide is almost random.

Table S1: ^{13}C{^{1}H} NMR assignments to determine polymer regioselectivity of **P6**.

^{13}C NMR Resonance	Dept-135	HSQC	HMBC	Assignment
**Figure S24**	**Figure S32**	**Figure S27**	**Figure S28**	
66.8	-CH$_2$-	4.31-4.36 (1° ester)	3.58 (2° ether) (See resonance 79.2 for full complimentary assignment)	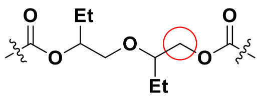 1° ester, H-T
67.2	-CH$_2$-	4.31-4.36 (1° ester)	3.71 (2° ether) (See resonance 77.3 for full complimentary assignment)	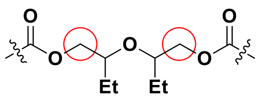 1° ester, T-T
70.3	-CH$_2$-	3.71, 3.80 (1° ethers)	3.58 (1° ether) 5.16 (2° ester)	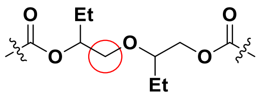 1° ether, H-T
72.8	-CH$_2$-	3.64, 3.68, 3.73 (1° ethers)	3.64, 3.68, 3.73 (1° ethers) 5.16 (2° ester)	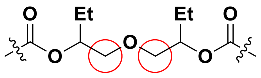 1° ether, H-H
75.4	-CH-	5.16 (2° ester)	3.68, 3.74 (1° ethers)	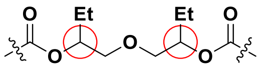 2° ester, H-H
75.7	-CH-	5.16 (2° ester)	3.71 (2° ether) 3.80 (1° ether)	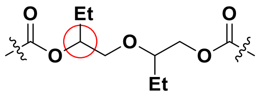 2° ester, H-T
77.3	-CH-	3.72 (2° ether)	3.72 (2° ether) 4.31 (1° ester)	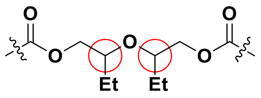 2° ether, T-T
79.2	-CH-	3.58 (2° ether)	3.71, 3.80 (1° ether) 4.31 (1° ester)	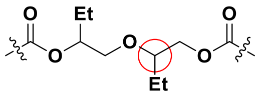 2° ether, H-T

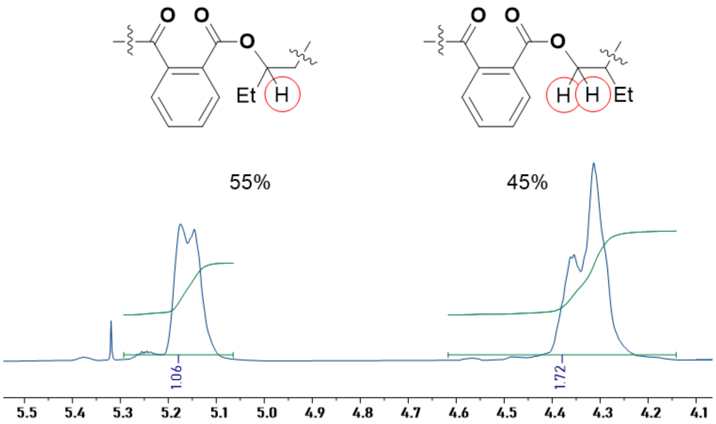

Figure S29: Magnified ^{1}H NMR spectrum from the reaction of **P6**, from Table 1, assigning the relative ratio of ester units adjacent to methine (2°, ~5.1-5.2 ppm) and methylene (1°, ~4.2-4.4 ppm) functionality.

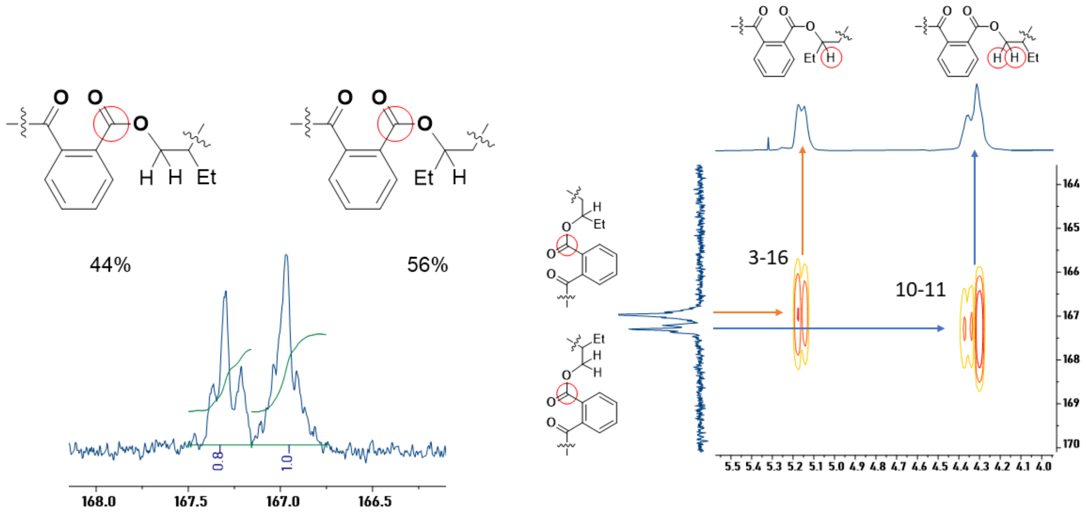

Figure S30: Left: Magnified quantitative ^{13}C NMR spectrum at the carbonyl region of **P6**, from Table 1, assigning the relative ratio of ester units adjacent to methine (2°, ~167.0 ppm) and methylene (1°, 167.3 ppm) functionality. Right: Magnified (^{1}H,^{13}C)-HMBC to assign carbonyl region of **P6**.

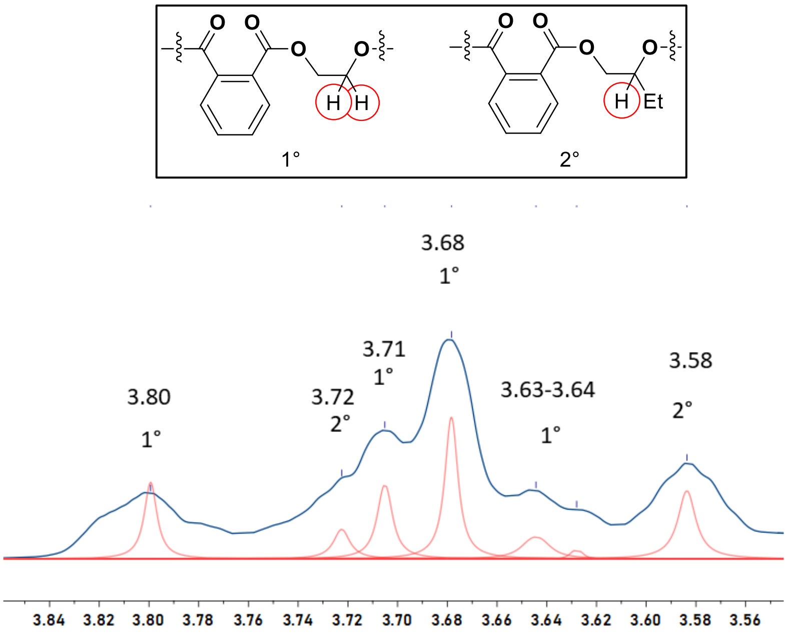

Figure S31: Magnified ^{1}H NMR spectrum from the reaction of **P6**, from Table 1, deconvoluting the ether resonances used to assign **ABB** regioselectivity. Methylene (2°) and methine (1°) ethers were assigned using correlations in (^{1}H,^{13}C)-HSQC.

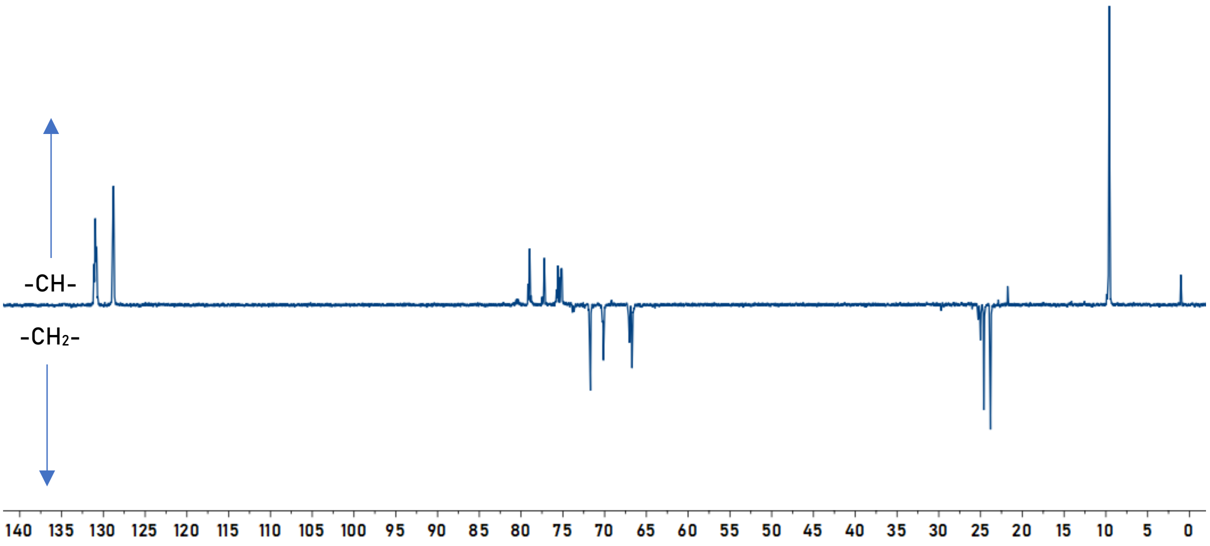

Figure S32: DEPT-135 ^{13}C{^{1}H} NMR spectrum from the reaction of [Cat]:[PA]:[BO] = 1: 50: 1150, Table 1, **P6** (151 MHz, $CDCl_3$).

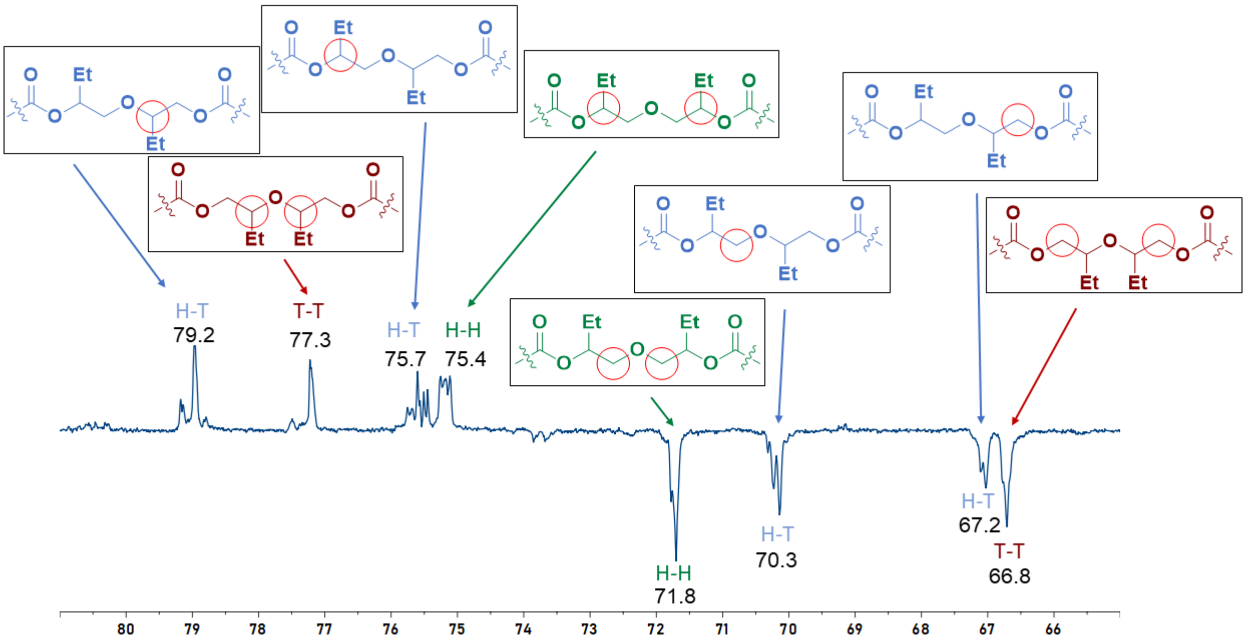

Figure S33: Magnified DEPT-135 $^{13}C\{^1H\}$ NMR spectrum from the reaction of [Cat]:[PA]:[BO] = 1: 50: 1150, Table 1, **P6** (151 MHz, $CDCl_3$). Full assignment of linkages were determined using ^1H, quantitative $^{13}C\{^1H\}$, DEPT-135, HSQC and HMBC.

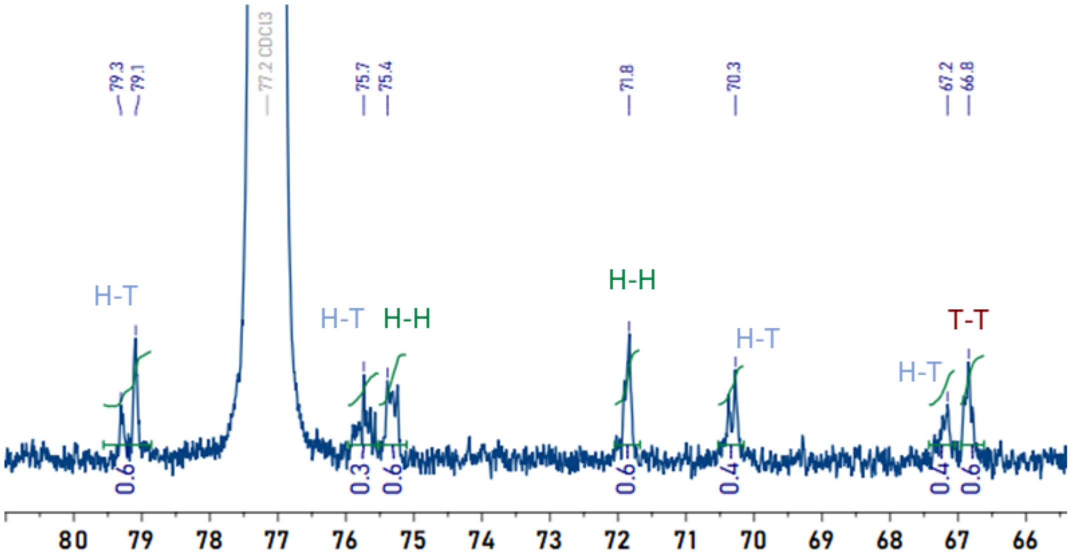

Figure S34: Magnified Quantitative $^{13}C\{^1H\}$ NMR spectrum from the reaction of [Cat]:[PA]:[(*R*)-BO] = 1: 50: 1150, Table 1, **P6** (151 MHz, $CDCl_3$). Full assignment was of linkages were determined using DEPT-135, HSQC and HMBC. Using peaks 71.8, 70.3, 67.2 and 66.8, the final ratio of H-T: H-H: T-T = 50: 30: 20.

4.1.7 NMR Spectra for P7

P(PA,(*R*)-BO,(*R*)-BO)

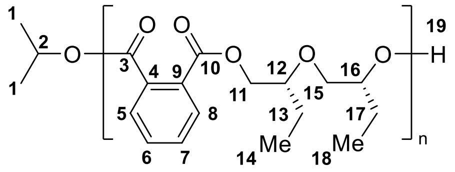

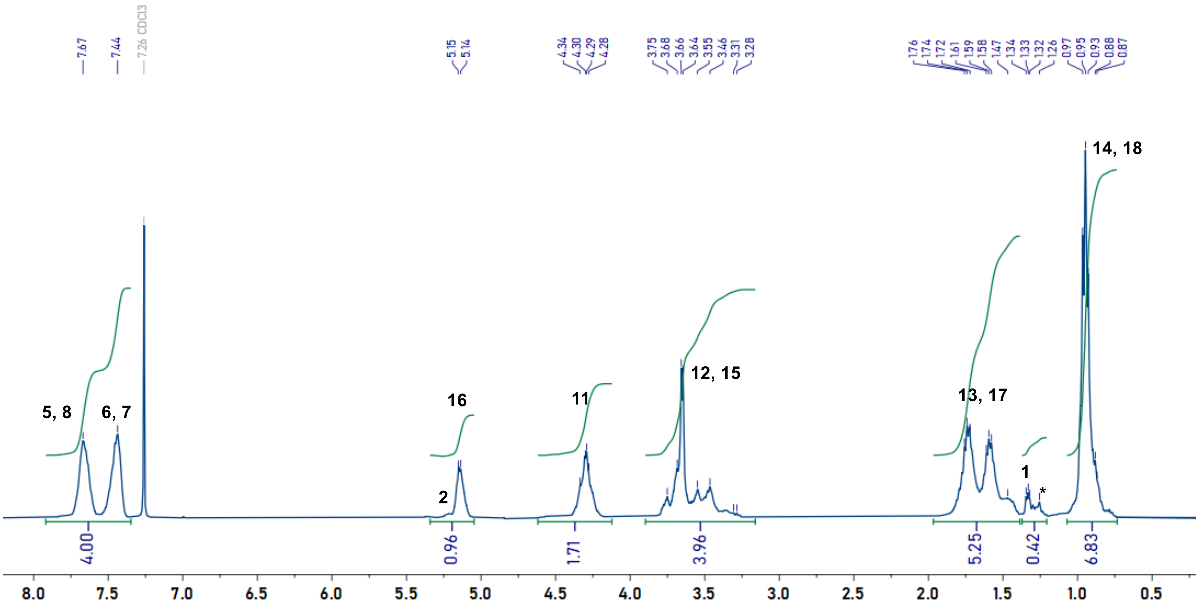

Figure S35: ^{1}H NMR spectrum from the reaction of [Cat]:[PA]:[(*R*)-BO] = 1: 50: 1150, (work-up in MeOH/ CH_2Cl_2). Spectrum corresponds to Table 1, **P7** (400 MHz, $CDCl_3$).

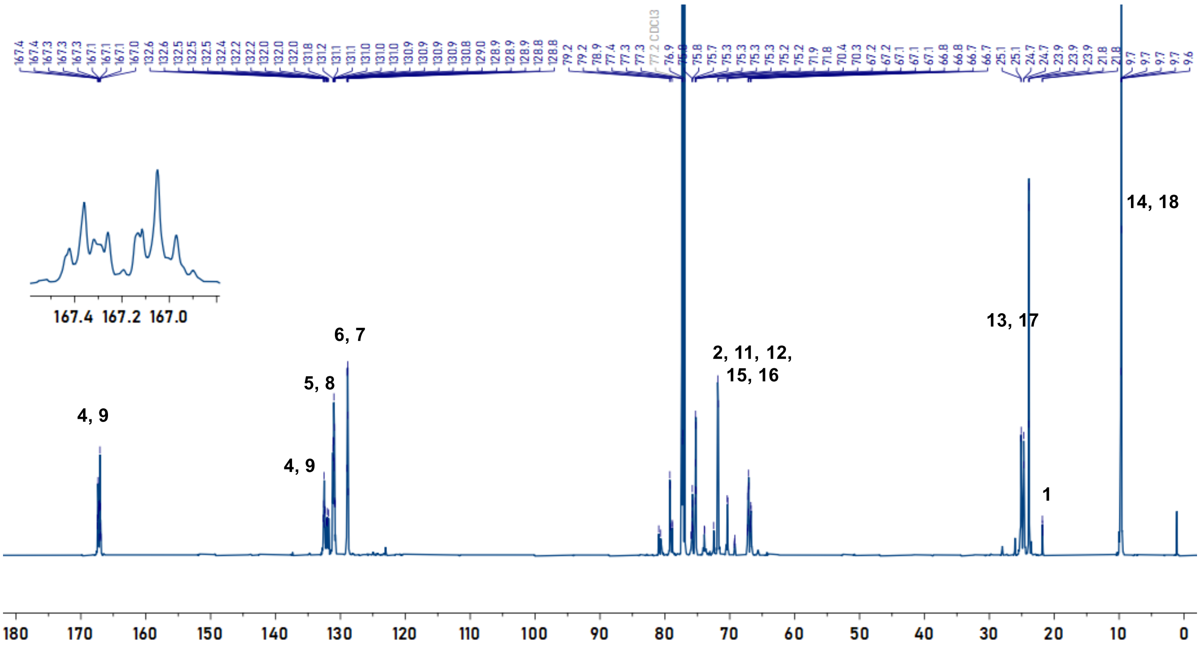

Figure S36: ^{13}C{^{1}H} NMR spectrum from the reaction of [Cat]:[PA]:[(*R*)-BO] = 1: 50: 1150, Table 1, **P7** (151 MHz, $CDCl_3$).

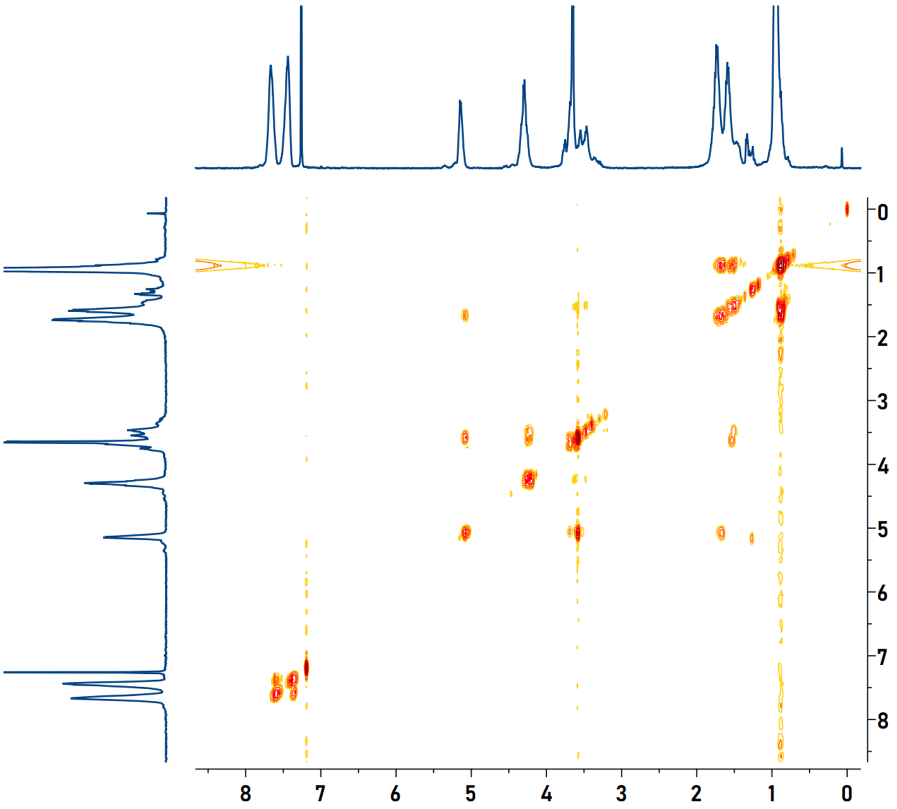

Figure S37: ^{1}H COSY NMR spectrum from the reaction of [Cat]:[PA]:[(*R*)-BO] = 1: 50: 1150, Table 1, **P7** (400 MHz, $CDCl_3$).

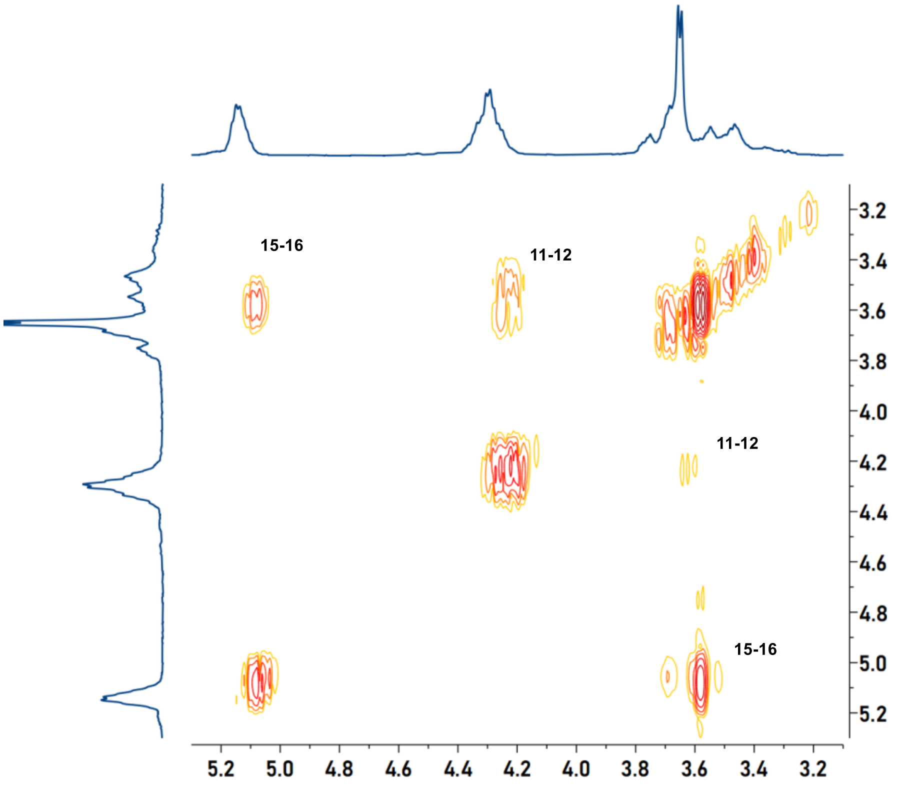

Figure S38: Magnified ^{1}H COSY NMR spectrum from the reaction of [Cat]:[PA]:[(*R*)-BO] = 1: 50: 1150, Table 1, **P7** (400 MHz, $CDCl_3$).

4.1.8 NMR Spectra for P8

P(PA,(*S*)-BO,(*S*)-BO)

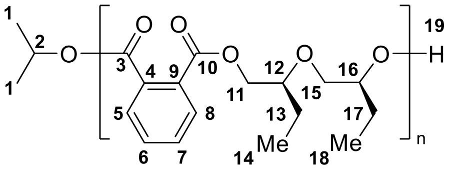

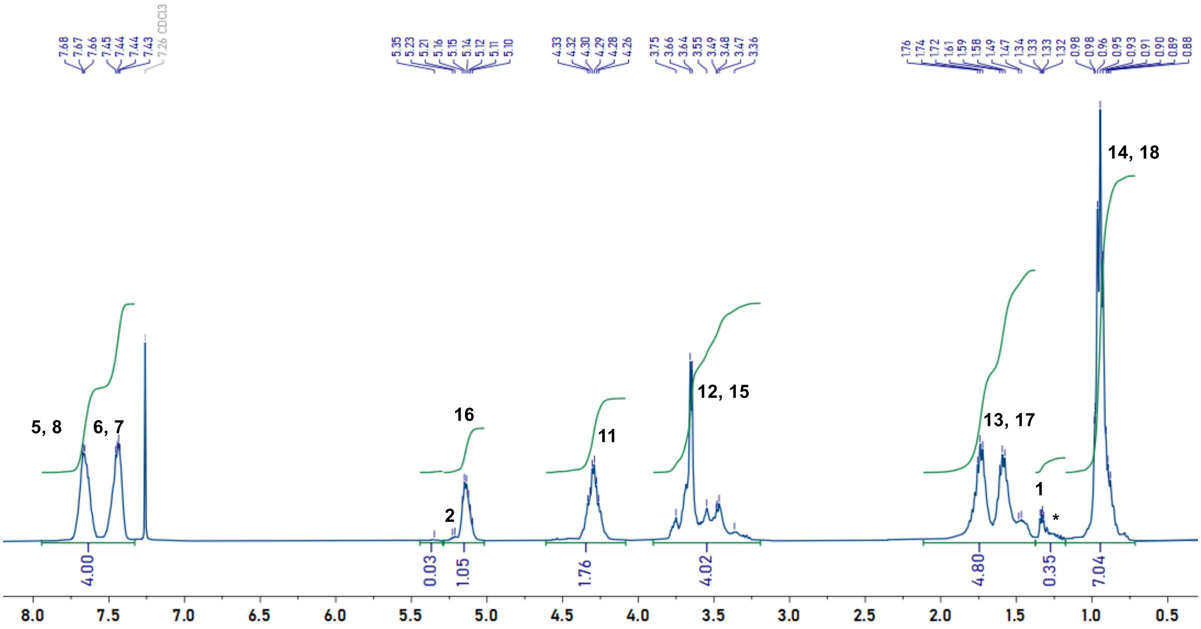

Figure S39: ^{1}H NMR spectrum from the reaction of [Cat]:[PA]:[(*S*)-BO] = 1: 50: 1150, (work-up in MeOH/ CH_2Cl_2). Spectrum corresponds to Table 1, **P8** (400 MHz, $CDCl_3$).

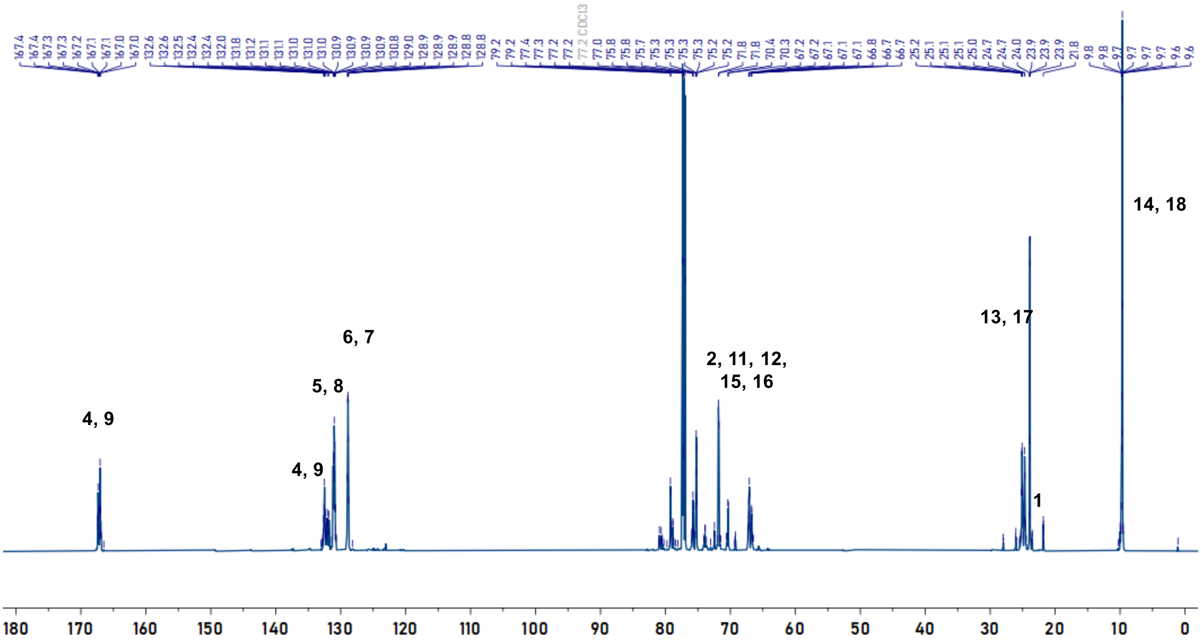

Figure S40: ^{13}C{^{1}H} NMR spectrum from the reaction of [Cat]:[PA]:[(*S*)-BO] = 1: 50: 1150, Table 1, **P8** (151 MHz, $CDCl_3$).

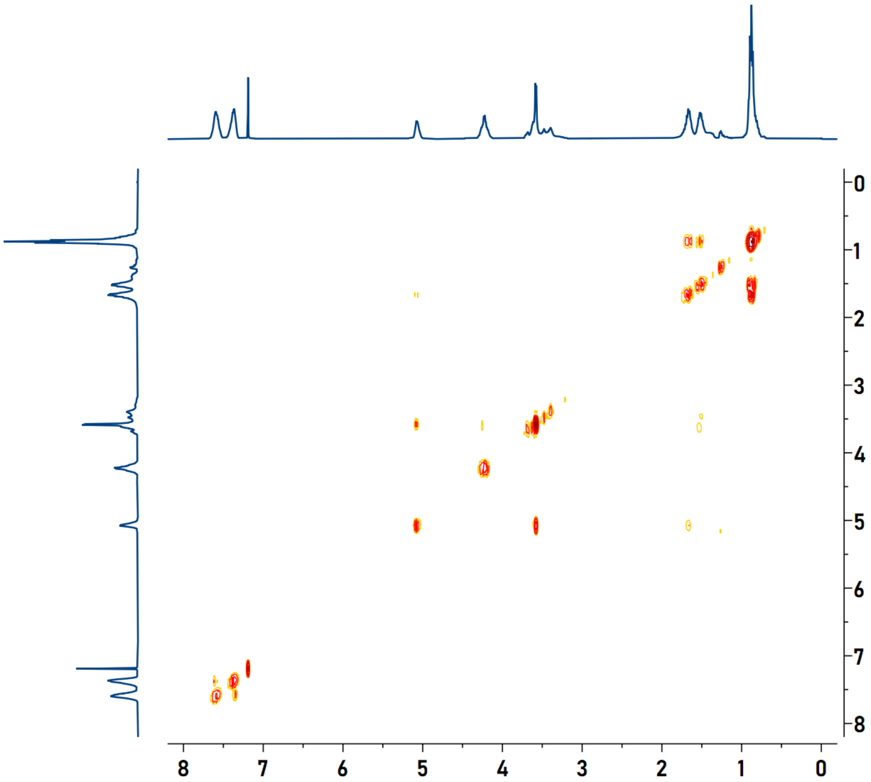

Figure S41: ^{1}H COSY NMR spectrum from the reaction of [Cat]:[PA]:[(*S*)-BO] = 1: 50: 1150, Table 1, **P8** (400 MHz, $CDCl_3$).

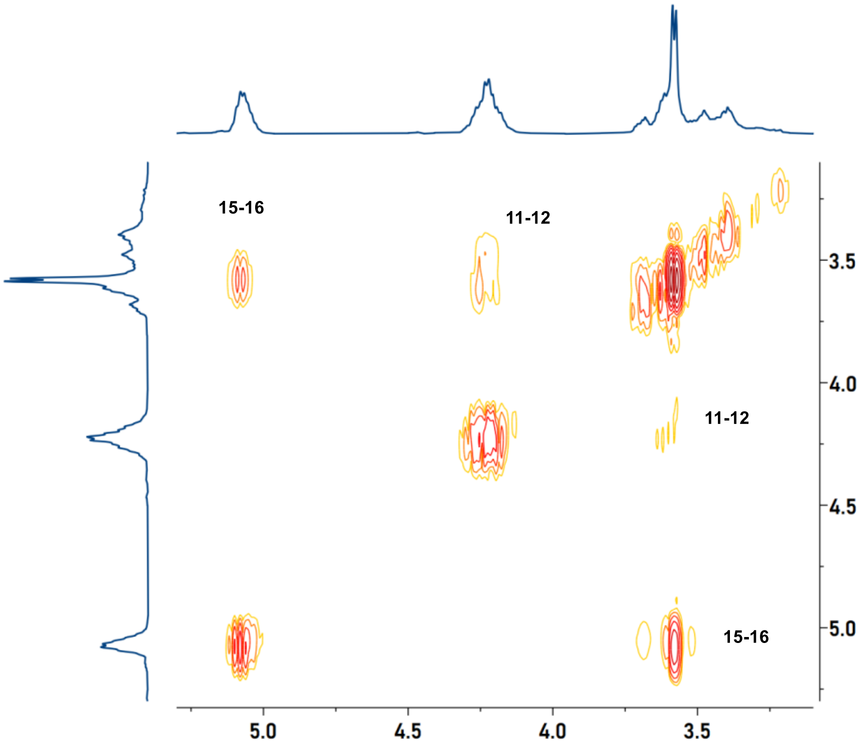

Figure S42: Magnified ^{1}H COSY NMR spectrum from the reaction of [Cat]:[PA]:[(*S*)-BO] = 1: 50: 1150, Table 1, **P8** (400 MHz, $CDCl_3$).

4.1.9 NMR Spectra for P9

P(PA,EDD,EDD)

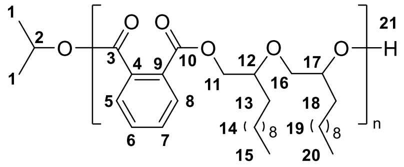

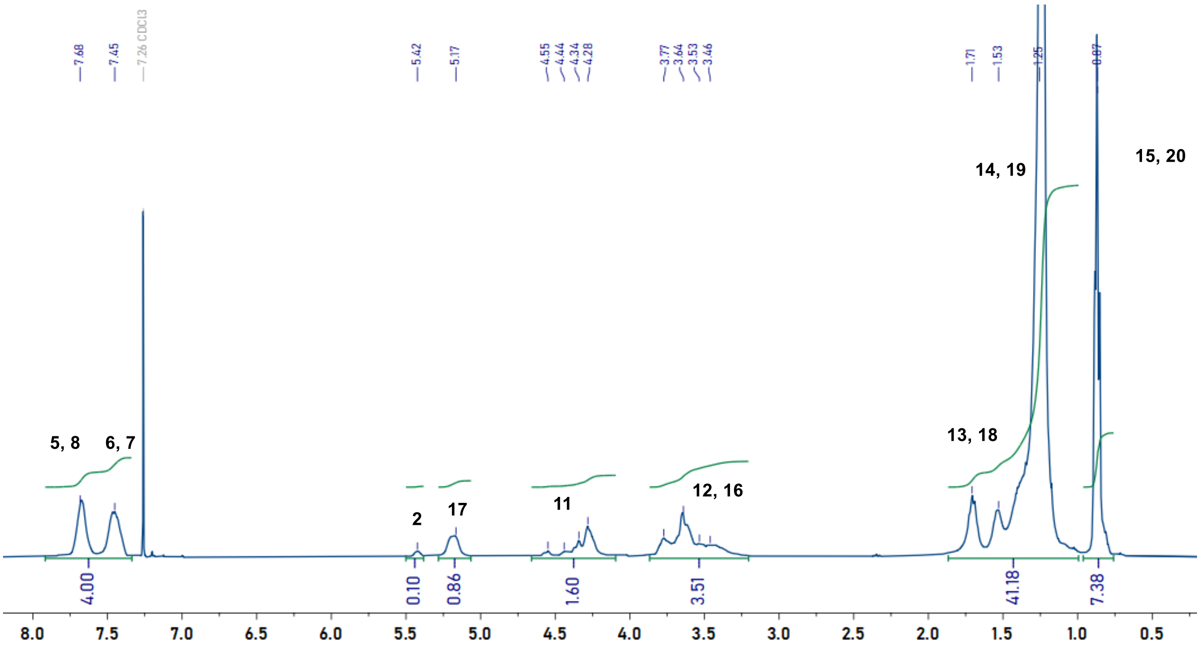

Figure S43: ^{1}H NMR spectrum from the reaction of [Cat]:[PA]:[EDD] = 1: 50: 643, (work-up in MeOH/ CH_2Cl_2). Spectrum corresponds to Table 1, **P9** (400 MHz, $CDCl_3$).

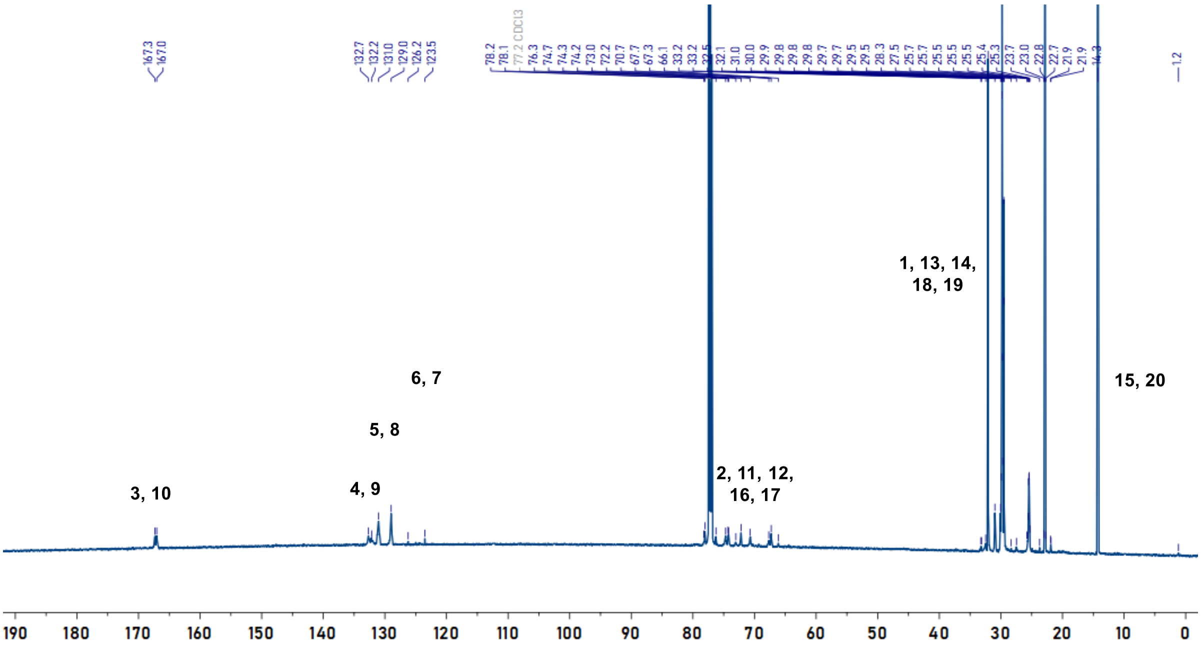

Figure S44: $^{13}C\{^{1}H\}$ NMR spectrum from the reaction of [Cat]:[PA]:[EDD] = 1: 50: 643, Table 1, **P9** (151 MHz, $CDCl_3$).

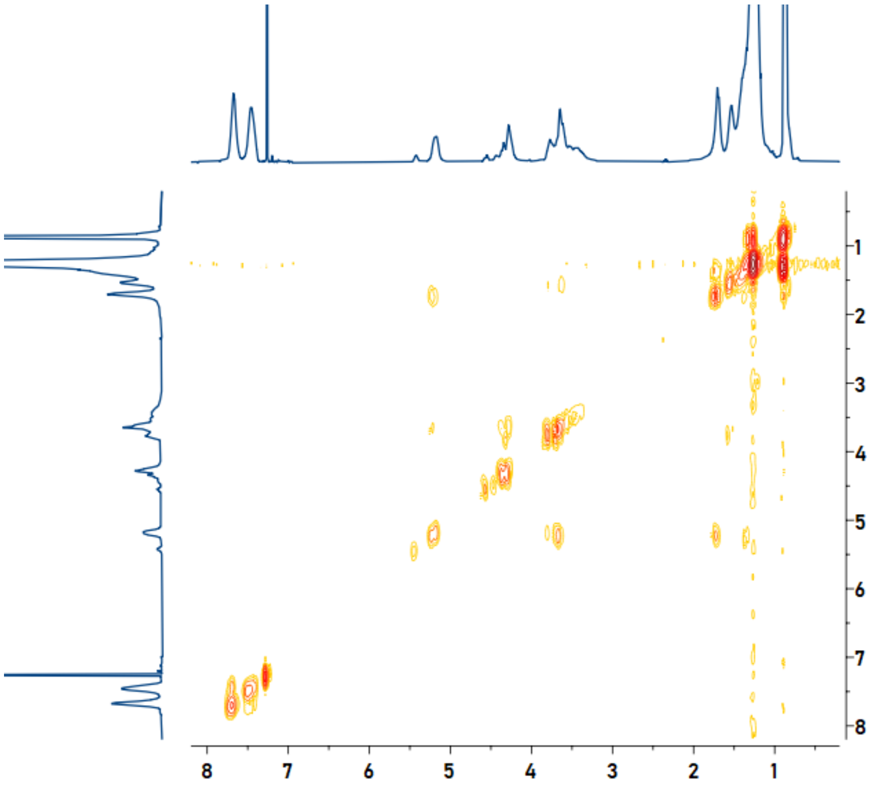

Figure S45: ^{1}H COSY NMR spectrum from the reaction of [Cat]:[PA]:[EDD] = 1: 50: 643, Table 1, **P9** (400 MHz, $CDCl_3$).

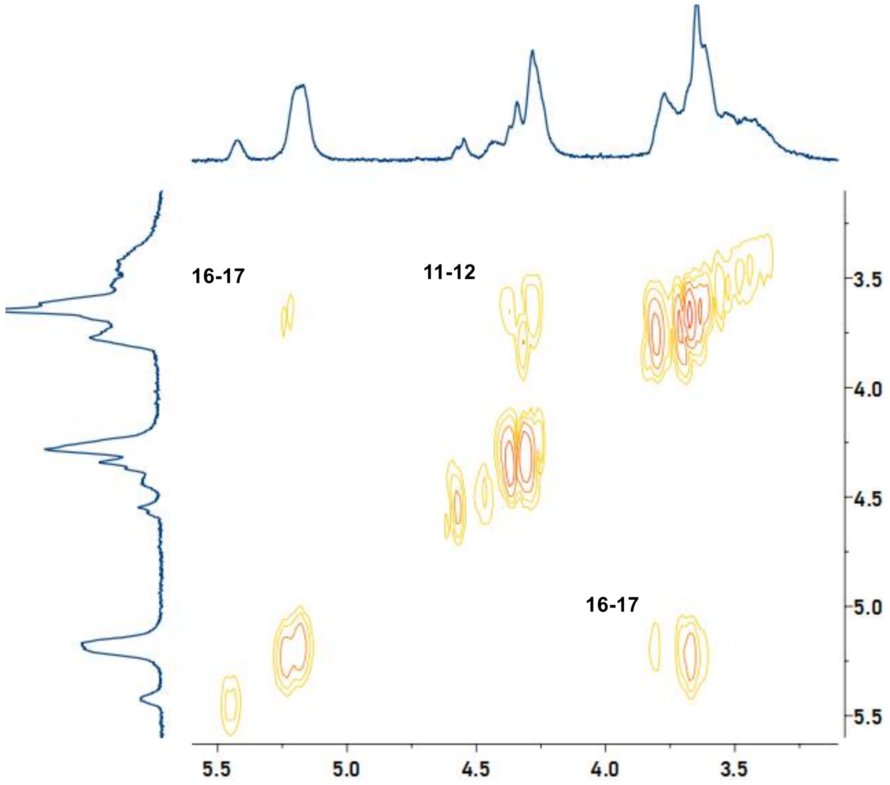

Figure S46: ^{1}H COSY NMR spectrum from the reaction of [Cat]:[PA]:[EDD] = 1: 50: 643, Table 1, **P9** (400 MHz, $CDCl_3$).

4.1.10 NMR Spectra for P10

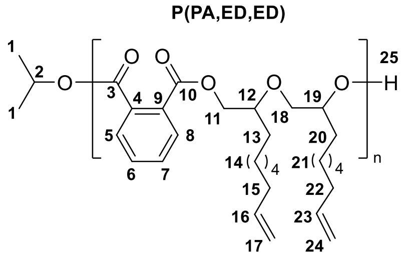

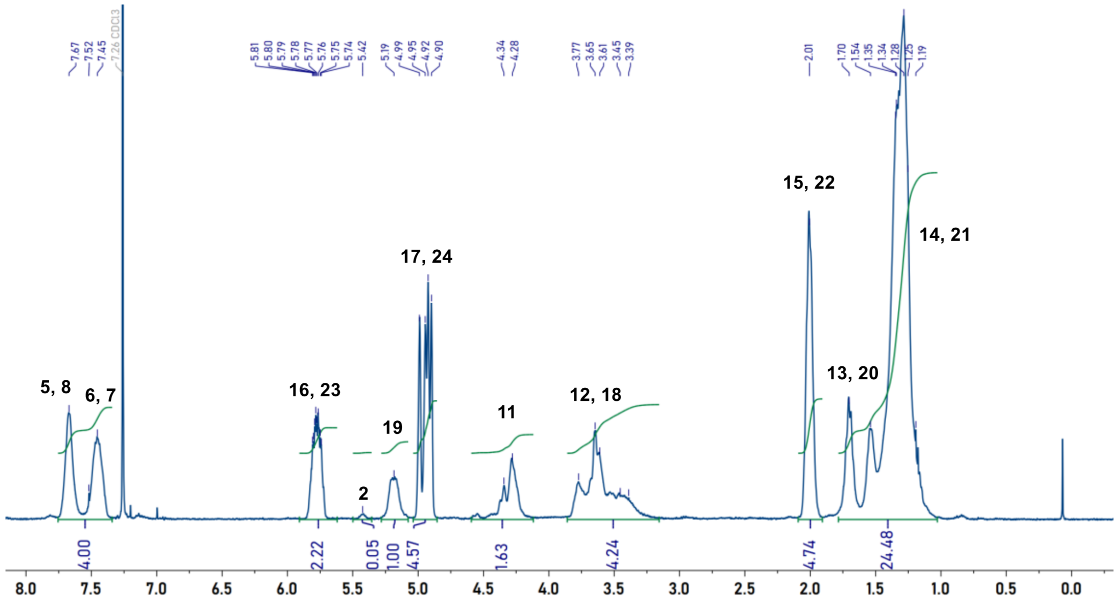

Figure S47: ^{1}H NMR spectrum from the reaction of [Cat]:[PA]:[ED] = 1: 50: 770, (work-up in MeOH/ CH_2Cl_2). Spectrum corresponds to Table 1, **P10** (400 MHz, $CDCl_3$).

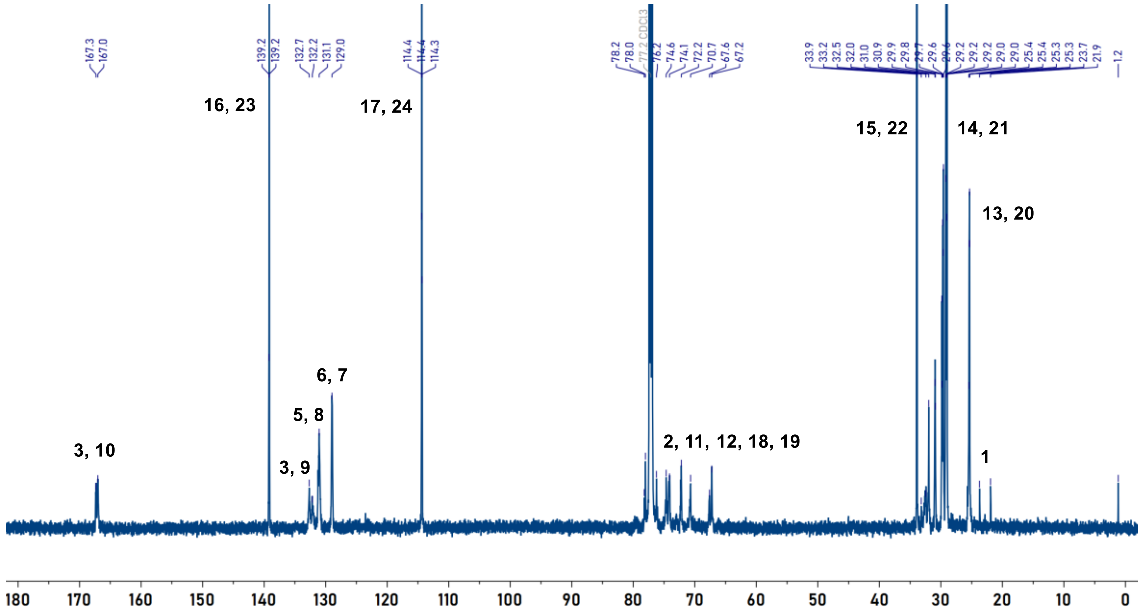

Figure S48: ^{13}C{^{1}H} NMR spectrum from the reaction of [Cat]:[PA]:[ED] = 1: 50: 770, Table 1, **P10** (151 MHz, $CDCl_3$).

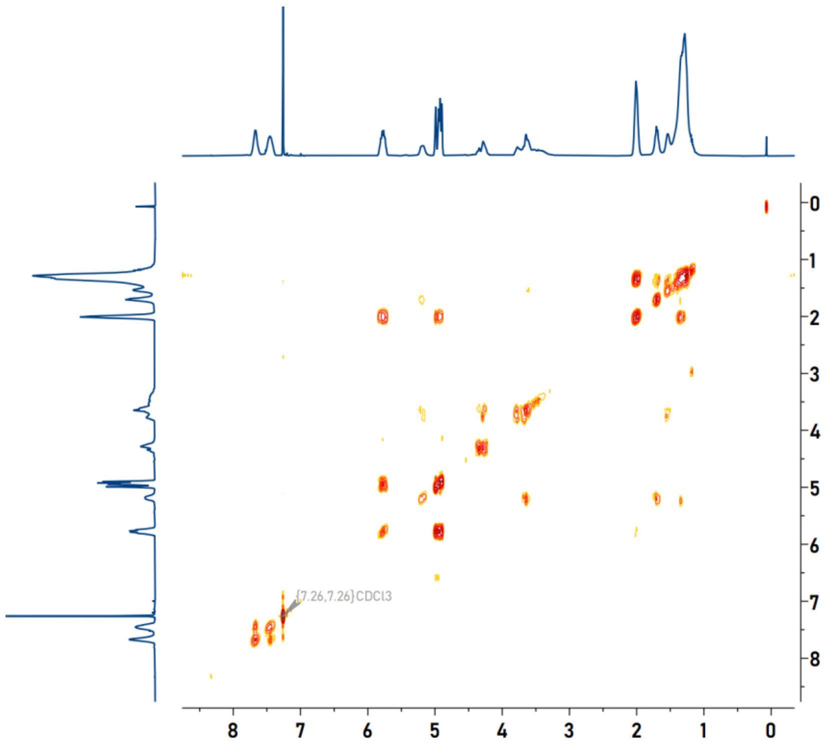

Figure S49: ^{1}H COSY NMR spectrum from the reaction of [Cat]:[PA]:[ED] = 1: 50: 770, Table 1, **P10** (400 MHz, $CDCl_3$).

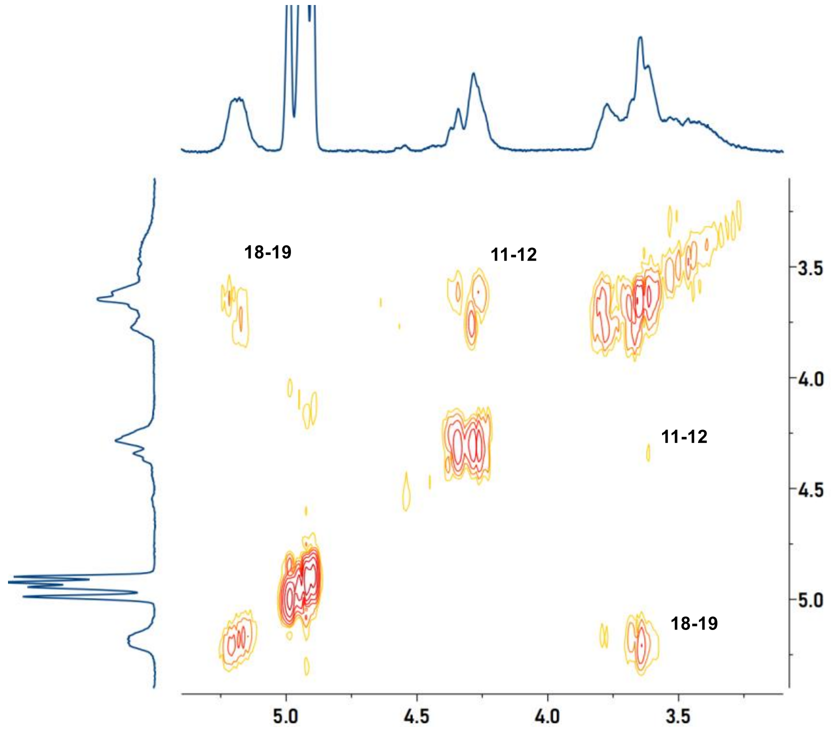

Figure S50: Magnified ^{1}H COSY NMR spectrum from the reaction of [Cat]:[PA]:[ED] = 1: 50: 770, Table 1, **P10** (400 MHz, $CDCl_3$).

4.1.11 NMR Spectra for P11

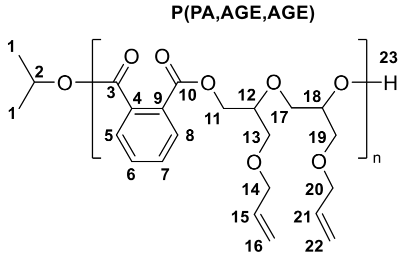

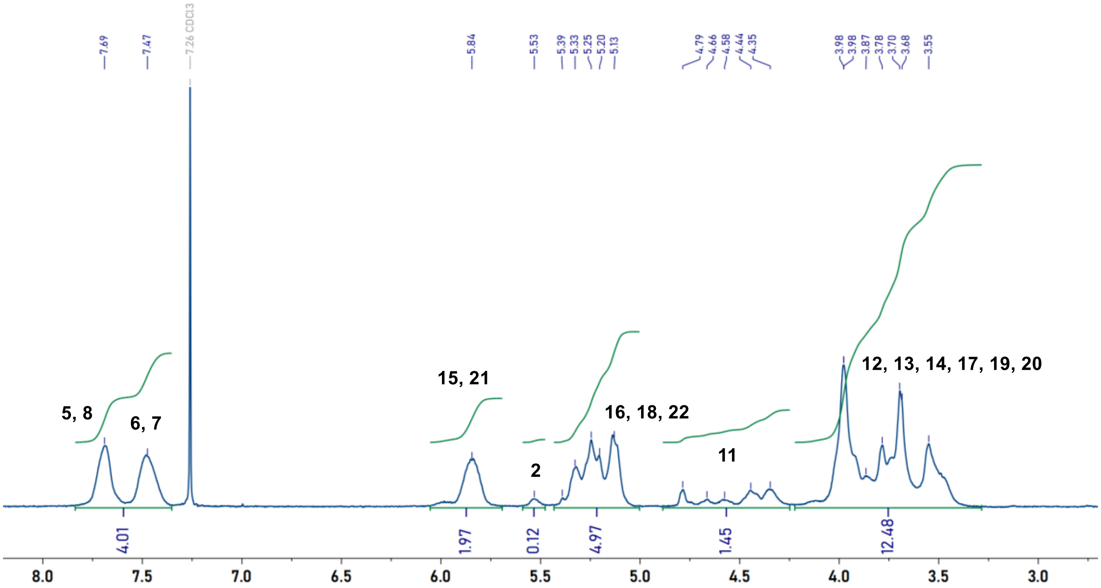

Figure S51: ^{1}H NMR spectrum from the reaction of [Cat]:[PA]:[AGE] = 1: 50: 911, (work-up in MeOH/ CH_2Cl_2). Spectrum corresponds to Table 1, **P11** (400 MHz, $CDCl_3$).

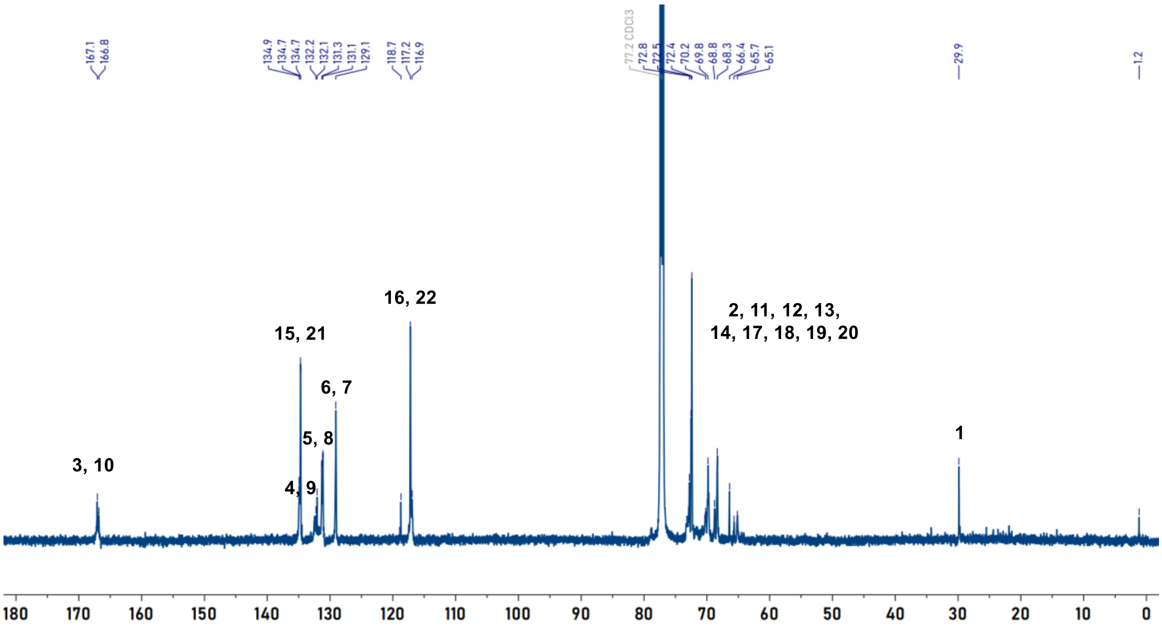

Figure S52: ^{13}C{^{1}H} NMR spectrum from the reaction of [Cat]:[PA]:[AGE] = 1: 50: 911, Table 1, **P11** (151 MHz, $CDCl_3$).

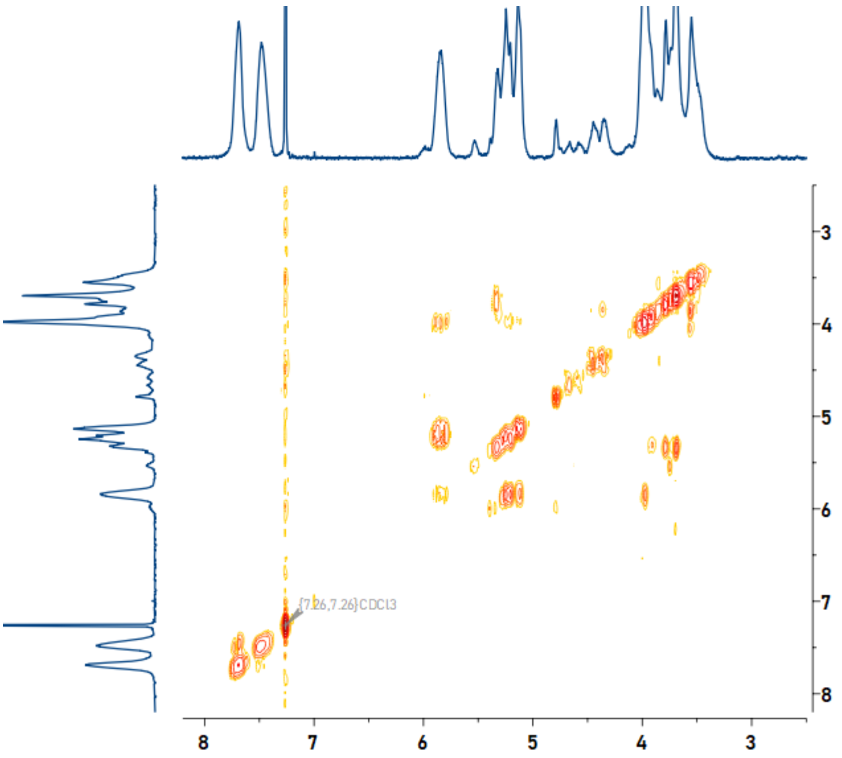

Figure S53: ^{1}H COSY NMR spectrum from the reaction of [Cat]:[PA]:[AGE] = 1: 50: 911, Table 1, **P11** (400 MHz, $CDCl_3$).

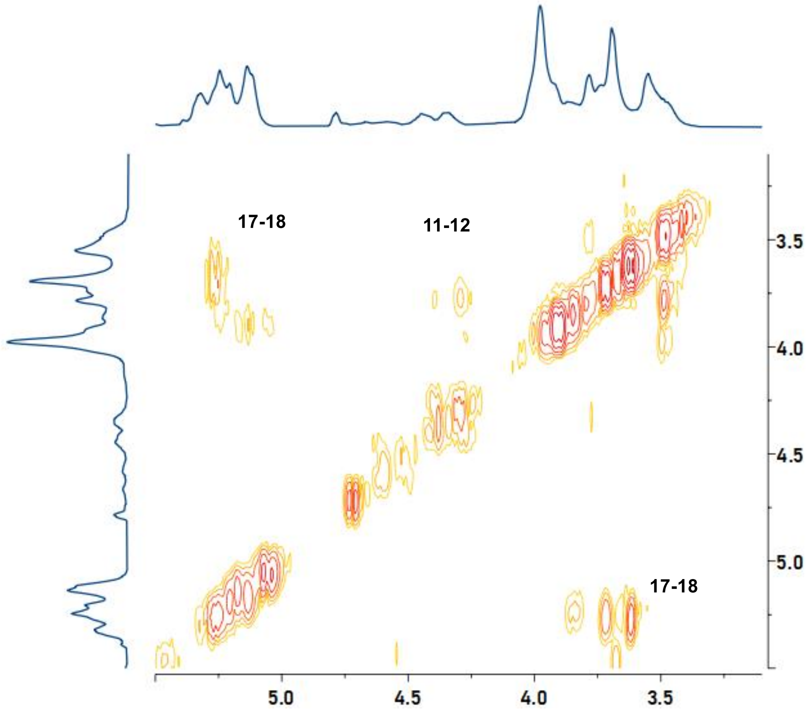

Figure S54: Magnified ^{1}H COSY NMR spectrum from the reaction of [Cat]:[PA]:[AGE] = 1: 50: 911, Table 1, **P11** (400 MHz, $CDCl_3$).

4.1.12 NMR Spectra for P12

P(PA,EHGE,EHGE)

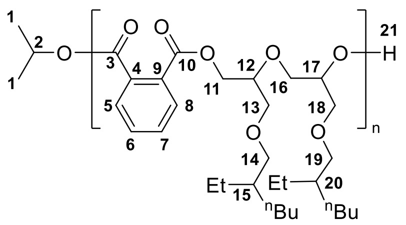

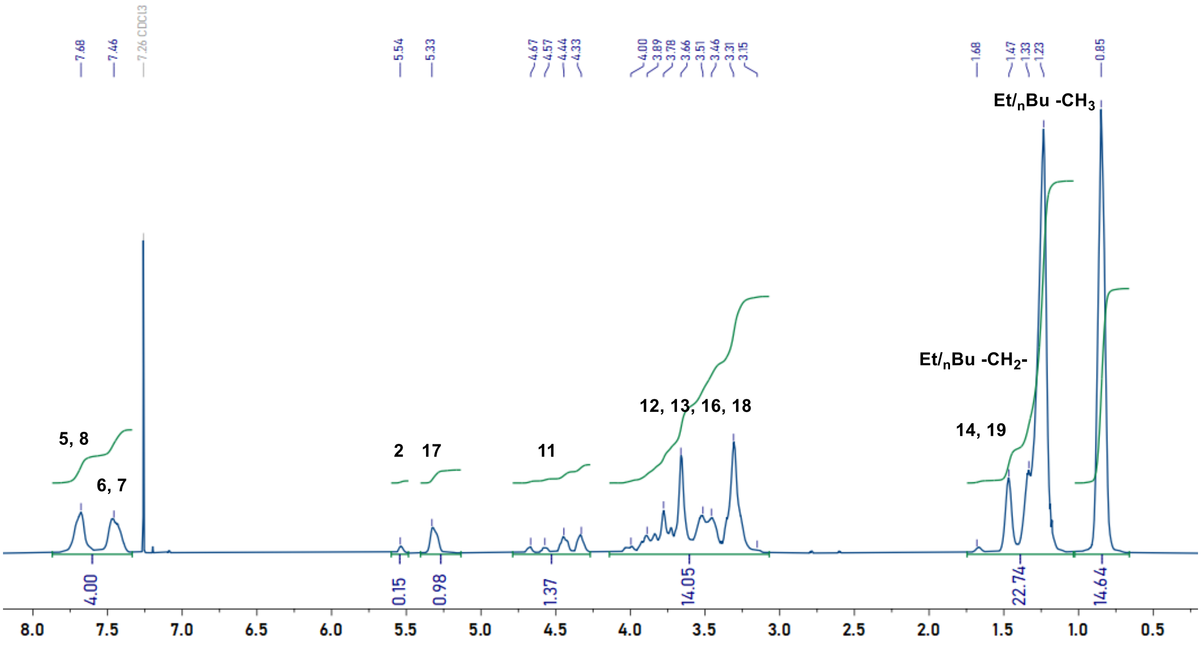

Figure S55: ^{1}H NMR spectrum from the reaction of [Cat]:[PA]:[EHGE] = 1: 50: 602, (work-up in MeOH/ CH_2Cl_2). Spectrum corresponds to Table 1, **P12** (400 MHz, $CDCl_3$).

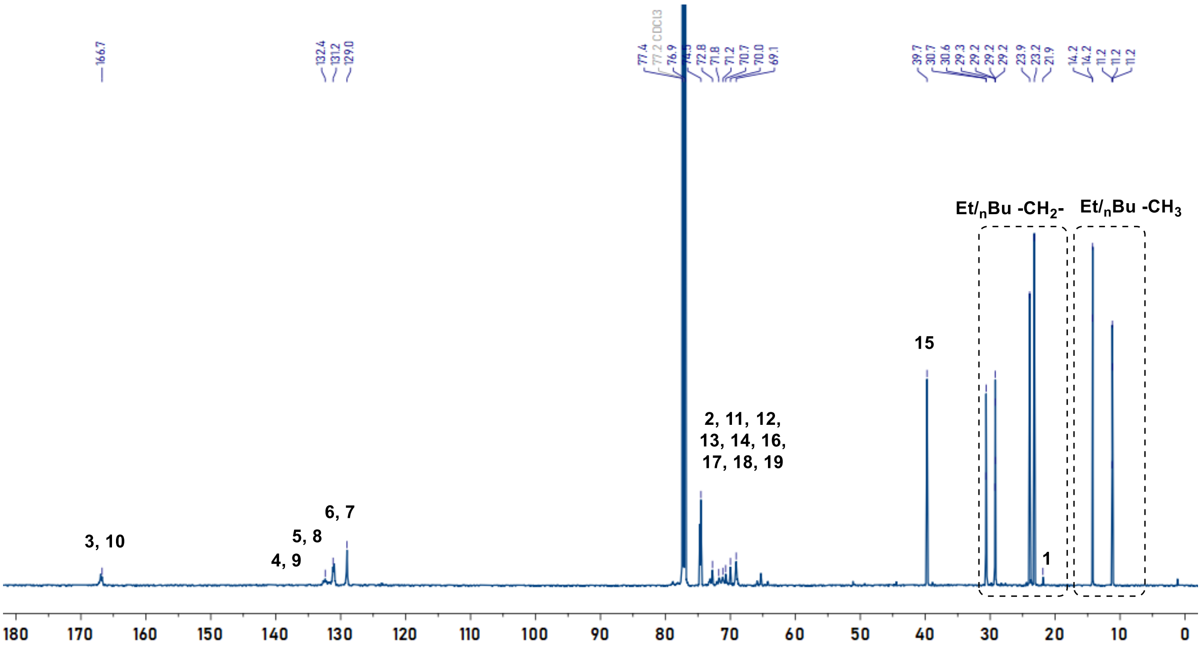

Figure S56: $^{13}C\{^{1}H\}$ NMR spectrum from the reaction of [Cat]:[PA]:[EHGE] = 1: 50: 602, Table 1, **P12** (151 MHz, $CDCl_3$).

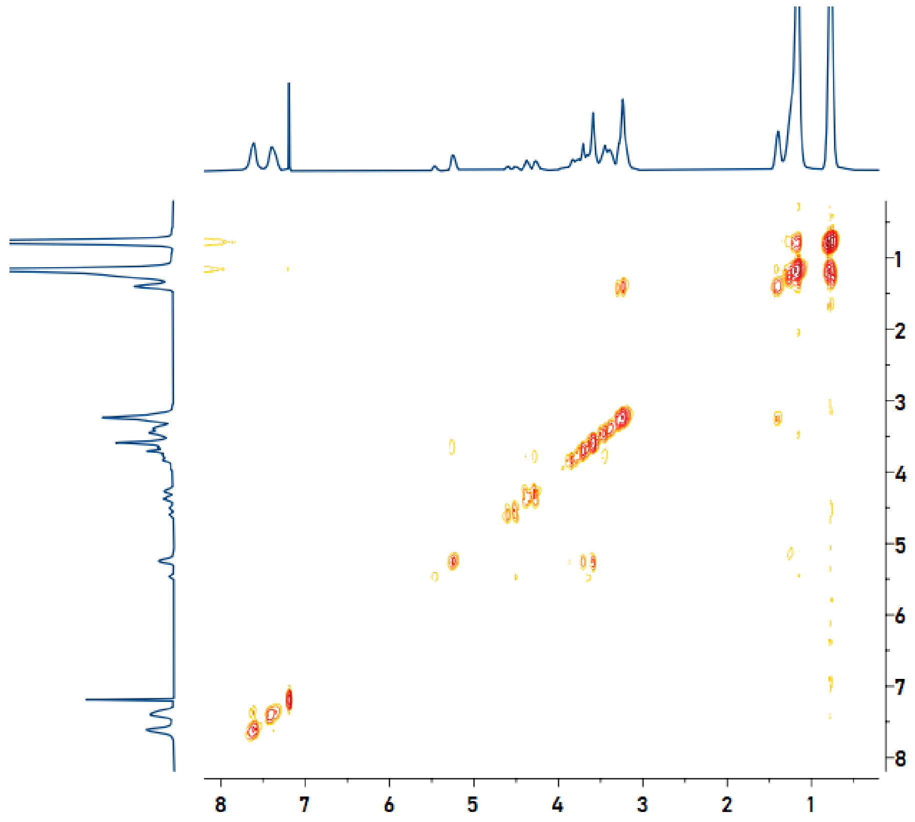

Figure S57: ^{1}H COSY NMR spectrum from the reaction of [Cat]:[PA]:[EHGE] = 1: 50: 602, Table 1, **P12** (400 MHz, $CDCl_3$).

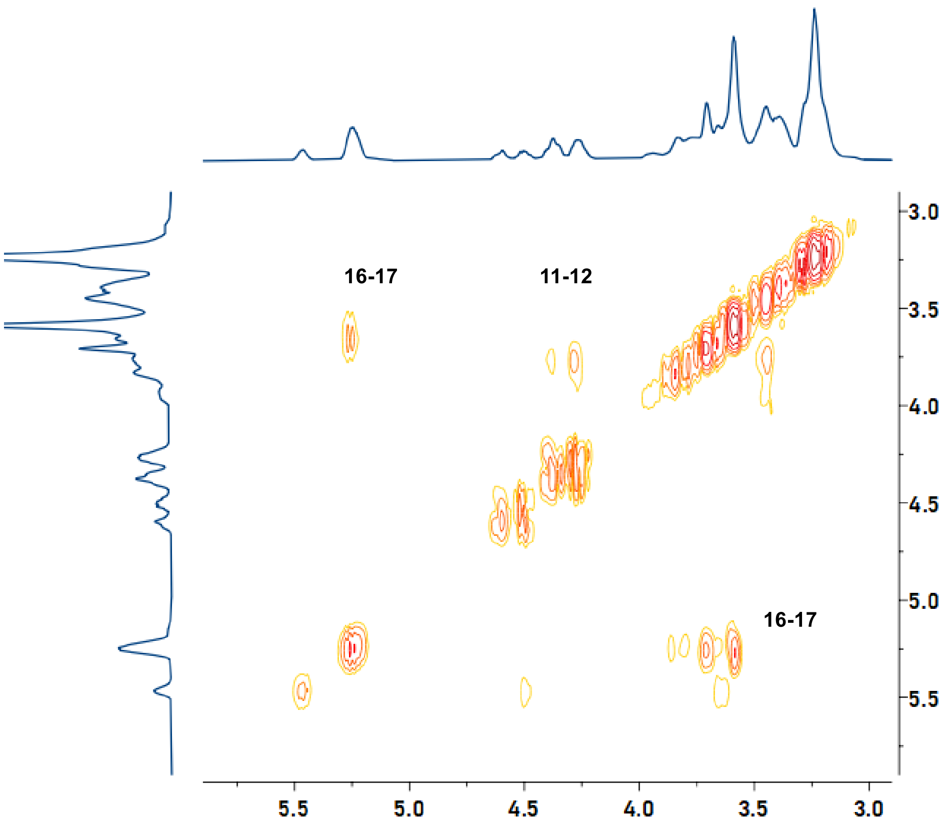

Figure S58: Magnified ^{1}H COSY NMR spectrum from the reaction of [Cat]:[PA]:[EHGE] = 1: 50: 602, Table 1, **P12** (400 MHz, $CDCl_3$).

4.1.13 NMR Spectra for P13

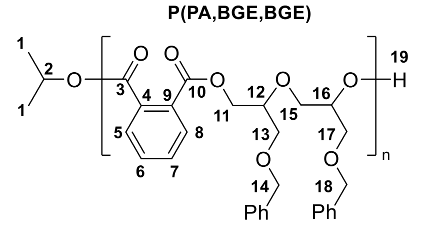

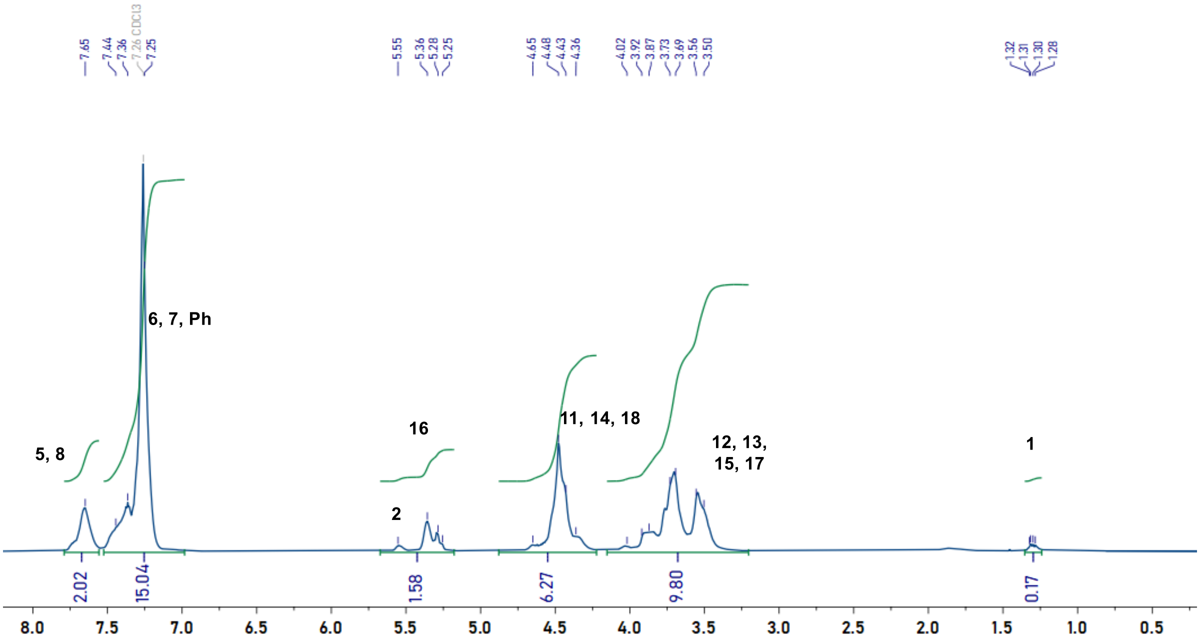

Figure S59: ^{1}H NMR spectrum from the reaction of [Cat]:[PA]:[BGE] = 1: 50: 565, (work-up in MeOH/ CH_2Cl_2). Spectrum corresponds to Table 1, **P13** (400 MHz, $CDCl_3$).

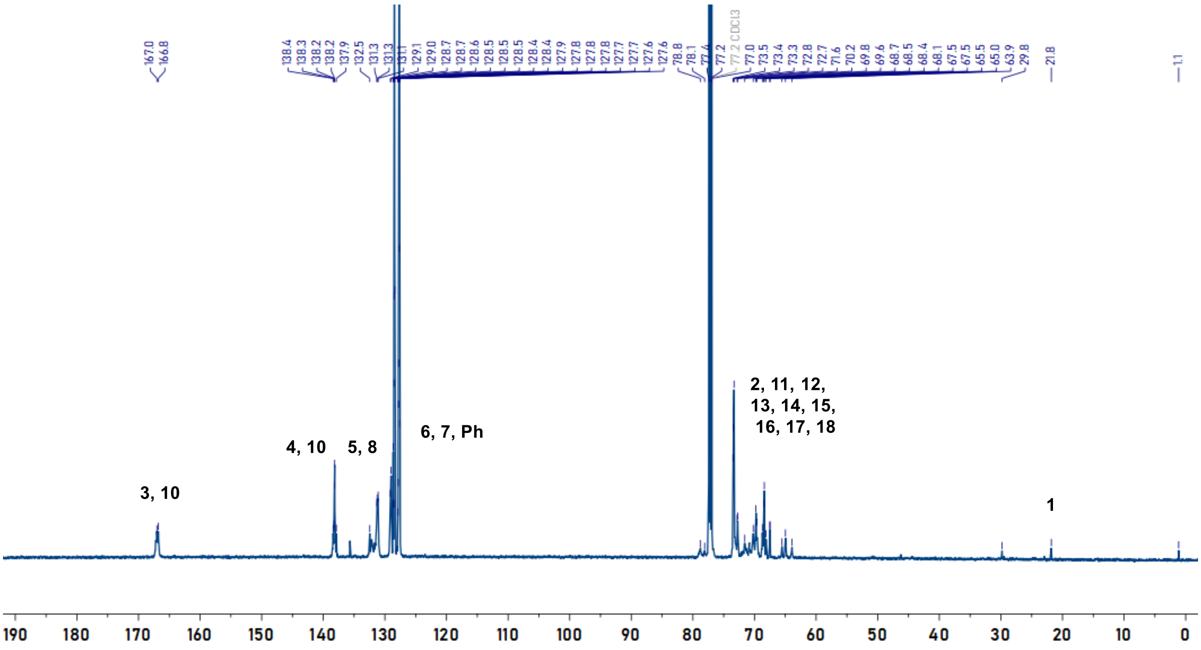

Figure S60: $^{13}C\{^{1}H\}$ NMR spectrum from the reaction of [Cat]:[PA]:[BGE] = 1: 50: 565, Table 1, **P13** (151 MHz, $CDCl_3$).

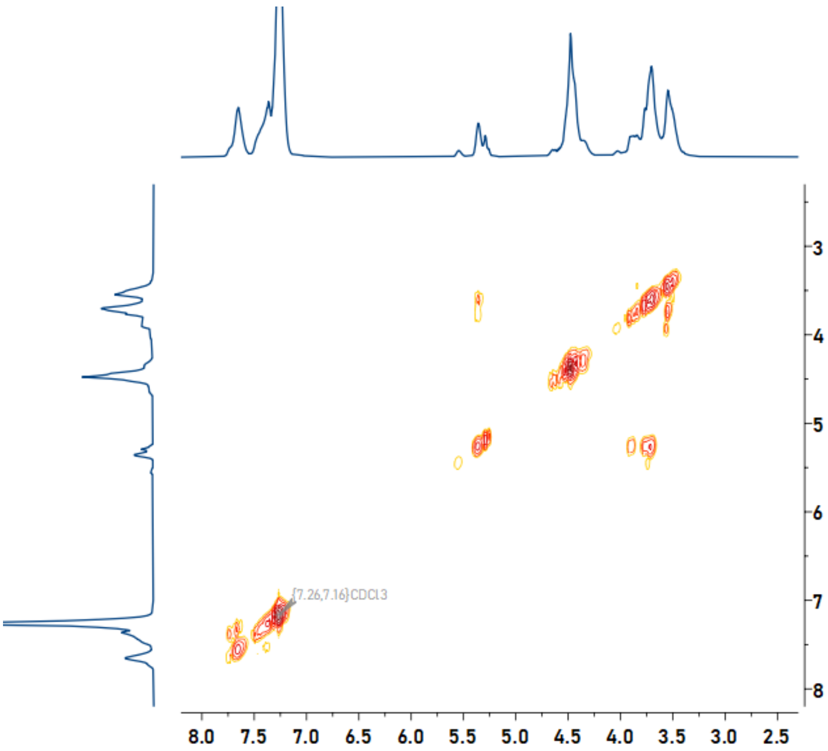

Figure S61: ^{1}H COSY NMR spectrum from the reaction of [Cat]:[PA]:[BGE] = 1: 50: 565, Table 1, **P13** (400 MHz, $CDCl_3$).

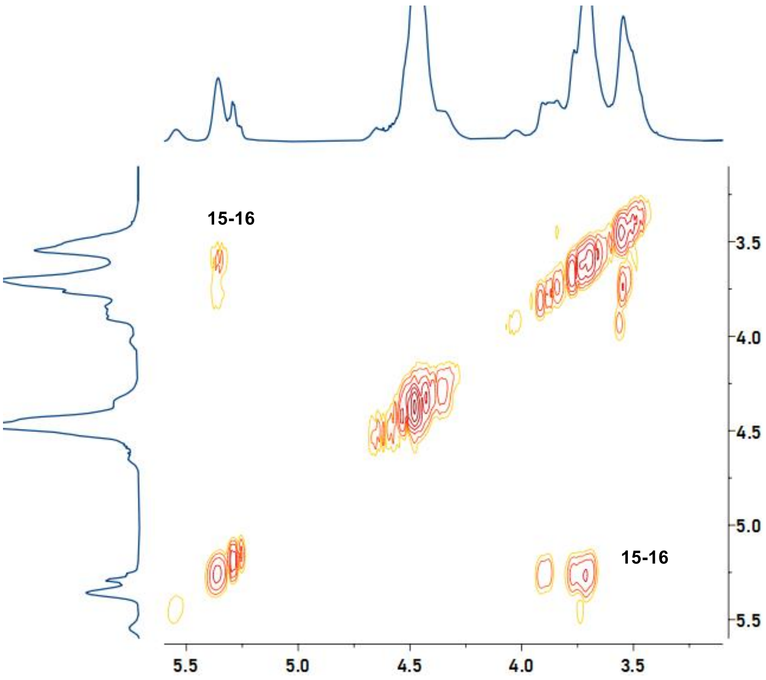

Figure S62: Magnified ^{1}H COSY NMR spectrum from the reaction [Cat]:[PA]:[BGE] = 1: 50: 565, Table 1, **P13** (400 MHz, $CDCl_3$).

4.1.14 NMR Spectra for P14

P14 - P(MA,EDD,EDD)

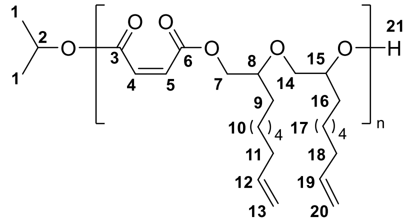

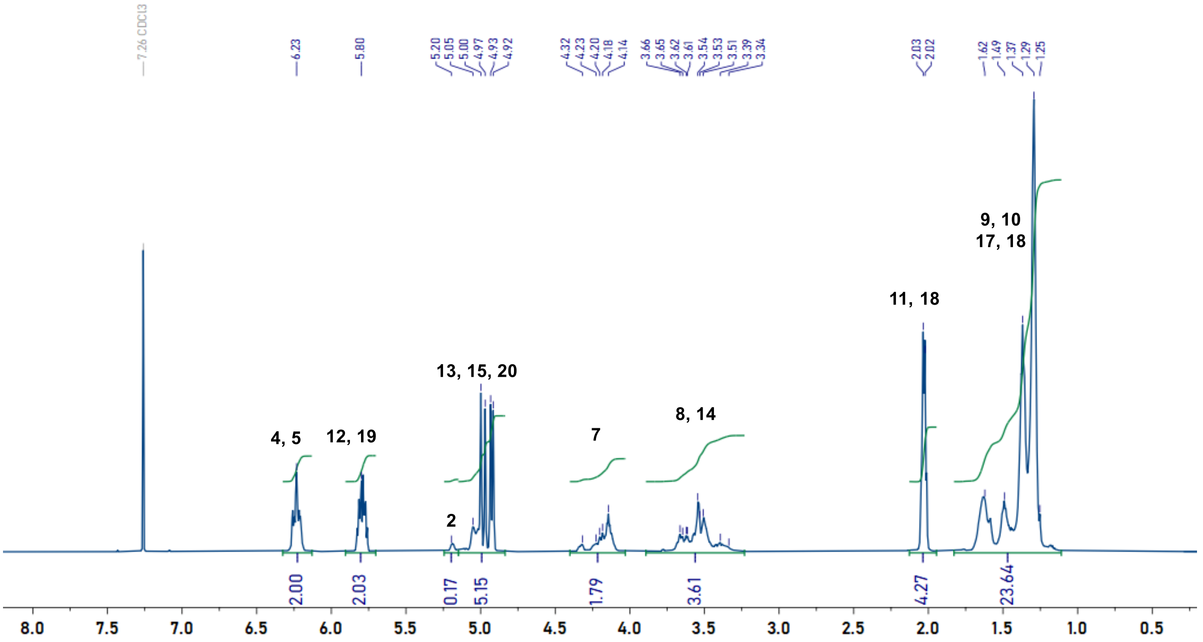

Figure S63: ^{1}H NMR spectrum from the reaction of [Cat]:[MA]:[ED] = 1: 50: 770, (work-up in MeOH/CH_2Cl_2). Spectrum corresponds to Table 1, **P14** (400 MHz, $CDCl_3$).

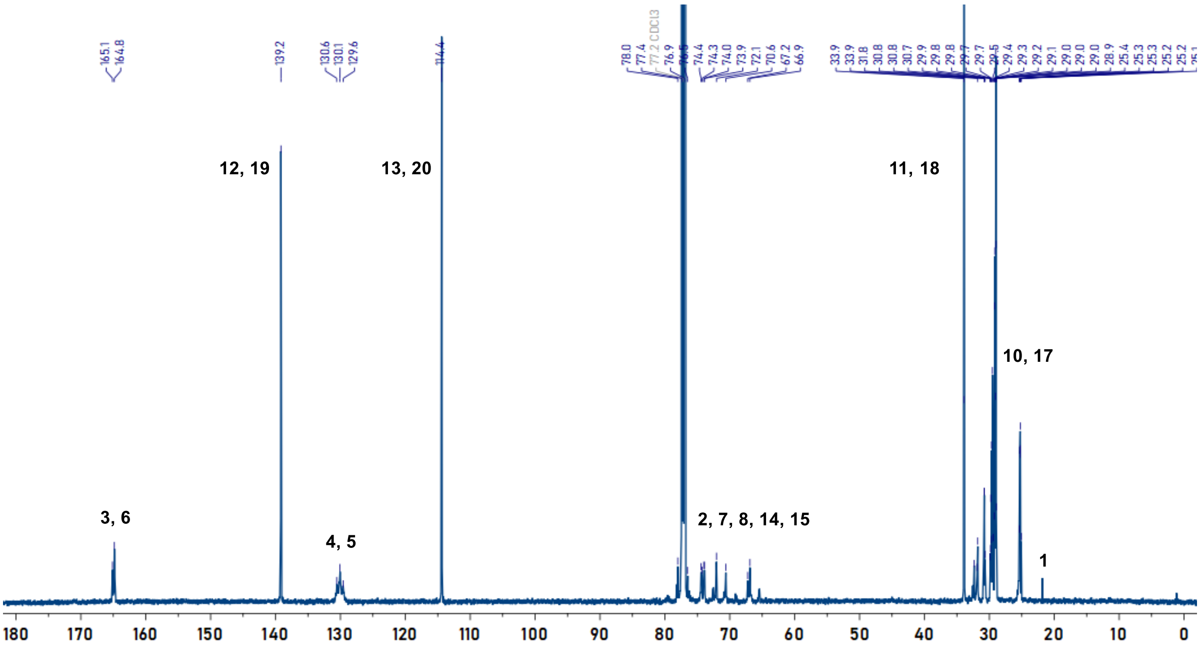

Figure S64: $^{13}C\{^{1}H\}$ NMR spectrum from the reaction of [Cat]:[MA]:[ED] = 1: 50: 770, Table 1, **P14** (151 MHz, $CDCl_3$).

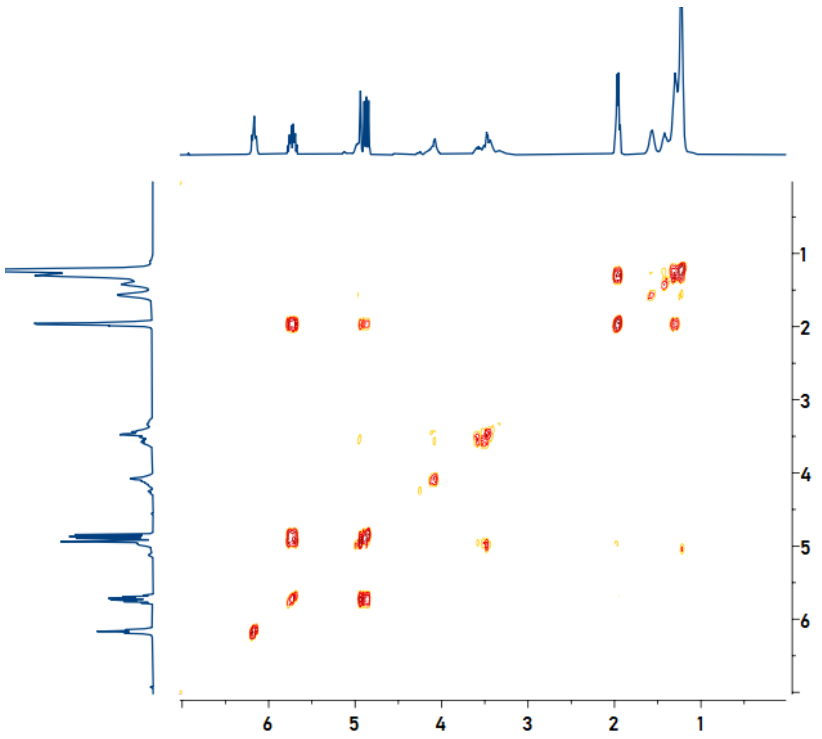

Figure S65: ^{1}H COSY NMR spectrum from the reaction of [Cat]:[MA]:[ED] = 1: 50: 770, Table 1, **P14** (400 MHz, $CDCl_3$).

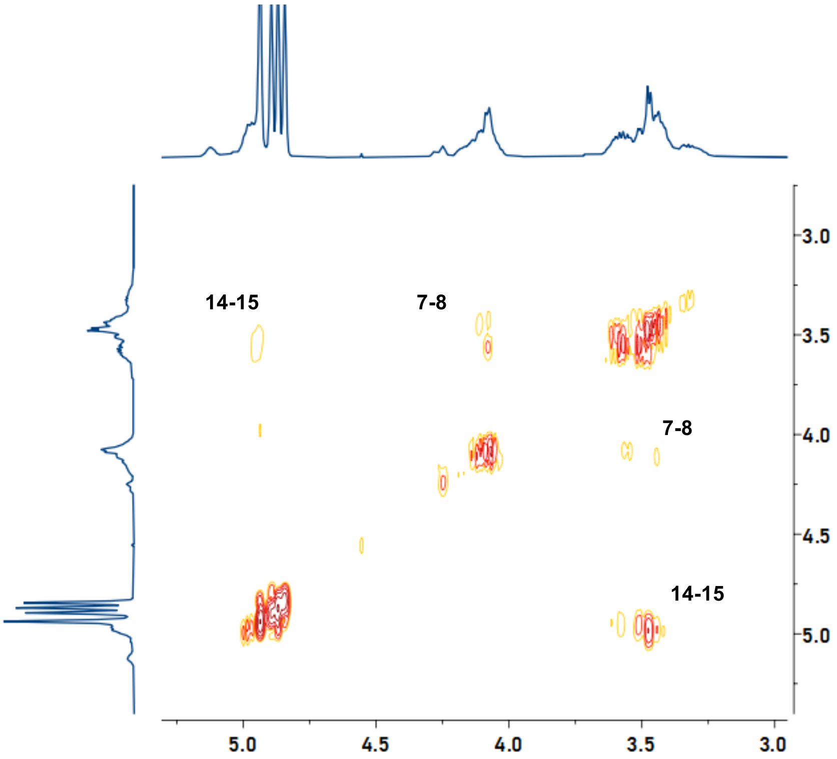

Figure S66: Magnified ^{1}H COSY NMR spectrum from the reaction of [Cat]:[MA]:[ED] = 1: 50: 770, Table 1, **P14** (400 MHz, $CDCl_3$).

4.2 GPC Data for Polymers Described in Table 1

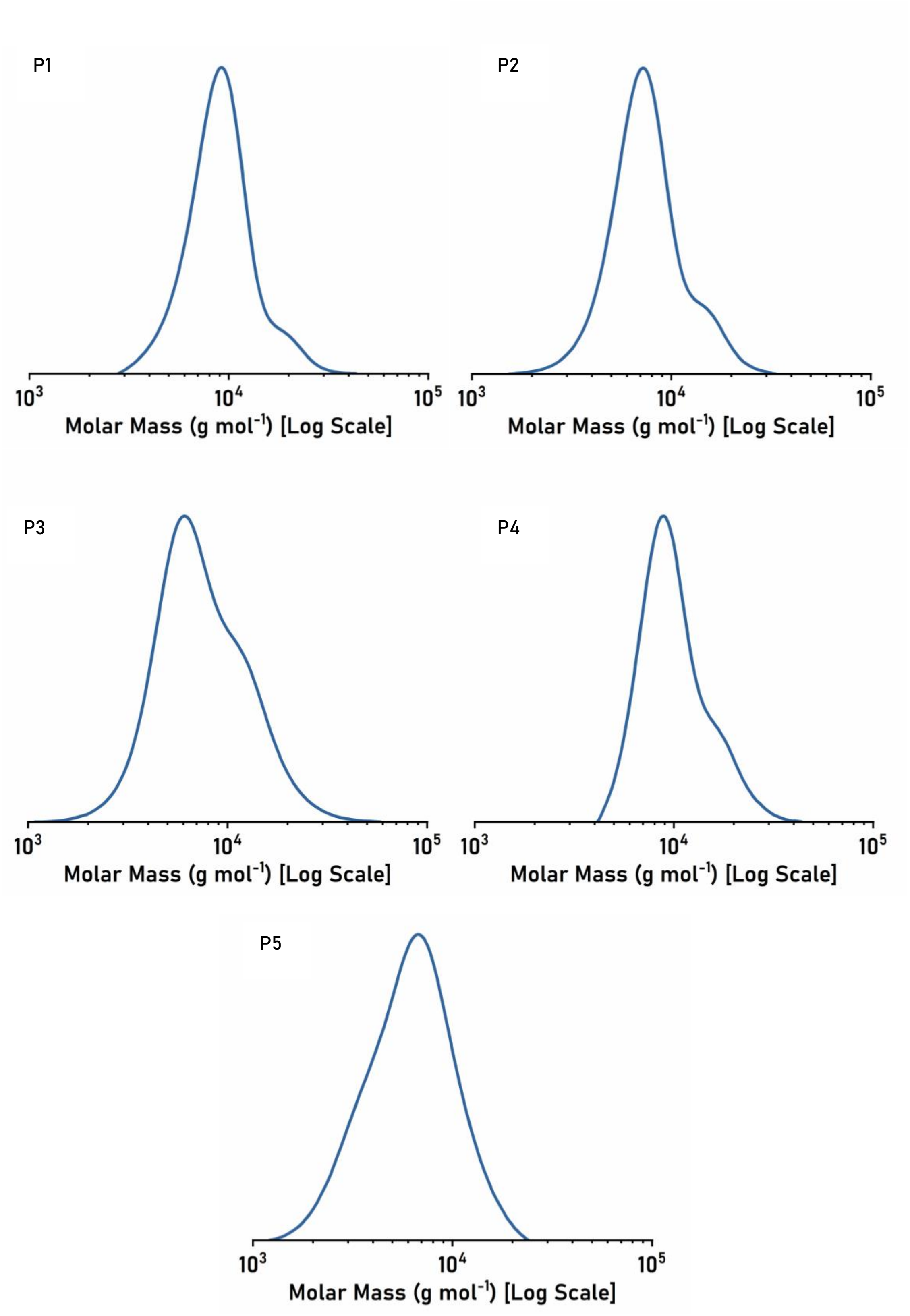

Figure S67: GPC chromatograms for polymers described in Table 1, **P1-5**, read left-to-right, top-to-bottom.

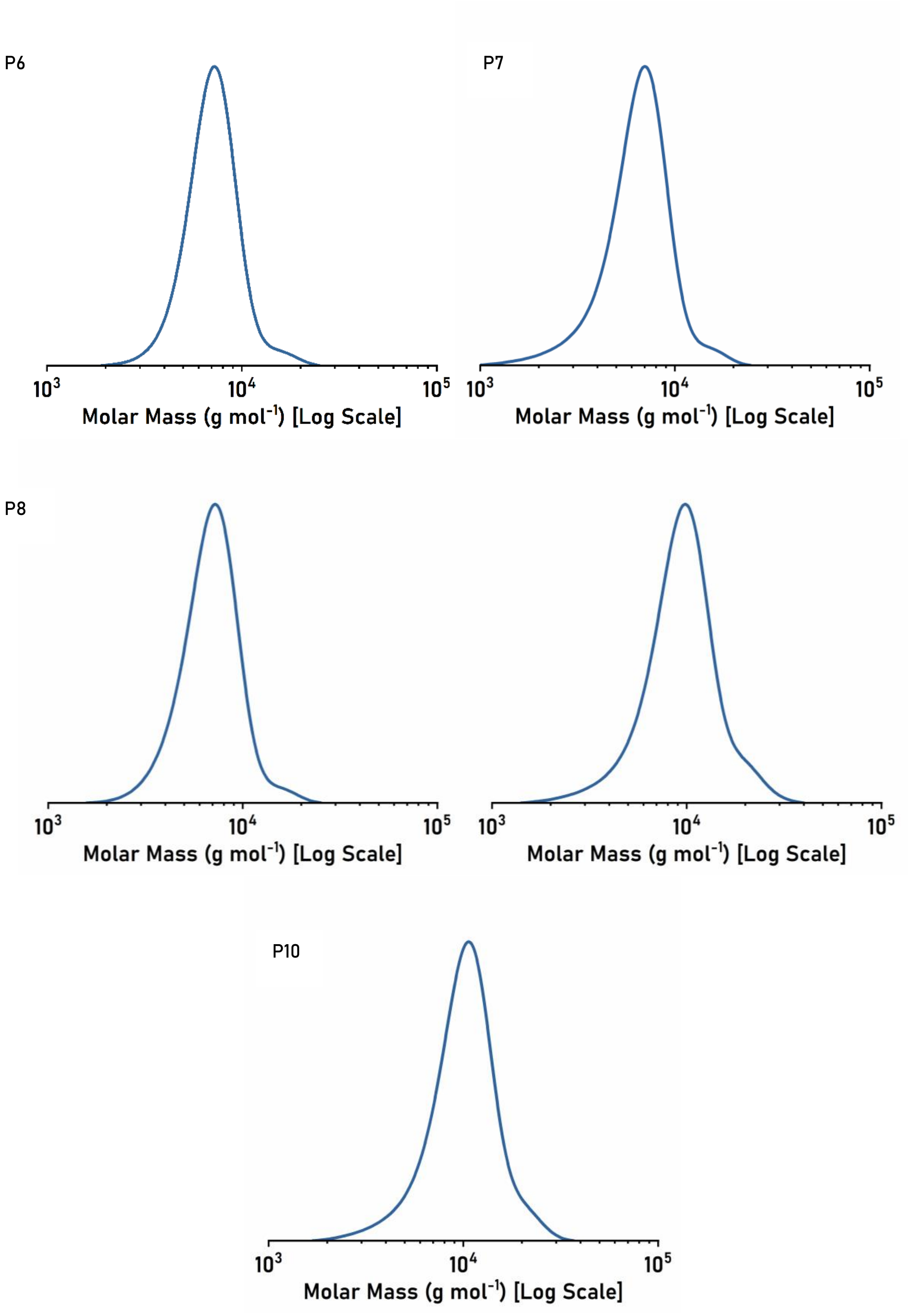

Figure S68: GPC chromatograms for polymers described in Table 1, **P6-10,** read left-to-right, top-to-bottom.

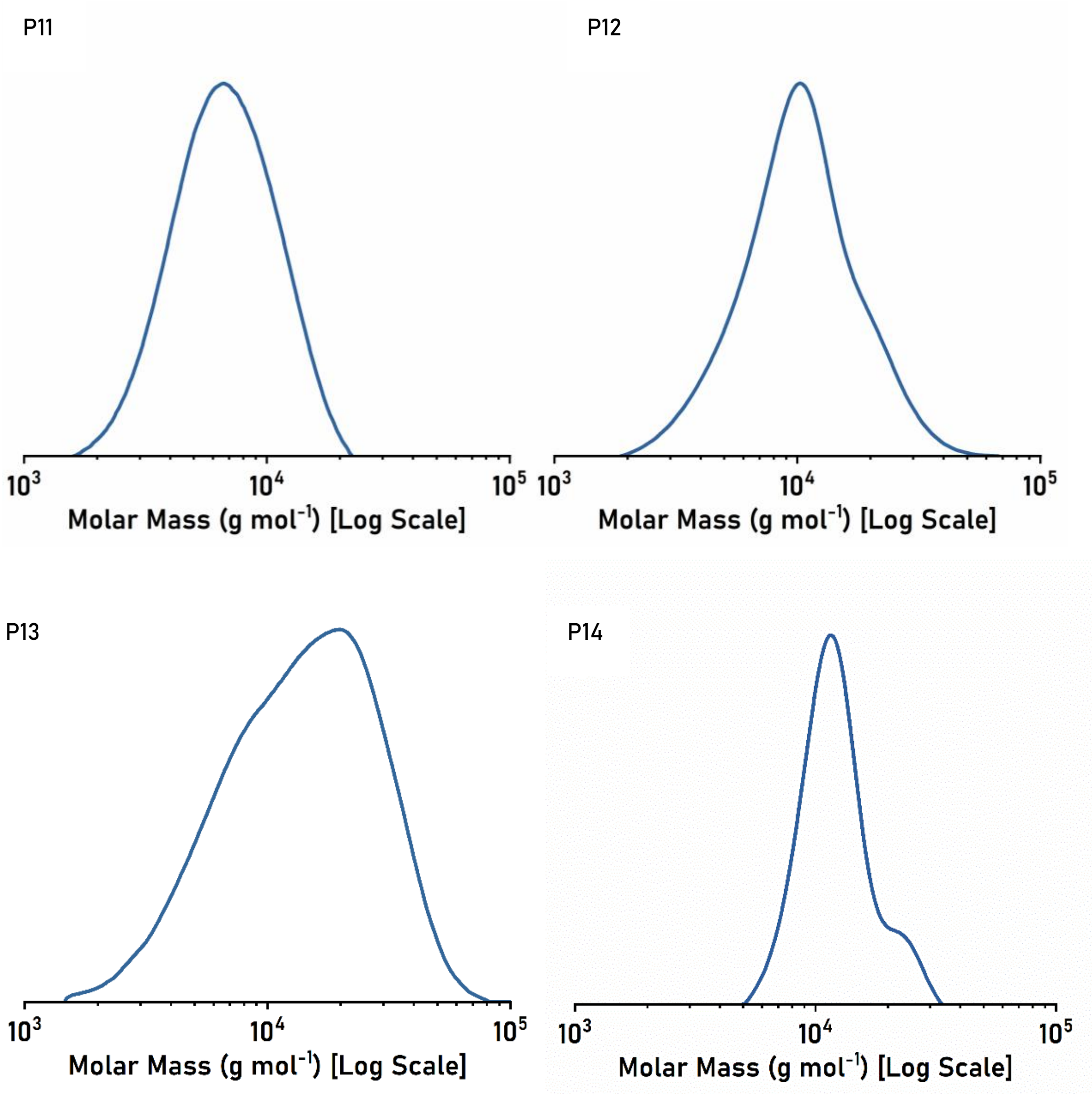

Figure S69: GPC chromatograms for polymers described in Table 1, **P11-14,** read left-to-right, top-to-bottom.

4.3 Kinetic Data for Polymers Described in Table 1

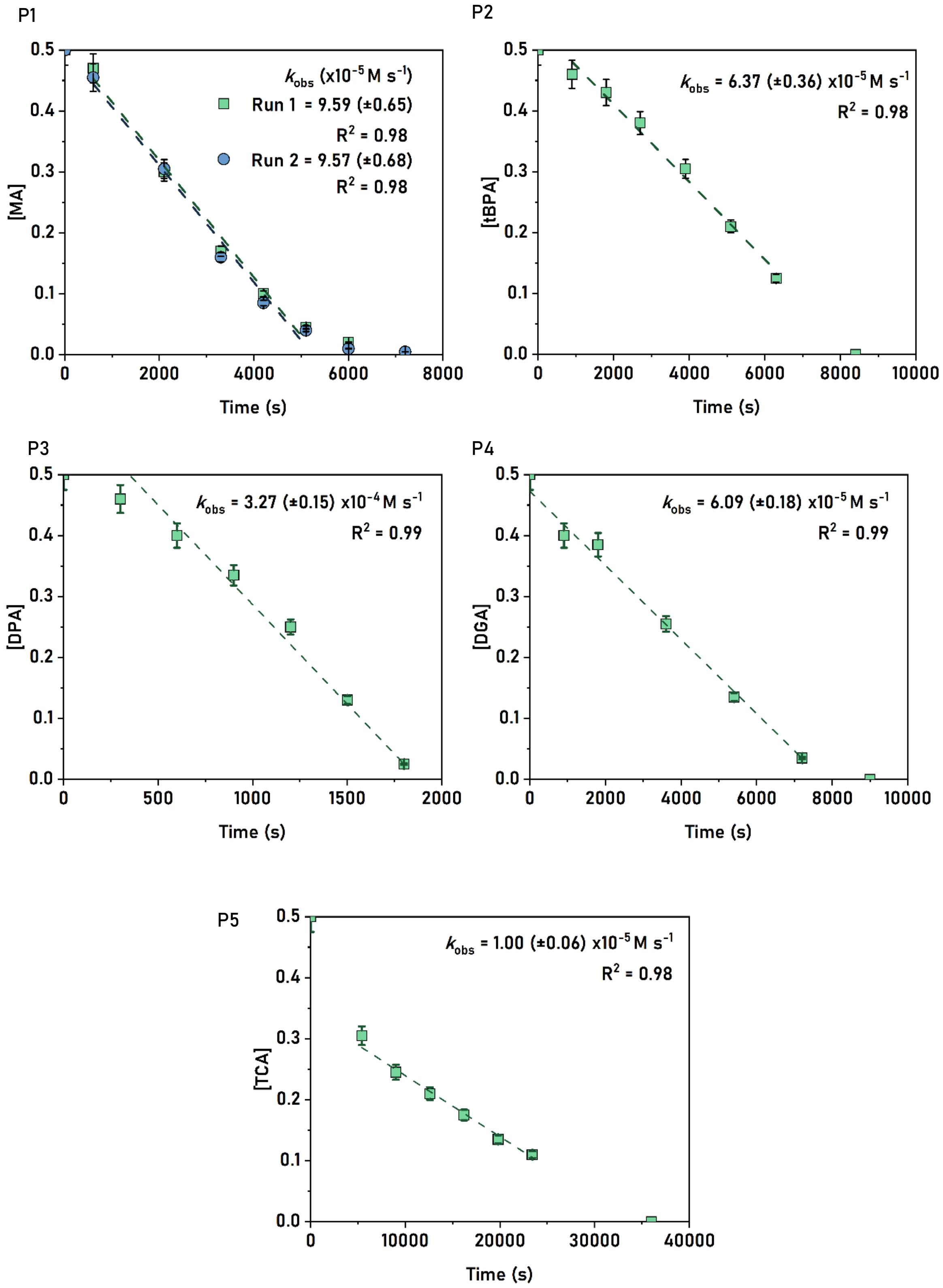

Figure S70: Plots of anhydride concentration vs. time, with linear fits to the data. The order in anhydride concentration was determined from the gradients of the linear fit. In these experiments [Anhydride] = 0.5 M. This diagram applies to the polymers described in Table 1, **P1-5**, read left-to-right, top-to-bottom.

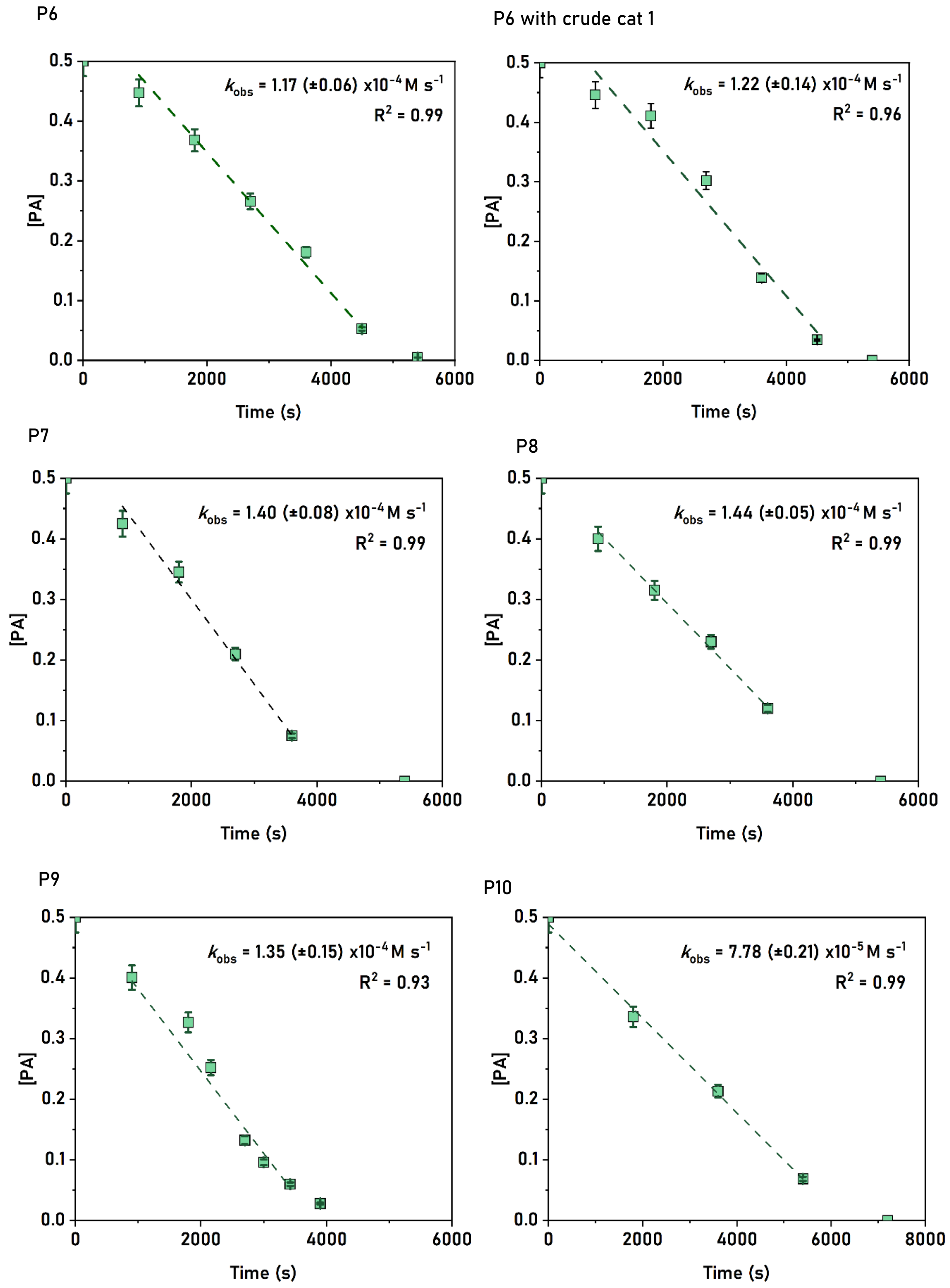

Figure S71: Plots of anhydride concentration vs. time, with linear fits to the data. The order in anhydride concentration was determined from the gradients of the linear fit. In these experiments [Anhydride] = 0.5 M. This diagram applies to the polymers described in Table 1, **P6-10**, read left-to-right, top-to-bottom.

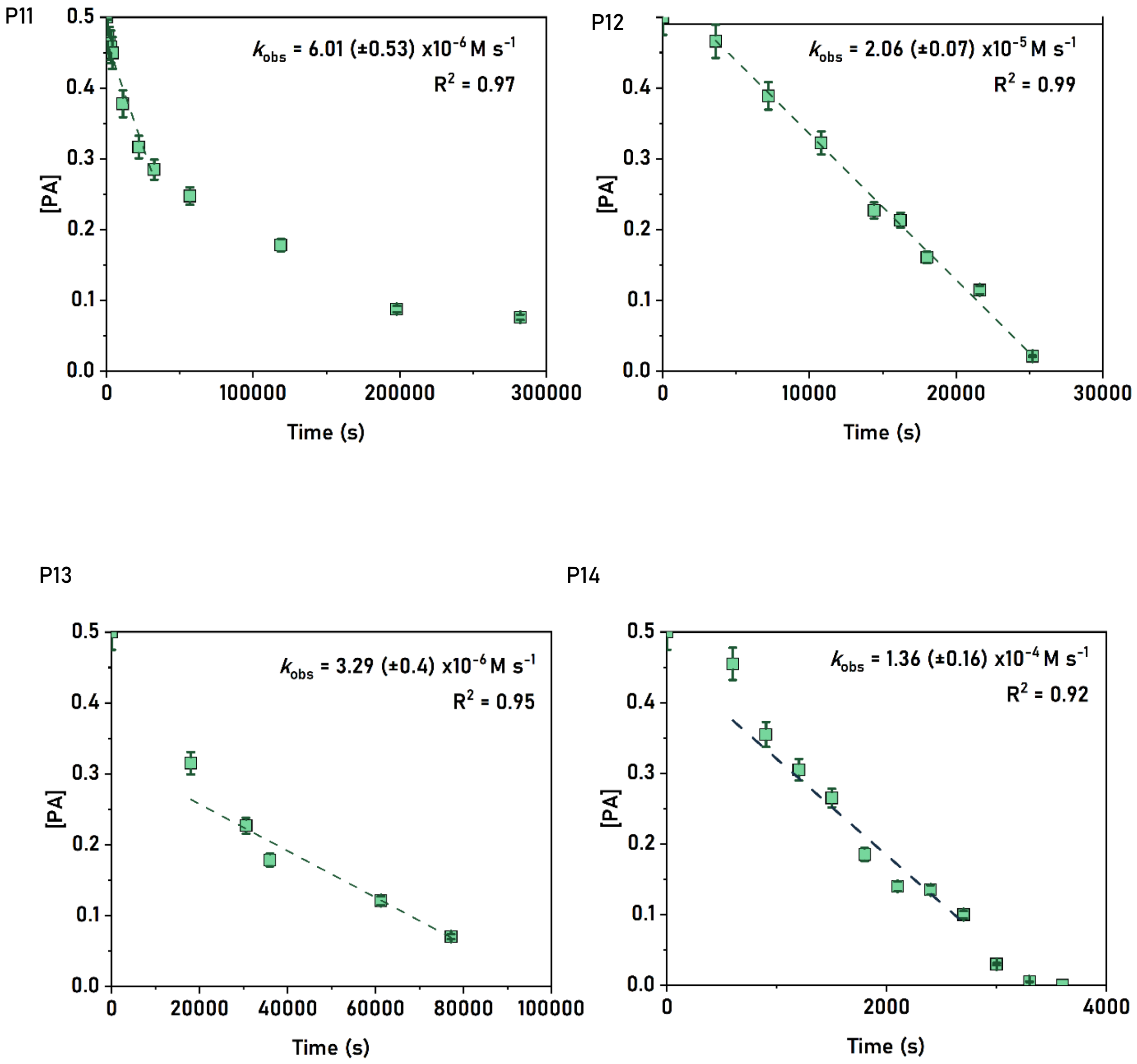

Figure S72: Plots of anhydride concentration vs. time, with linear fits to the data. The order in anhydride concentration was determined from the gradients of the linear fit. In these experiments [Anhydride] = 0.5 M. This diagram applies to the polymers described in Table 1, **P11-14**, read left-to-right, top-to-bottom.

4.4 DSC Data for Polymers Described in Table 1

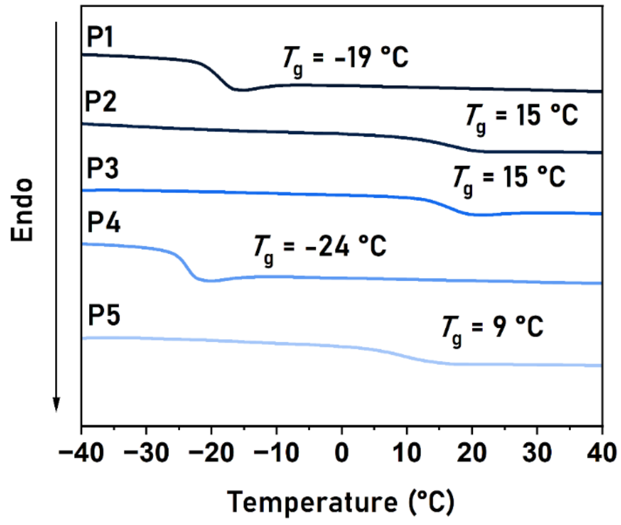

Figure S73: DSC Thermograms for Polymers **P1-5**. Data are presented at the glass transition temperatures

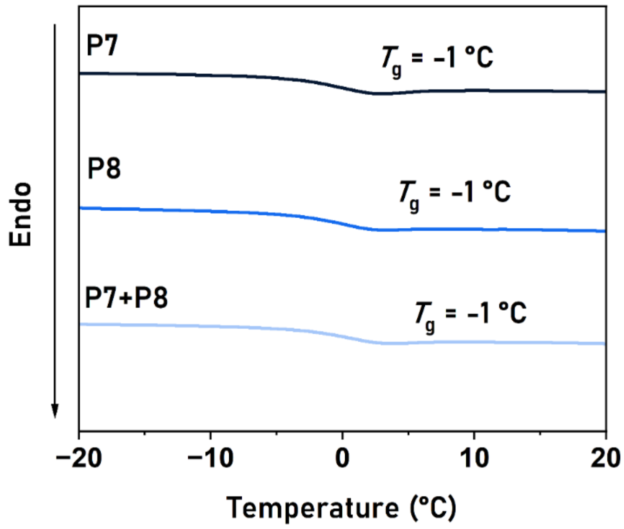

Figure S74: DSC Thermograms for Polymers **P7-8**. Data are presented at the glass transition temperatures

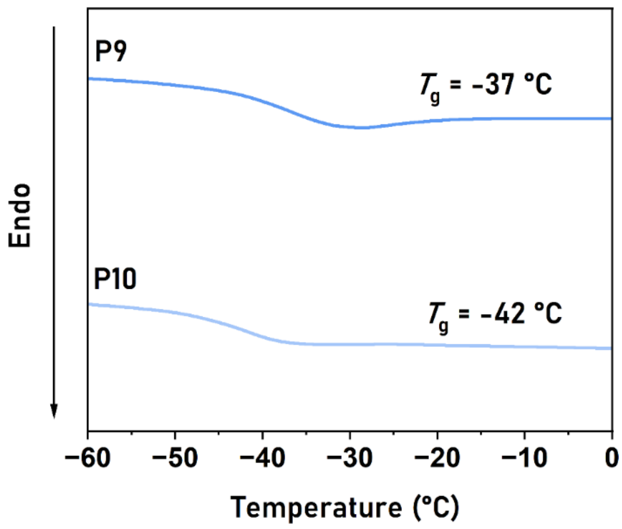

Figure S75: DSC Thermograms for Polymers **P9-10**. Data are presented at the glass transition temperatures

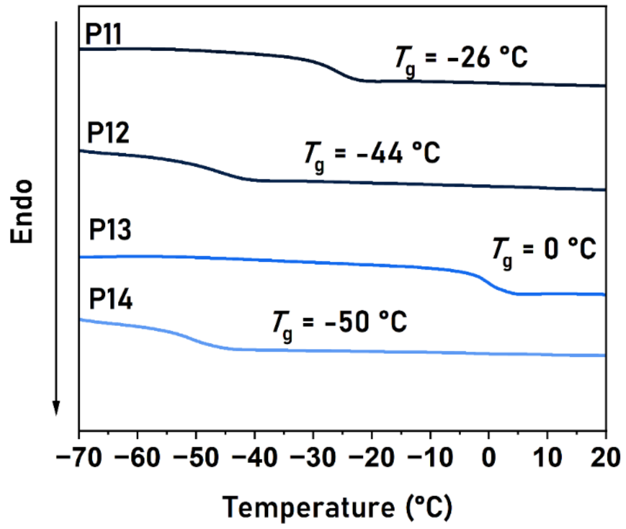

Figure S76: DSC Thermograms for Polymers **P11-14**. Data are presented at the glass transition temperatures

4.5 TGA Data for Polymers Described in Table 1

Table S2: TGA Data for **P1-14**

Polymer (#)	Anhyd.	Epoxide	$T_{d,5}$ (°C)^{g}	$T_{d,95}$ (°C)^{g}	*Notes*
**P1**	MA	BO	306	486	
**P2**	tBPA	BO	311	387	
**P3**	DPA	BO	327	463	
**P4**	DGA	BO	270	362	
**P5**	TCA	BO	273	>600	6% Mass remaining at 600 °C
**P6**[1]	PA	BO	318	379	Ref. 1
**P7**	PA	(*R*)-BO	304	367	
**P8**	PA	(*S*)-BO	314	387	
**P9**	PA	EDD	317	391	
**P10**	PA	ED	308	485	
**P11**	PA	AGE	294	>600	6% Mass remaining at 600 °C
**P12**	PA	EHGE	310	396	
**P13**	PA	BGE	283	450	
**P14**	MA	ED	344	576	

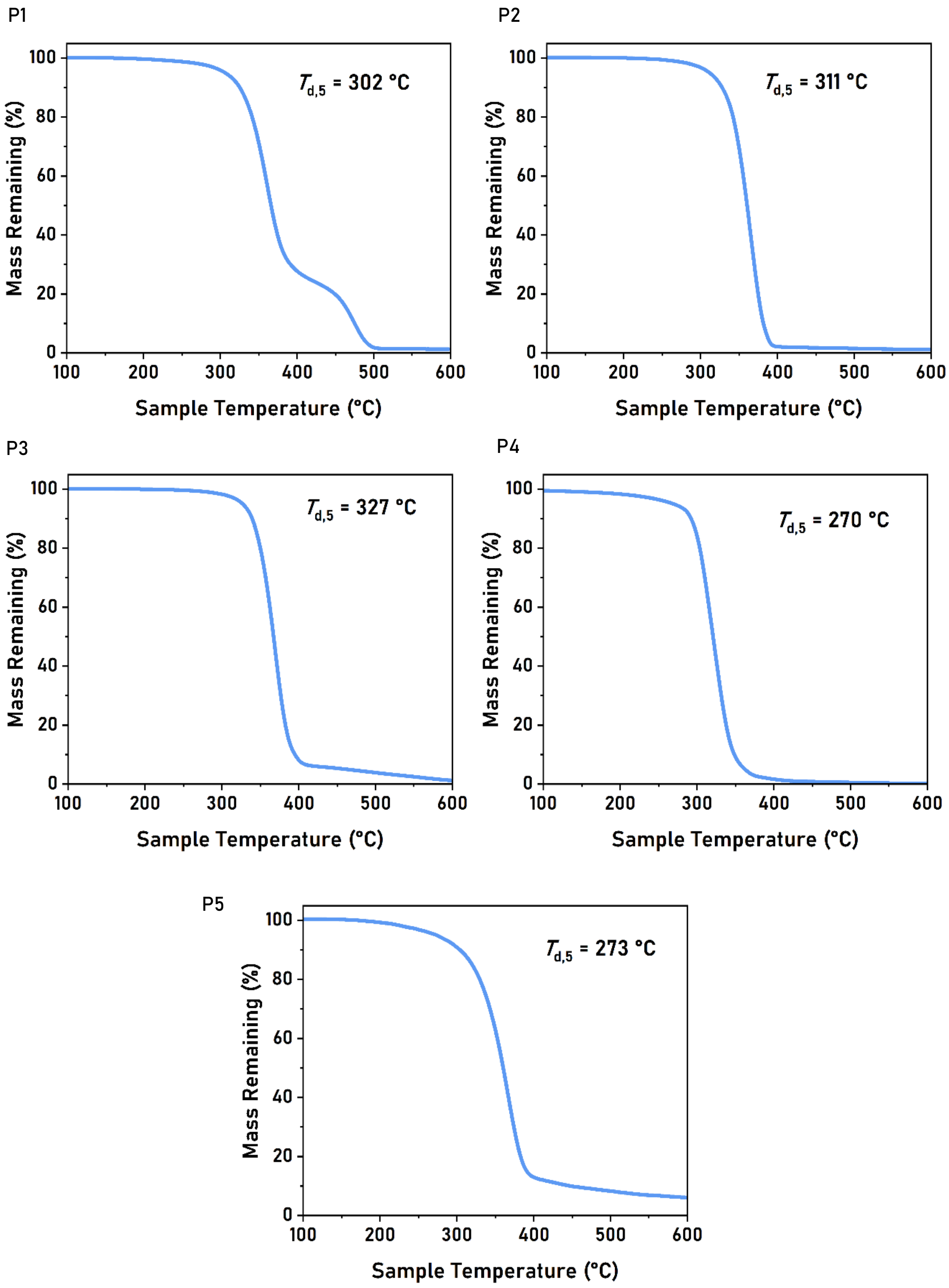

Figure S77: TGA data for **P1-5**, read left-to-right, top-to-bottom.

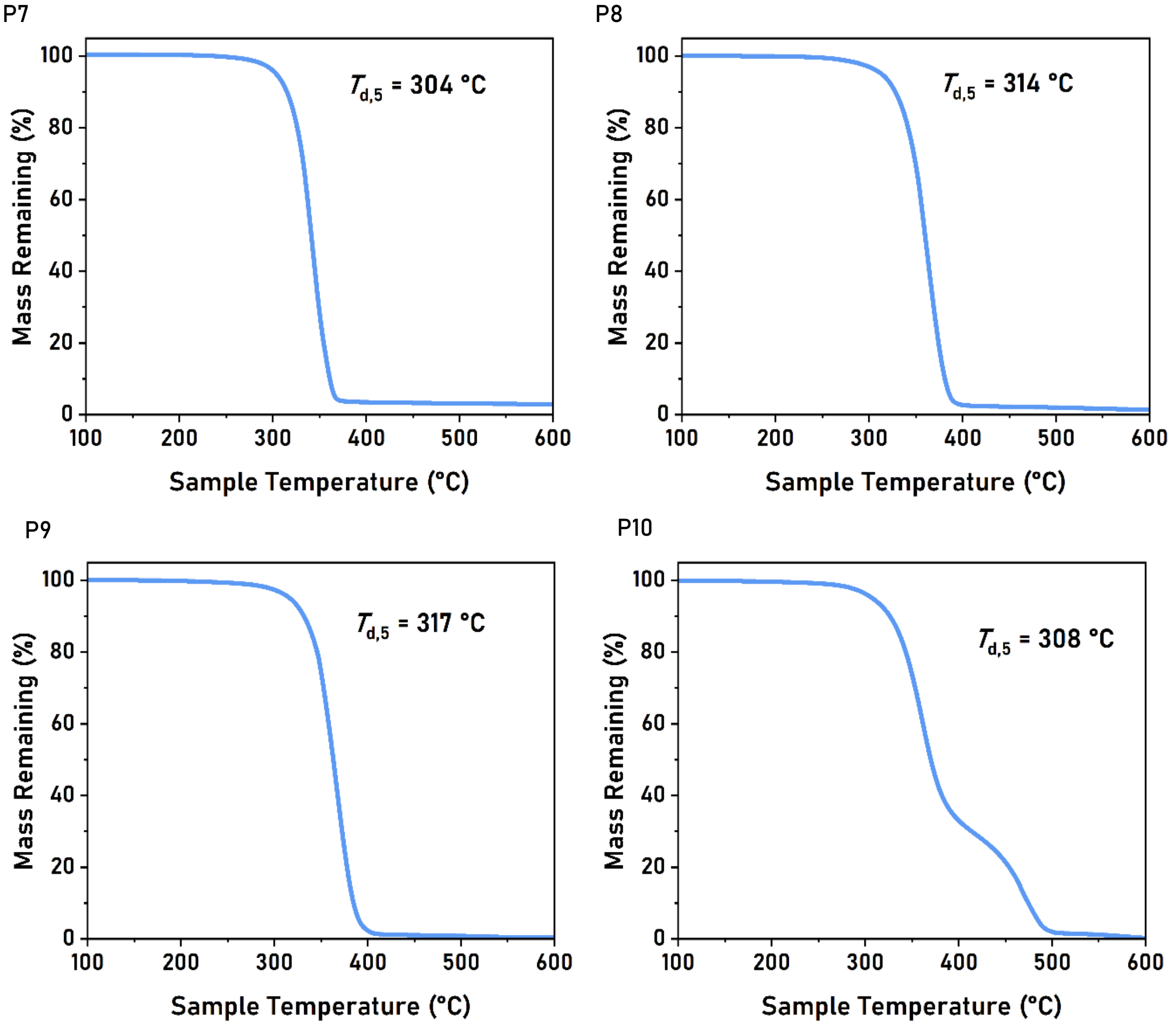

Figure S78: TGA data for **P7**-**10**, read left-to-right, top-to-bottom.

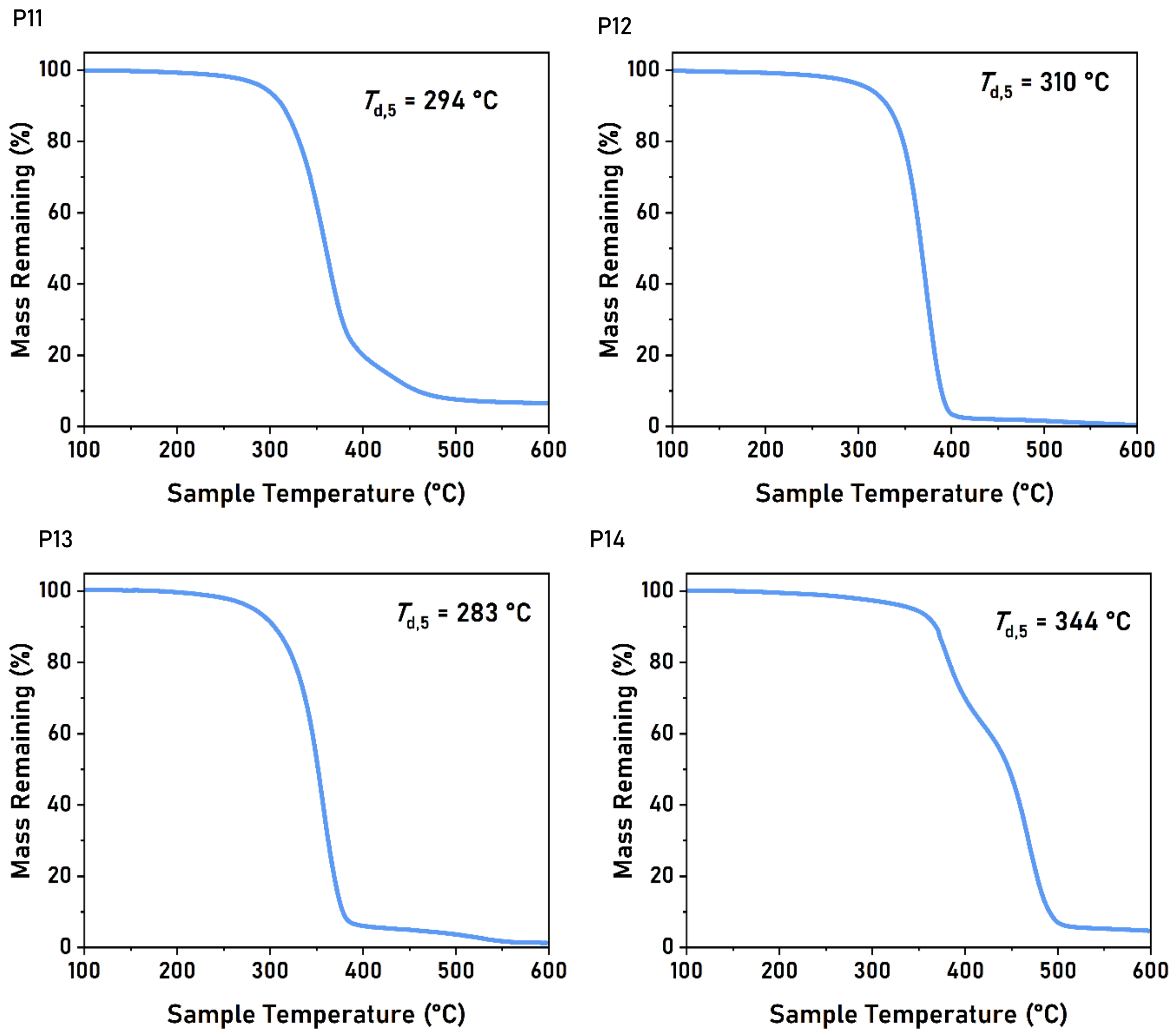

Figure S79: TGA data for **P11**-**14**, read left-to-right, top-to-bottom.

4.6 Data for P1’

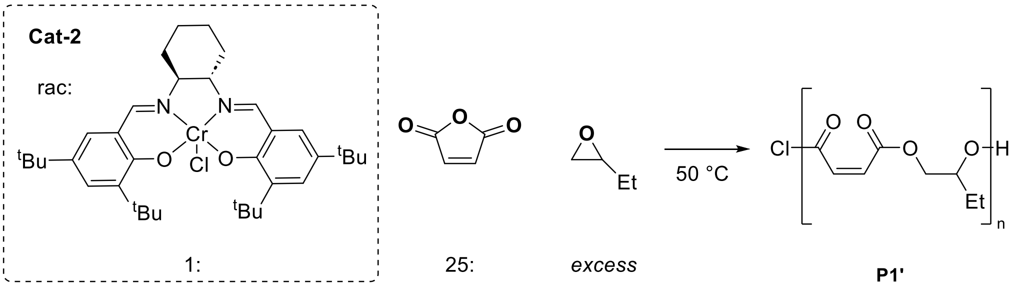

In a glovebox, Cr(III) catalyst **2** (6 mg, 0.01 mol) was weighed into a vial, then dissolved/suspended in BO (1 mL). Maleic anhydride (25 mg, 0.25 mmol) was added to the reaction mixture, the vial was sealed with electric tape, then parafilm, removed from the glovebox and heated to 50 °C. After the desired time, samples were cooled to 0 °C, taken into the glovebox for removal of aliquots (20 µL) or exposed to air to quench and evaporated to dryness. The crude polymer was characterized at ~10 mg/mL in THF for GPC and ~10 mg/mL in $CDCl_3$ for ^{1}H NMR spectroscopy. The pure polymer was obtained by precipitation from CH_2Cl_2/MeOH, followed by drying under vacuum, at 60 °C, overnight.

P1' - P(MA,BO)

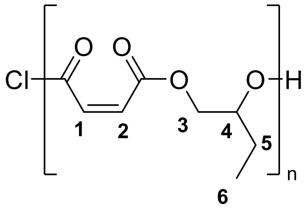

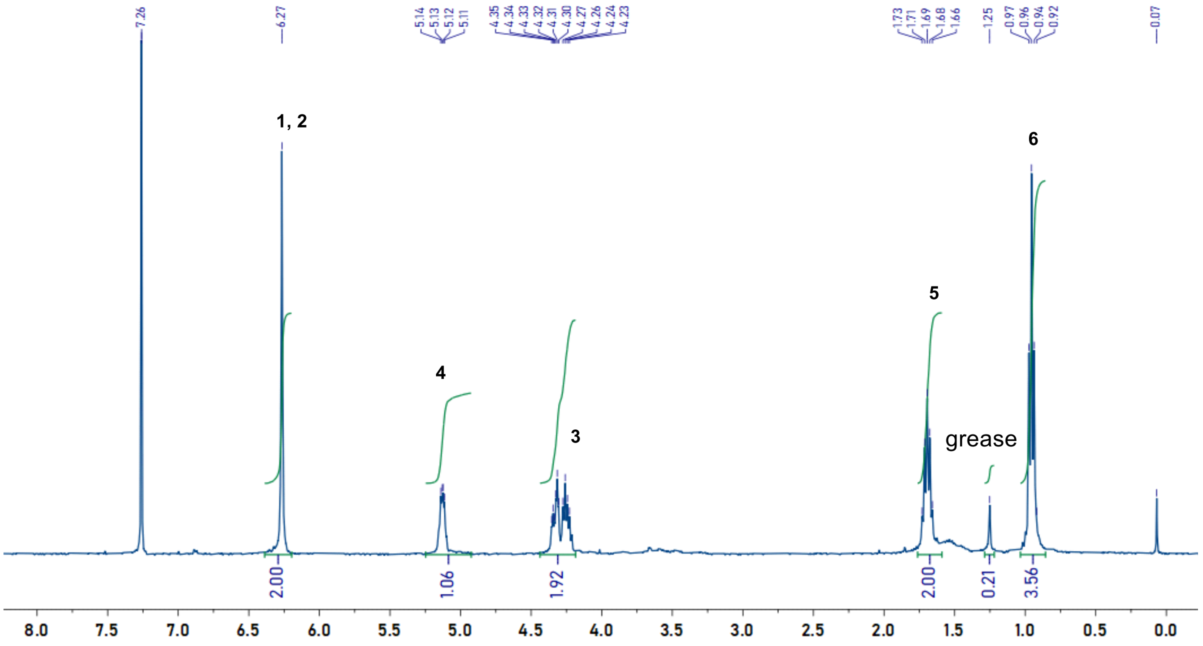

Figure S80: ^{1}H NMR spectrum from the reaction of [Cat]:[MA]:[BO] = 1: 25: 1150, (work up MeOH/ CH_2Cl_2). Spectrum corresponds to Table 1, **P1’** (400 MHz, $CDCl_3$).

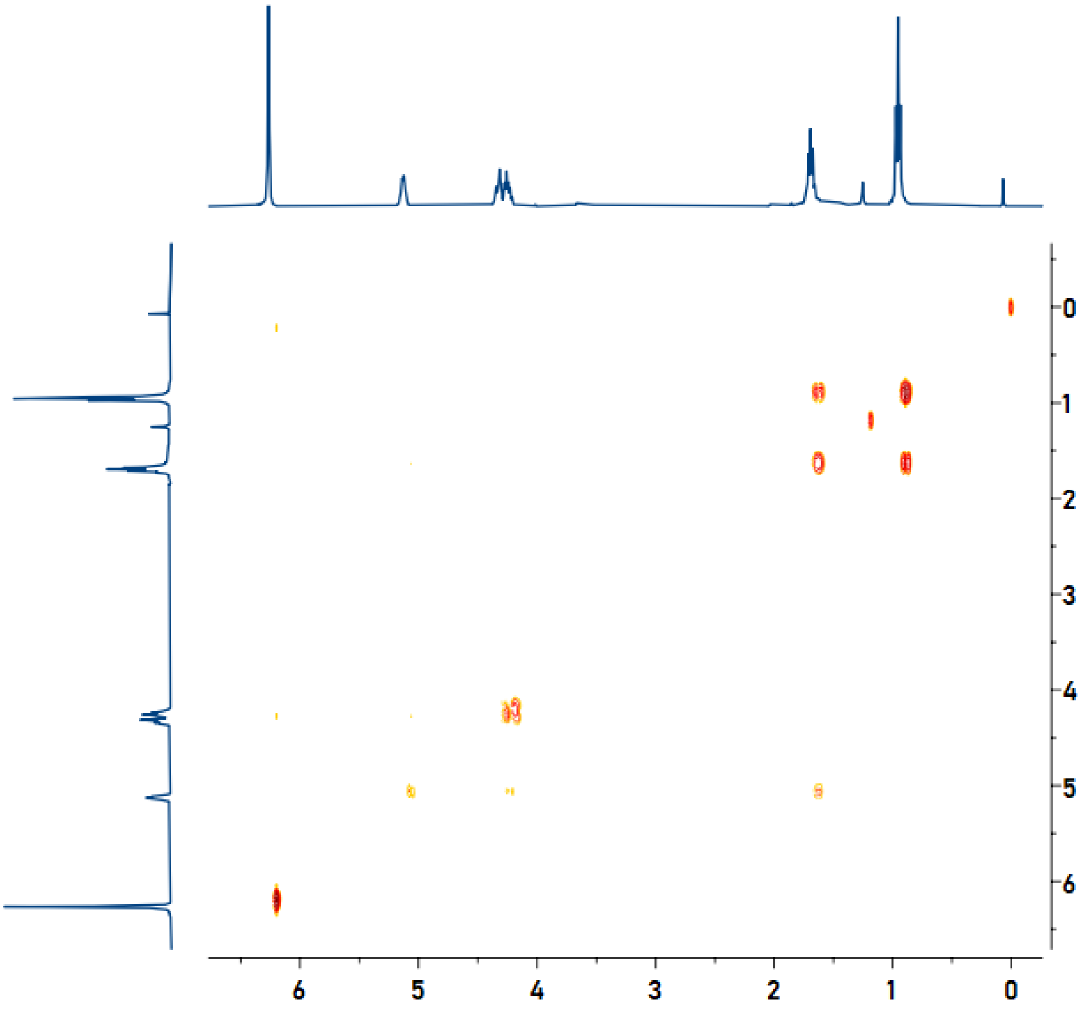

Figure S81: ^{1}H COSY NMR spectrum from the reaction of [Cat]:[MA]:[BO] = 1: 25: 1150, Table 1, **P1’** (400 MHz, $CDCl_3$).

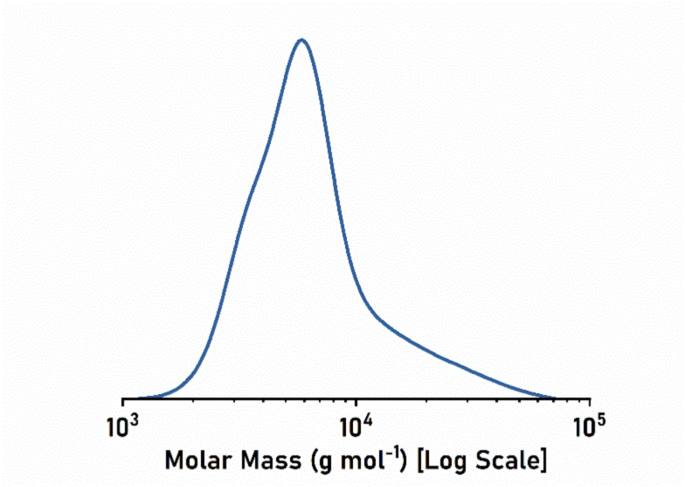

Figure S82: GPC chromatograms for P1’, described in main manuscript.

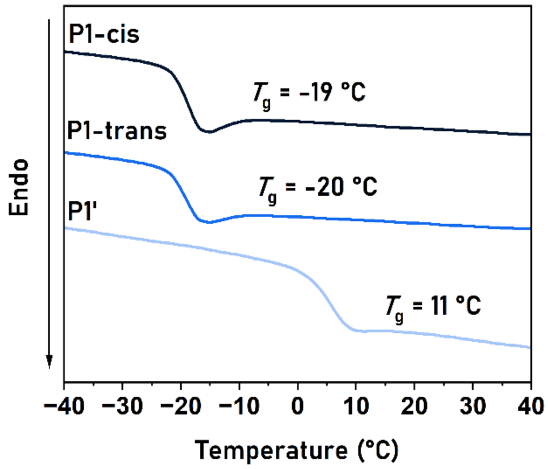

Figure S83: DSC Thermograms for Polymers **P1-cis, P1-trans and P1’**. Data are presented at the glass transition temperatures.

4.7 MALDI-ToF Data for SP1

4.7.1 Synthesis of SP1

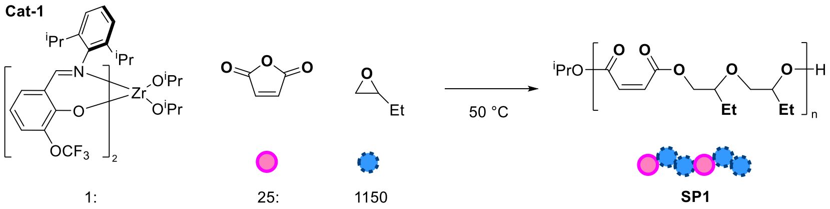

Scheme S16: Synthesis of **SP1** from **1**, MA and BO.

In a glovebox, catalyst **1** (18.8 mg, 0.02 mol) was weighed into a vial, then dissolved in 1 mL of butylene oxide. Maleic anhydride (49 mg, 0.5 mmol) was added to the reaction mixture, the vial sealed with electric tape, then parafilm, removed from the glovebox and heated to 50 °C. After 1 h, the sample was cooled to 0 °C, and exposed to air to quench and evaporated to dryness. The crude polymer was characterized at ~10 mg mL^{-1} ($CDCl_3$ solution) for ^{1}H NMR spectroscopy, showing complete anhydride conversion. The pure polymer was obtained by precipitation from DCM solutions, using methanol as the non-solvent, after drying under vacuum, at 60 °C, overnight. The polymer was prepared for MALDI-ToF analysis by pre-mixing **SP1** (10 mg mL^{-1} in THF), dithranol (10 mg mL^{-1} in THF), and NaOTf (10 mg mL^{-1} in MeOH) in 1:4:1 ratio and spotted onto a metal plate and allowed to fully evaporate before analysis; DP [MA]:[BO] = 13: 29, ABB selectivity = 95%, $M_{n,gpc}$ = 4.4 kg mol^{-1} (Đ = 1.17), $M_{n,theo}$= 3.4 kg mol^{-1} and $M_{n,nmr}$ = 3.1 kg mol^{-1}.

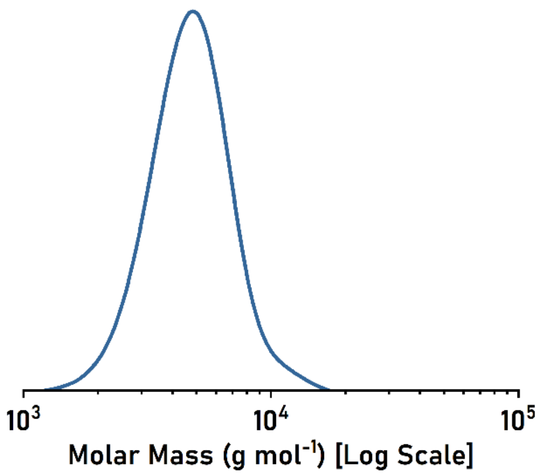

Figure S84: GPC chromatogram for polymers described in scheme S16, **SP1**.

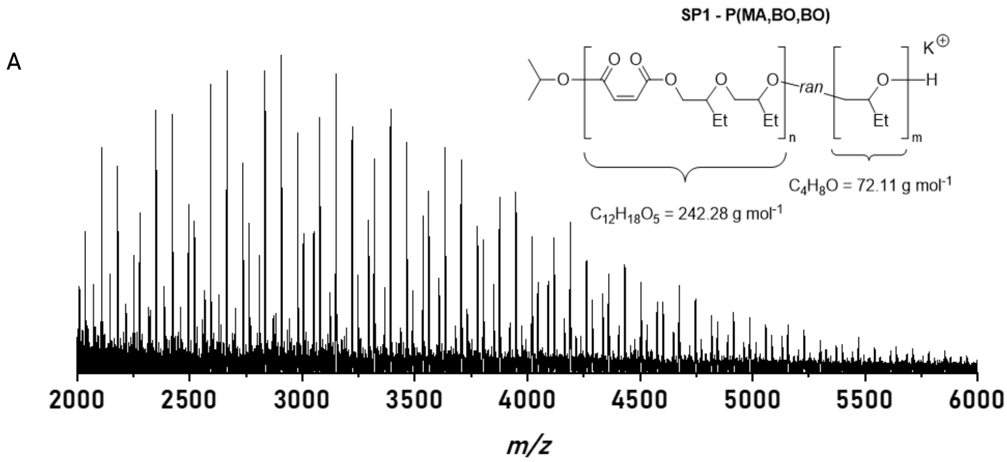

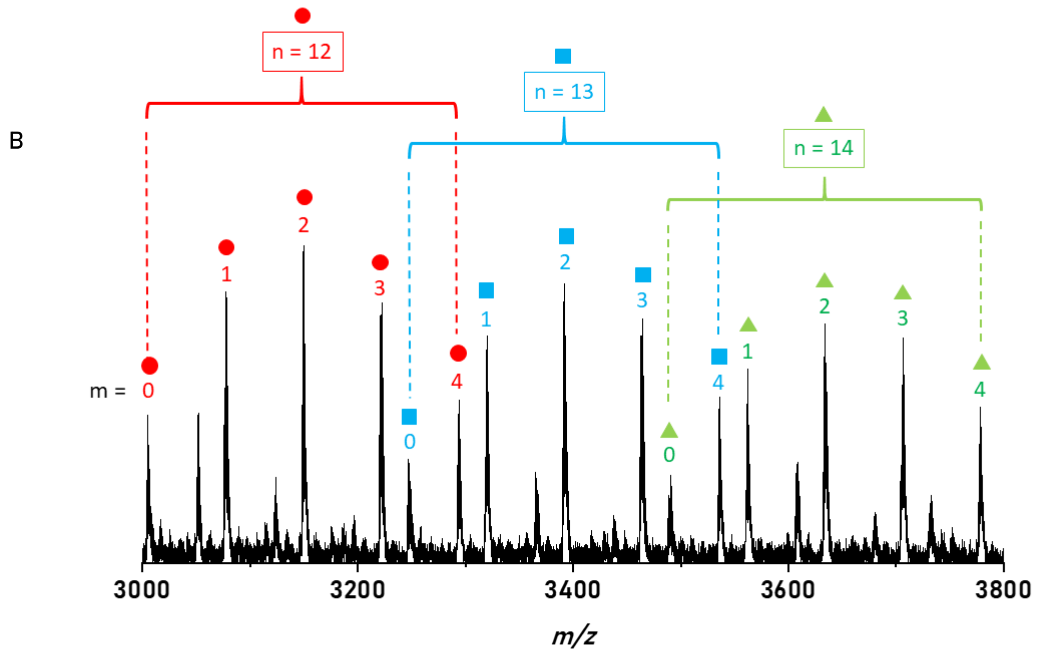

Figure S85: A) Full MALDI-ToF of **SP1** obtained with **1**. Conditions: [**1**] = 0.1 mM, [Anhydride] = 5 mM, Epoxide = 1 mL, 50 °C. B) Zoomed MALDI-ToF of **SP1** at DP of anhydride = 12-14 where end groups = ^{i}PrOH + K^+ = 99.02 g mol^{-1}, n = 242.12 g mol^{-1} and m = 72.11 g mol^{-1}. Polymers are separated by DP of anhydride (n) where n = 12 (red circle), n = 13 (blue square) and n = 14 (green triangle) and additional random epoxide enchainment's (m) where m = 0-4. Individual assignments can be found in Table S3.

Table S3: Selected assignments of analytes observed from the MALDI-ToF spectrum of **SP1** in **Figure S85**.

DP of anhydride (n)	m = 0	m = 1	m = 2	m = 3	m = 4
8	2035.74	2107.768	2179.797	2251.834	2323.862
9	2277.768	2349.807	2423.9383	2493.899	2565.955
10	2519.839	2591.898	2663.944	2736.004	2808.067
11	2761.925	2834.012	2906.072	2978.128	3050.191
12	3004.071	3076.133	3148.195	3220.247	3292.293
13	3246.187	3318.243	3390.291	3462.336	3534.376
14	3488.274	3560.31	3632.342	3704.362	3776.382

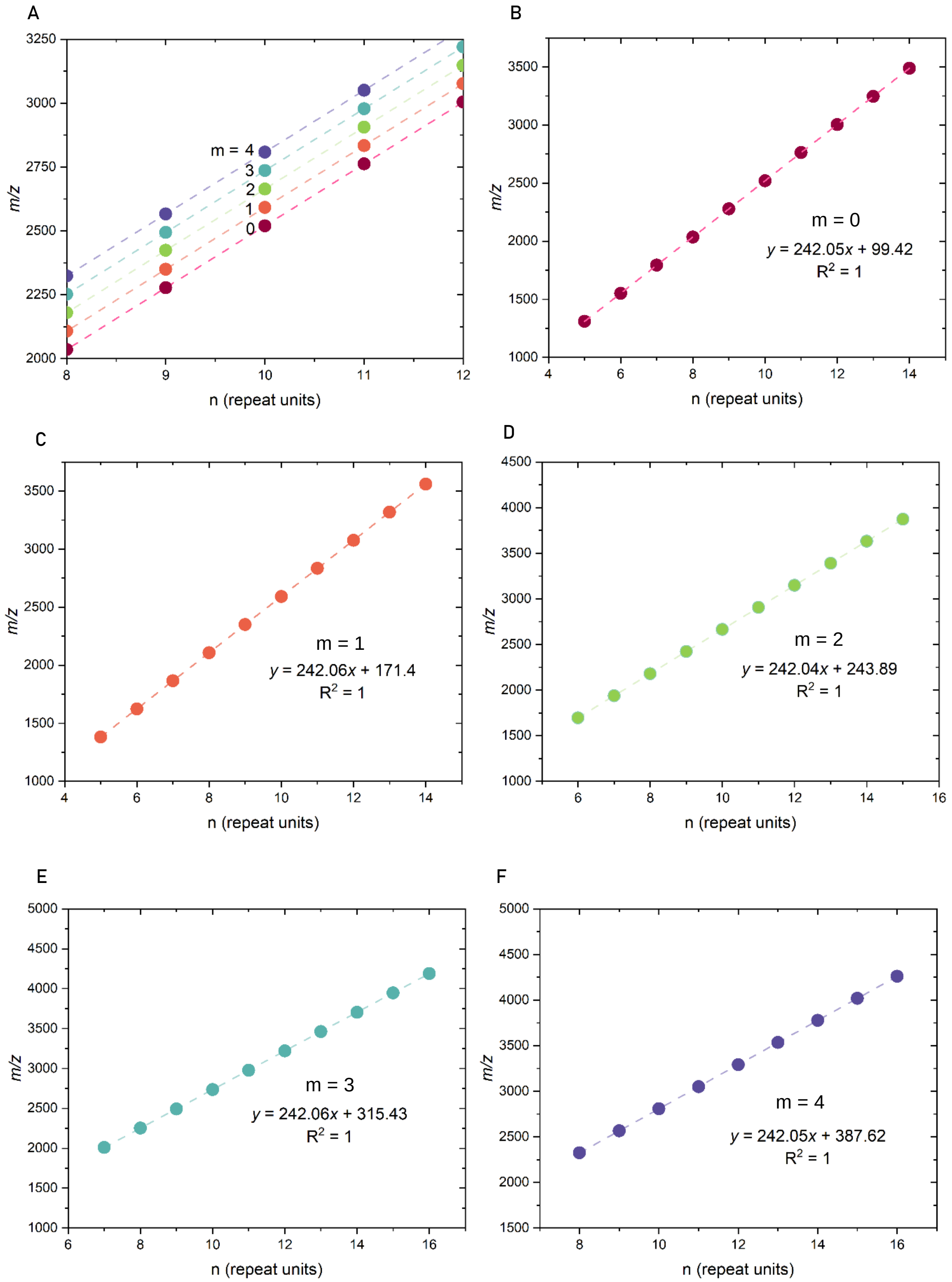

Figure S86: Plots of m/z values vs the number of MA-BO-BO repeat units (n) from the MALDI-TOF spectrum in Figure S85 separated by the number of additional BO units (m), where end groups = ^{i}PrOH + K^+ = 99.19 g mol^{-1}, n = 242.12 g mol^{-1} and m = 72.11 g mol^{-1}. A) Zoomed overlay of each m/z distribution of m; B) Plot of m/z values of MA-BO-BO repeats where m = 0. C) Plot of m/z values of MA-BO-BO repeats where m = 1. D) Plot of m/z values of MA-BO-BO repeats where m = 2. E) Plot of m/z values of MA-BO-BO repeats where m = 3. F) Plot of m/z values of MA-BO-BO repeats where m = 4.

4.8 Comparison of P6 Synthesized from Recrystallized and "Crude" Catalyst 1

4.8.1 Alternative Synthesis of 1

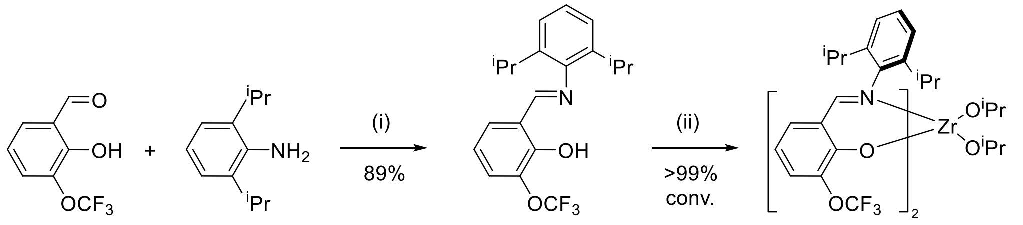

HL$_1$ **1**

Scheme S16: Modified synthesis of **1** (i) Formic acid (0.8 mol%), EtOH, 80°C, 18h. (ii) Toluene, 40°C, 24 h.

Using a modified literature procedure;[1] To a round bottom flask charged with 2-hydroxy-3-(trifluoromethoxy) benzaldehyde (2.54 g, 12.32 mmol), dissolved in ethanol (50 mL), was added diisopropylaniline (2.32 mL, 12.32 mmol) and formic acid (0.1 mmol). The reaction was heated to reflux for 18 h, cooled to RT and concentrated to a yellow oil. Proligand HL$_1$ was obtained after column chromatography purification by the Biotage® Selekt™ [SNAP Ultra C18 120g, 100 mL min^{-1}, pentane: EtOAc (100:0 to 97:3 3 CV, 97:3 2 CV, 97:3 to 94: 6 2 CV)], the title compound **HL$_1$** (4.01 g, 10.97 mmol, 89%) as a fine yellow powder with spectroscopic data in accordance with the literature.[1]

Using a modified literature procedure;[1] To an ampoule charged with HL$_1$ (1 g, 2.73 mmol) dissolved in toluene (50 mL) was added [Zr(O^{i}Pr)$_4$(HO^{i}Pr)] (531 mg, 1.36 mmol) and stirred for 24 h, at 40 °C. The reaction mixture was dried under dynamic vacuum (1 x 10^{-3} mbar) overnight. The title compound **1** was obtained as pale-yellow powder in >99% conversion and was used directly as a catalyst without further purification to synthesise **SP2**. All spectroscopic data was in accordance with literature.[1]

Note: for all other general reactivity studies compound **1** was further purified by reported methods.[1] After drying under dynamic vacuum. **1** was recrystallized in toluene/hexane at –30 °C, overnight, to give the single X-ray quality crystals (> 49 % yield) to ensure reproducibility between batches. The overall catalyst yield over two steps is 89% or 44% depending on purification methods.

Table S4: Ring-opening copolymerisation (ROCOP) of PA and BO with catalyst **1.*[a]***

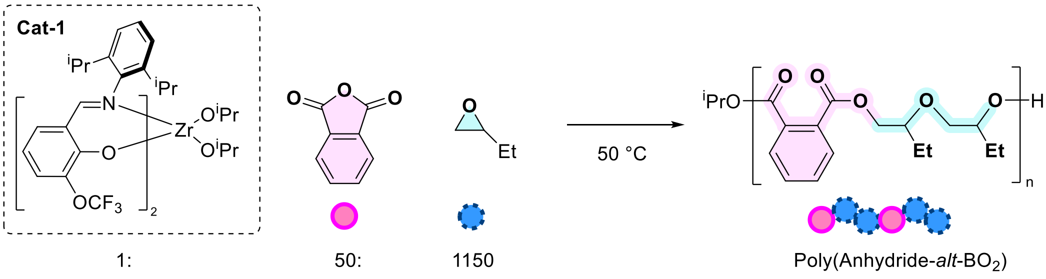

Polymer (#)	Degrees of Polymerization (DP): [PA]:[BO][b]	ABB Selectivity (%)[c]	M_n (Đ) [kg mol^{-1}][d]	M_n (Theo)[e]	k_{obs} (x10^{-5} M s^{-1})[f]
**P6 (Table 1)**	25: 57	96	6.7 (1.11)	7.9	11.7
**SP2**	25: 61	94	4.9 (1.25)	8.2	12.0

[a]ROCOP Conditions: [**1**] = 10 mM, [Anhydride] = 0.5 M, Epoxide = 1 mL, 50 °C, where **P6** was synthesised using a recrystallised catalyst **1** and **SP2** using a "crude" catalyst **1** (see SI Section 4.8). [b]DP of monomer measured by integration of the polymer in the 1H NMR spectra of crude polymers against 2 iso-propoxide initiators. [c]Determined as the theoretical percentage of perfect ABB epoxide selectivity (66.67%) against calculated epoxide selectivity (range = 67-70%) (Fig. S23). [d]Determined by gel permeation chromatography (GPC), using THF as the eluent, and calibrated using narrow MW polystyrene standards (Fig. S87). [e]Theoretical M_n are calculated from the monomer conversion data and assume both iso-propoxides initiate.

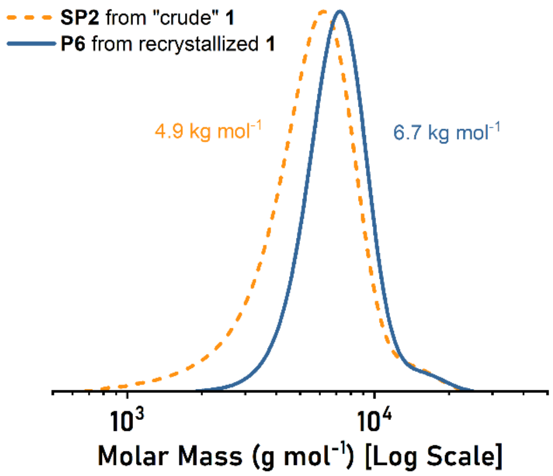

Figure S87: GPC chromatogram for polymers described in Table S4, **P6** and **SP2**.

4.9 Synthesis of Higher Weight P6

It was of interest to probe whether catalyst **1** was active at lower loadings and capable of synthesizing polymers with an increased molar mass, Table S5. Using the standard reaction conditions presented in table 1, the loading of catalyst to anhydride/epoxide was decreased by a factor of 5 and then by a factor 10 (**SP3** and **SP4** respectively). In all cases full conversion of anhydride was observed and the **ABB** selectivity remained high (97%) indicating the catalyst is tolerant at low loadings. It was observed that a high molecular weight shoulder was observed in both GPC chromatograms and the overall molar mass was below the expected theoretical value.

Table S5: Ring-opening copolymerisation (ROCOP) of PA and BO with catalyst **1*[a]***.

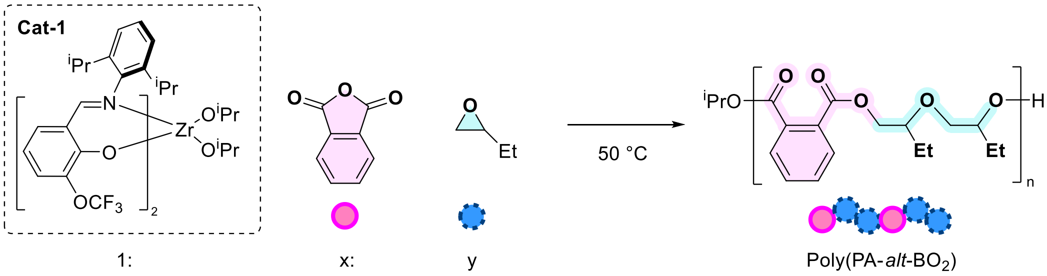

Polymer (#)	x	y	Time	Degrees of Polymerization (DP): [PA]:[BO][b]	ABB Selectivity (%)[c]	M_n (Đ) [kg mol^{-1}][d]	M_n (Theo)[e]
**SP3**	250	2925	18 h	250: 558	97	30.6 [1.18]	38.9
**SP4**	500	5557	48 h	500: 1113	97	45.6 [1.28]	77.2
**SP5**	500	2925	48 h	500: 1097	97	39.4 [1.22]	76.6

[a]ROCOP Conditions: [**1**] = 0.5-2 mM, [Anhydride] = 0.25-0.5 M, Epoxide = 0.5-1 mL, 50 °C. [b]DP of monomer measured by integration of the polymer in the ^1H NMR spectra of crude polymers against 2 iso-propoxide initiators. [c]Determined as the theoretical percentage of perfect ABB epoxide selectivity (66.67%) against calculated epoxide selectivity (range = 67-70%) (See Fig. S23 for method). [d]Determined by gel permeation chromatography (GPC), using THF as the eluent, and calibrated using narrow MW polystyrene standards (Fig. S88). [e]Theoretical M_n are calculated from the monomer conversion data and assume both iso-propoxides initiate.

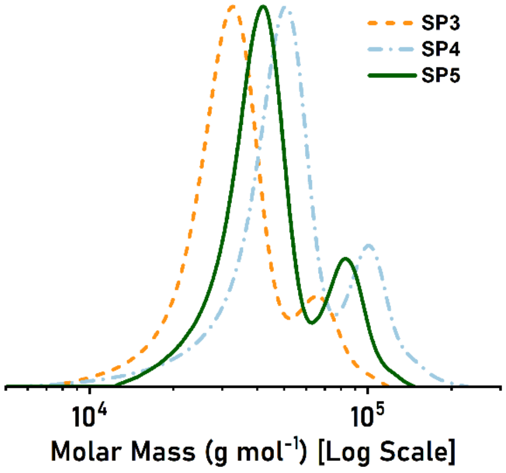

Figure S88: GPC chromatogram for polymers described in Table S5, **SP3-SP5**.

5.0 Characterization Data for Polymers Described in Scheme 2

5.1 NMR Spectra for Polymers Described in Scheme 2

5.1.1 NMR Spectra for P15

P15 - P(PA-BO-BO)-*ran*-P(PA,BO,DHF)

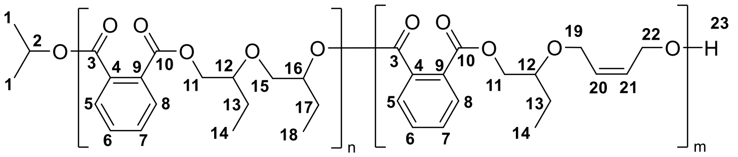

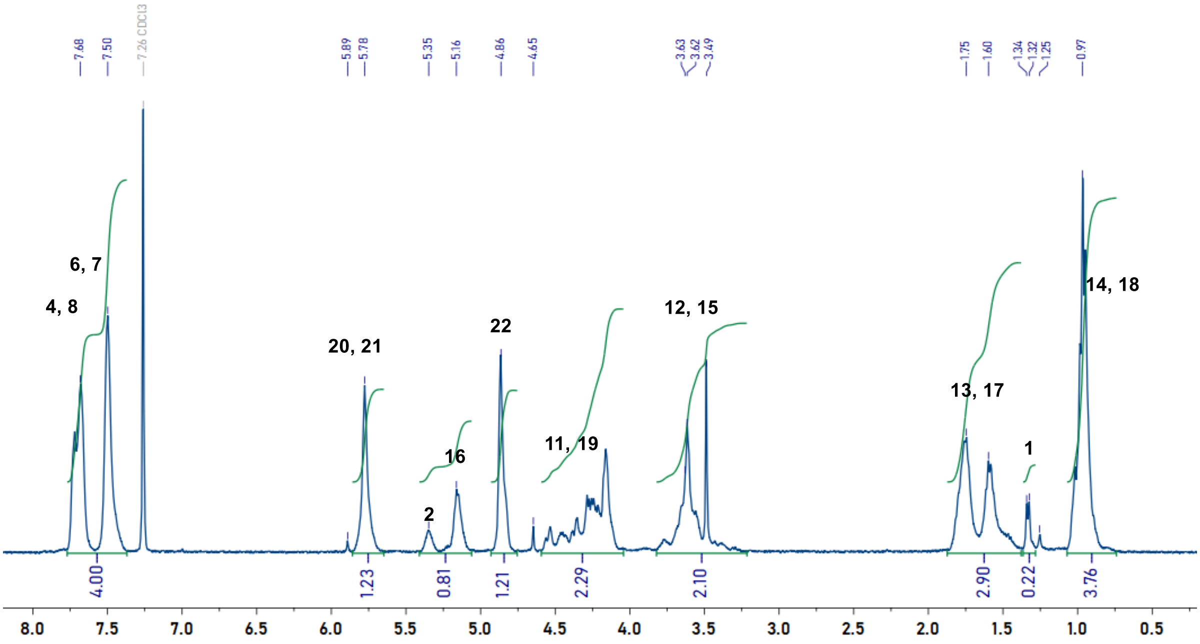

Figure S89: ^{1}H NMR spectrum from the reaction of [Cat]:[PA]:[BO]:[DHF] = 1: 50: 228: 1154, (work-up in MeOH/ CH_2Cl_2). Spectrum corresponds to Scheme 2, **P15** (400 MHz, $CDCl_3$).

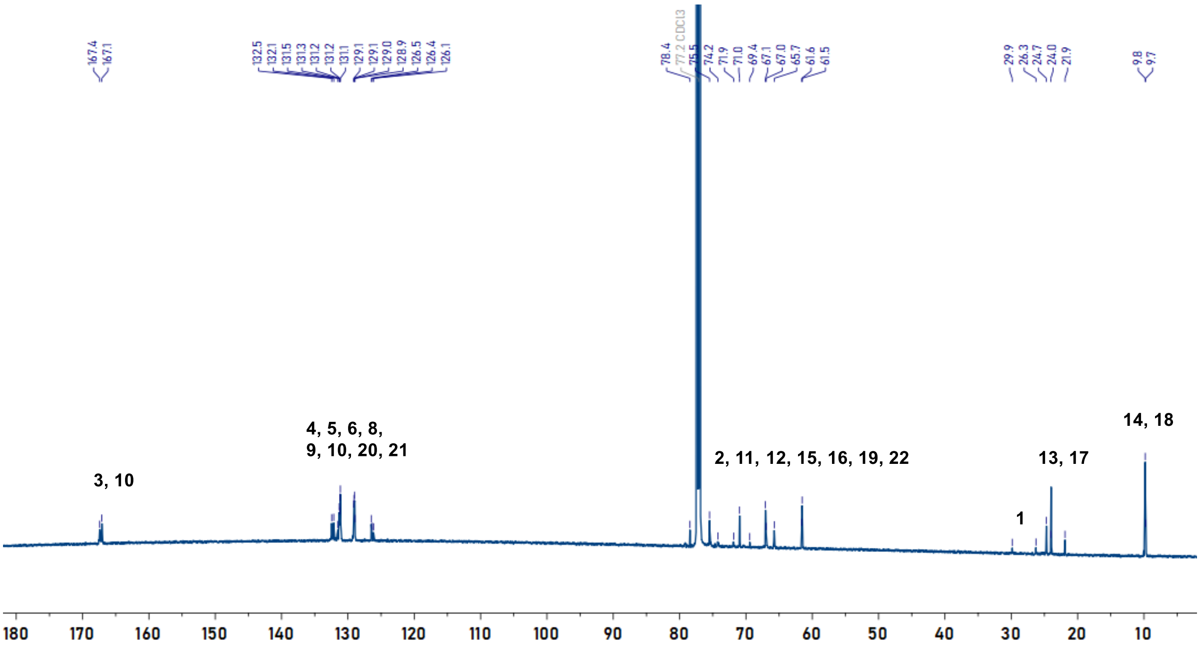

Figure S90: $^{13}C\{^{1}H\}$NMR spectrum from the reaction of [Cat]:[PA]:[BO]:[DHF] = 1: 50: 228: 1154, after work-up in MeOH/DCM. Spectrum corresponds to Scheme 2, **P15** (151 MHz, $CDCl_3$).

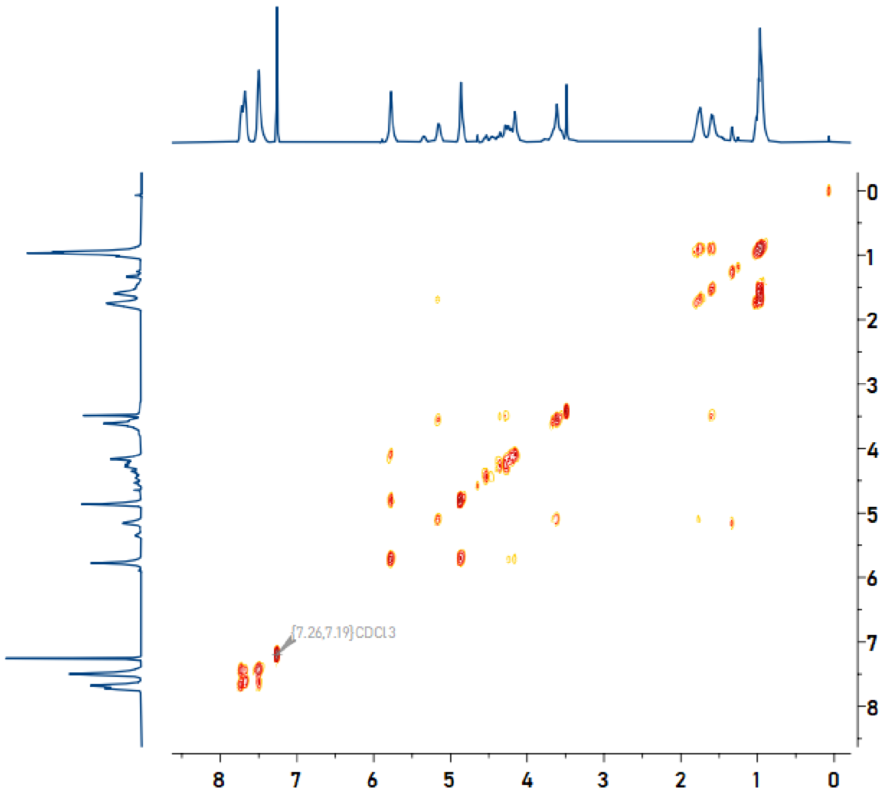

Figure S91: ^{1}H COSY NMR spectrum from the reaction of [Cat]:[PA]:[BO]:[DHF] = 1: 50: 228: 1154, Scheme 2, **P15** (400 MHz, $CDCl_3$).

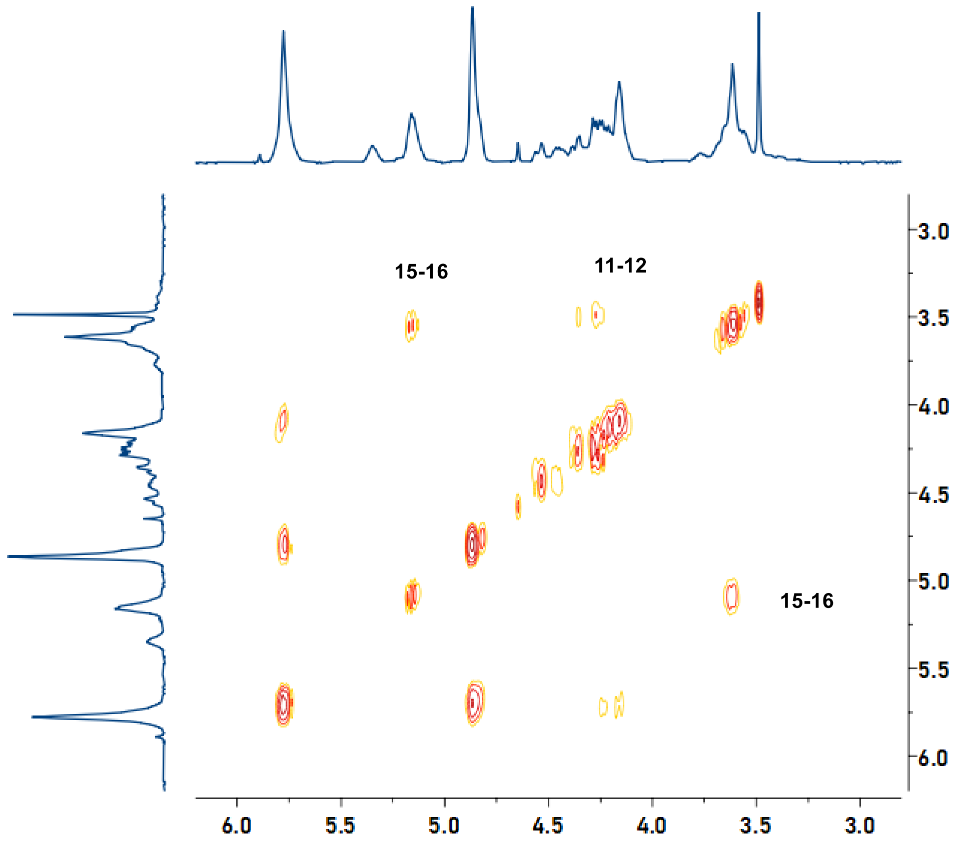

Figure S92: Magnified ^{1}H COSY NMR spectrum from the reaction of [Cat]:[PA]:[BO]:[DHF] = 1: 50: 228: 1154, Scheme 2, **P15** (400 MHz, $CDCl_3$).

5.1.2 NMR Spectra for P16

P16 - P(PA-BO-BO)-*ran*-P(PA,BO,OBH)

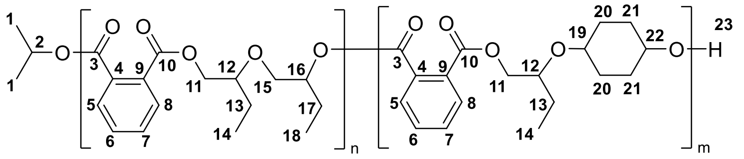

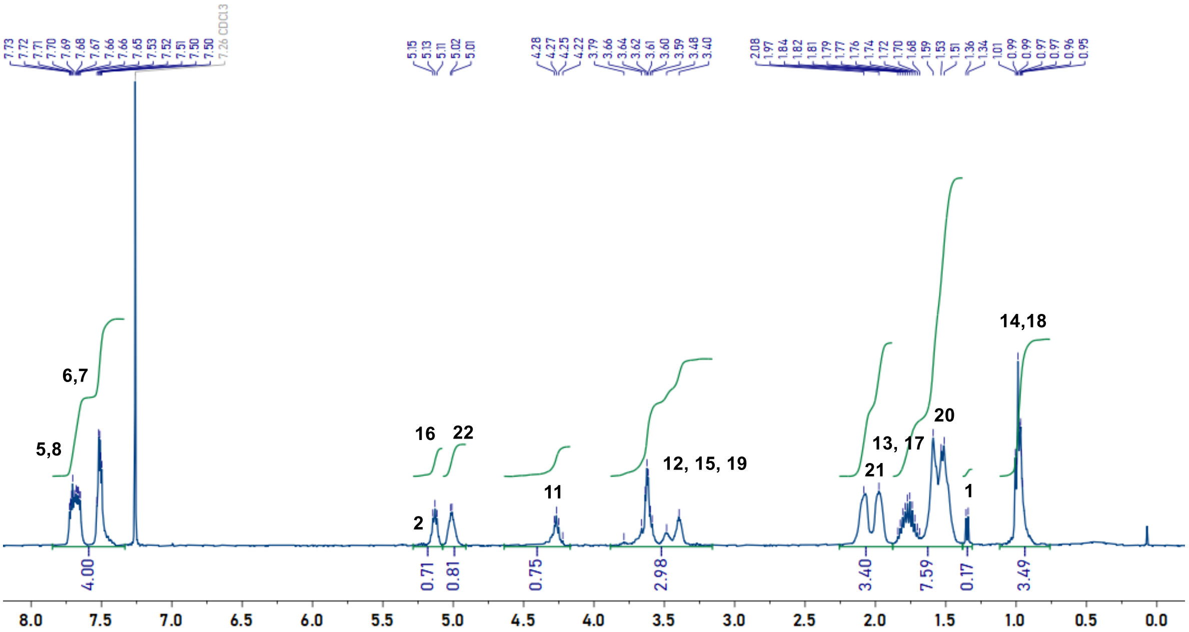

Figure S93: ^{1}H NMR spectrum from the reaction of [Cat]:[PA]:[BO]:[OBH] = 1: 50: 228: 986, (work-up in MeOH/ CH_2Cl_2). Spectrum corresponds to Scheme 2, **P16** (400 MHz, $CDCl_3$).

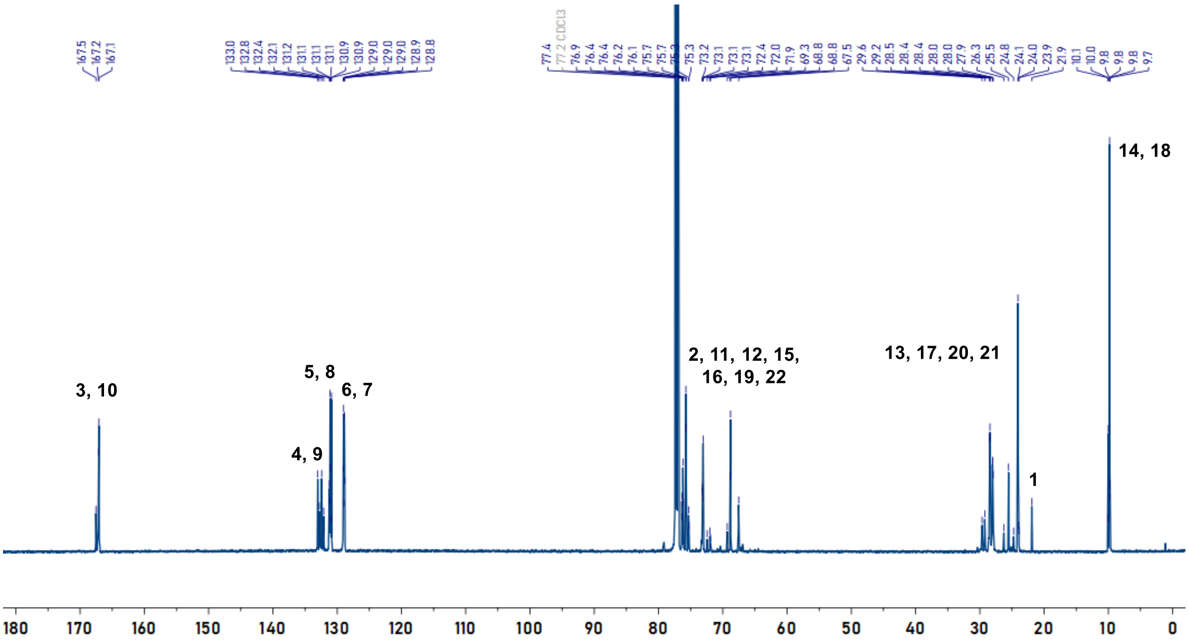

Figure S94: $^{13}C\{^1H\}$ NMR spectrum from the reaction of [Cat]:[PA]:[BO]:[OBH] = 1: 50: 228: 986, Scheme 2, **P16** (151 MHz, $CDCl_3$).

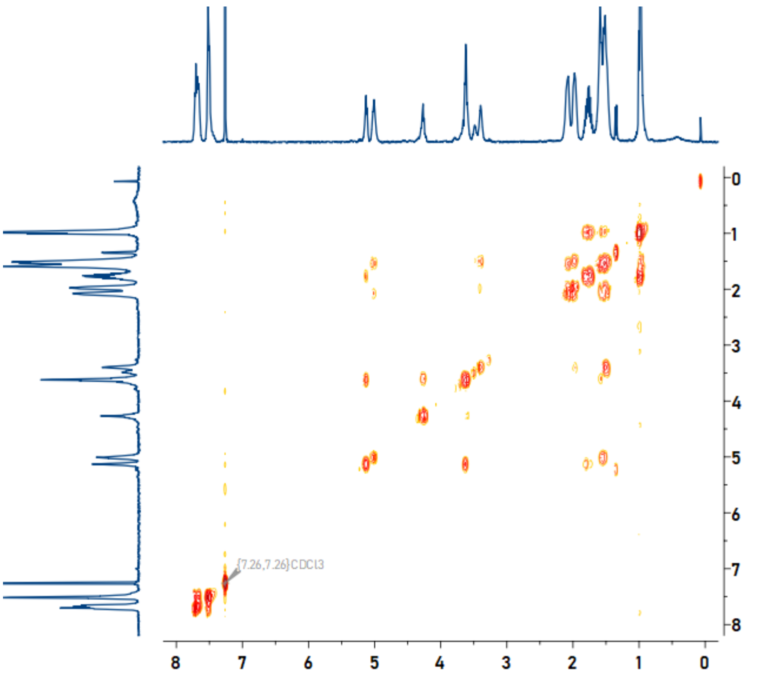

Figure S95: ^{1}H COSY NMR spectrum from the reaction of [Cat]:[PA]:[BO]:[OBH] = 1: 50: 228: 986, Scheme 2, **P16** (400 MHz, $CDCl_3$).

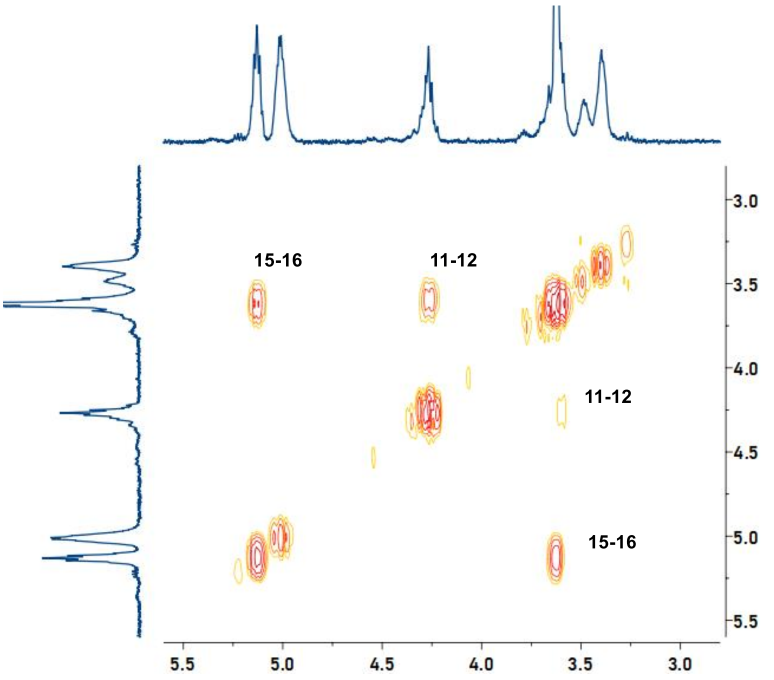

Figure S96: Magnified ^{1}H COSY NMR spectrum from the reaction of [Cat]:[PA]:[BO]:[OBH] = 1: 50: 228: 986, Scheme 2, **P16** (400 MHz, $CDCl_3$).

5.2 GPC Data for Polymers Described in Scheme 2

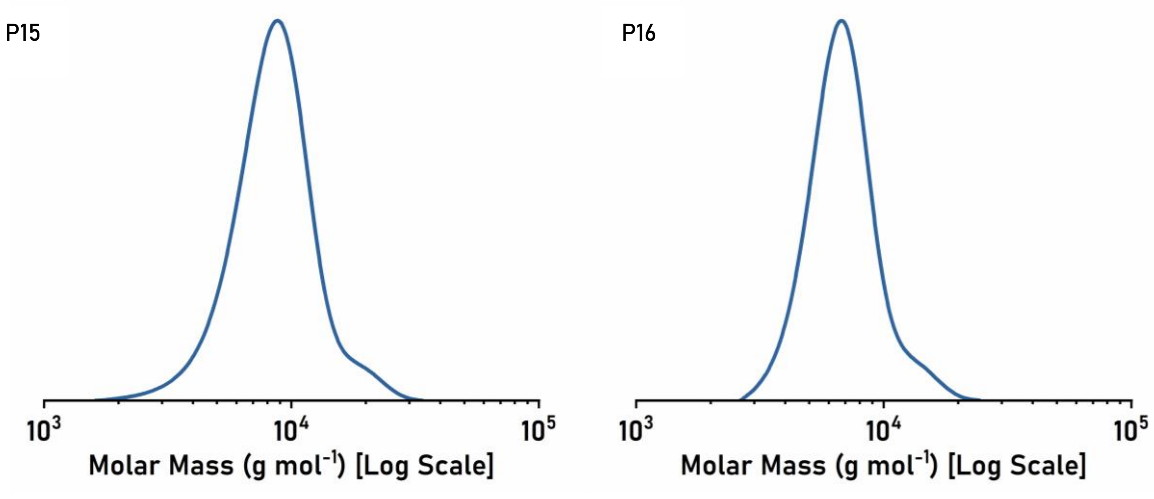

Figure S97: GPC chromatograms for polymers described in Scheme 2, **P15-16**.

5.3 Kinetic Data for Polymers Described in Scheme 2

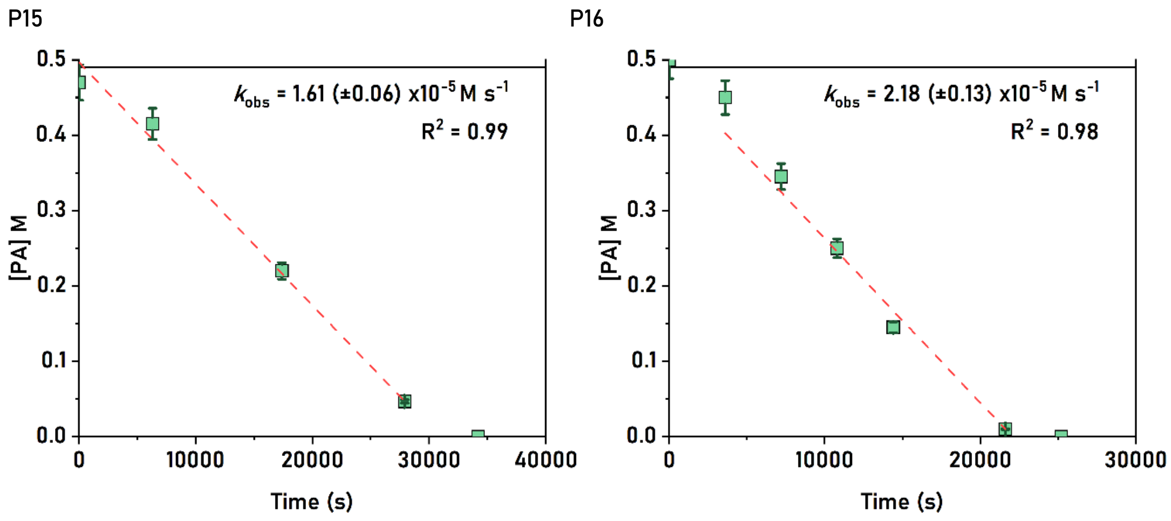

Figure S98: Plots of anhydride concentration vs. time, with linear fits to the data. The order in PA concentration was determined from the gradients of the linear fit. In these experiments [PA] = 0.5 M. This diagram applies to the polymers described in Scheme 2, **P15-16**, read left-to-right.

5.4 DSC Data for Polymers Described in Scheme 2

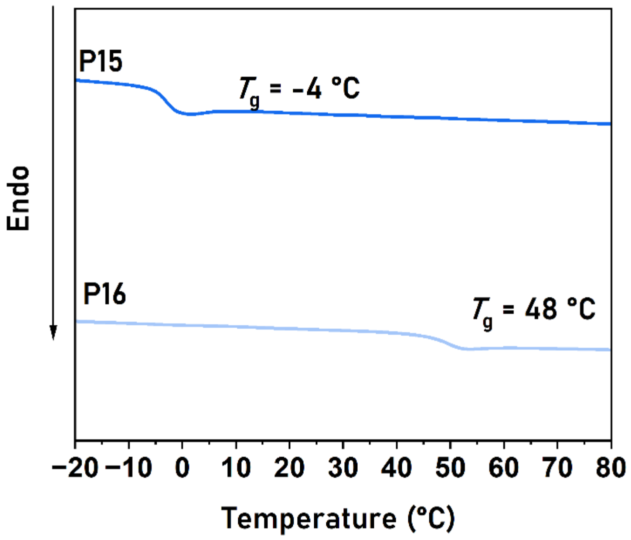

Figure S99: DSC Thermograms for Polymers in Scheme 2, **P15-16**. Data are presented at the glass transition temperatures

5.5 TGA Data for Polymers Described in Scheme 2

Table S6: TGA Data for polymers described in Scheme 2, **P15**-**P16.**

Polymer (#)	Anhyd.	Epoxide	Cyclic Ether	$T_{d,5}$ (°C)	$T_{d,95}$ (°C)
**P15**	PA	BO	DHF	291	383
**P16**	PA	BO	OBH	300	388

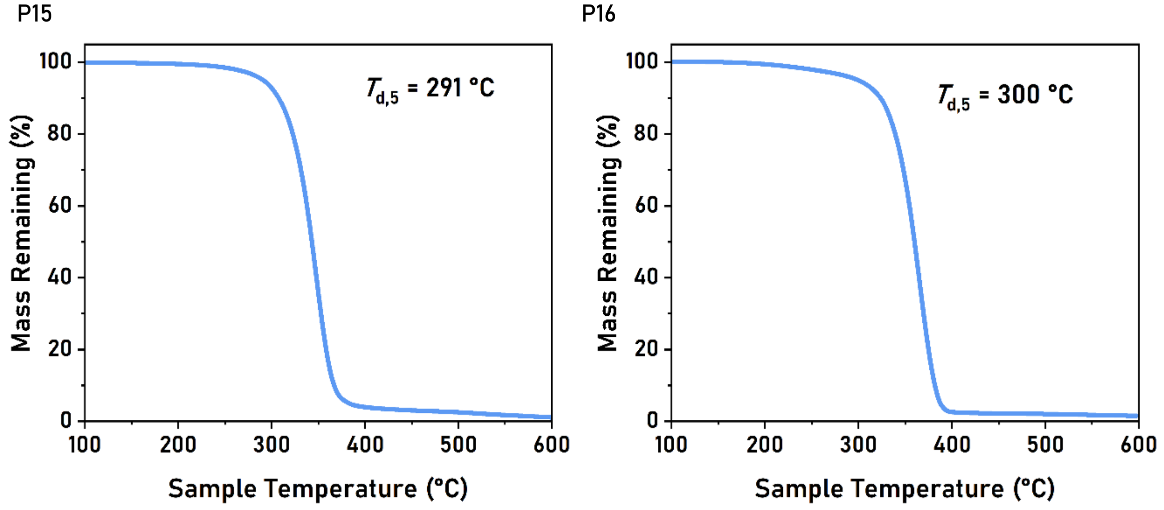

Figure S100: TGA data for polymers described in Scheme 2, **P15-16**

6.0 Characterization Data for Polymers Described in Table 1

6.1 Data for Polymers Described in Scheme 3 – Post-Polymerization Functionalization

6.1.1 Procedure for the Synthesis of (*trans*)-P1

Using a modified general procedure 2.1.1 and literature procedure.[59] In a glovebox, catalyst **1** (9.4 mg, 0.01 mol) was weighed into a vial, then dissolved in butylene oxide (1 mL, 1.2 mmol). Maleic Anhydride (48 mg, 0.5 mmol) was added to the reaction mixture, the vial sealed with electric tape, then parafilm, removed from the glovebox and heated to 50 °C. After 2 hours, diethyl amine (51 µL, 0.5 mmol) was added to the crude reaction mixture and stirred for 3 hours. The crude polymer was characterized as ~ 10 mg/mL THF solution for GPC and ~ 10 mg/mL $CDCl_3$ solution for ^1H NMR spectroscopy. The pure polymer was obtained by precipitation in CH_2Cl_2/MeOH and drying under vacuum at 60 °C, overnight.

6.1.2 General Procedure for 2-Mercaptoethanol Functionalization

Using a modified literature procedure.[60] **P1**, **P10** or **P14** (0.22 mmol relative to the concentration of anhydride in the polymer), 2-mercaptoethanol (2 equivalents relative to the concentration of alkene functional groups in the polymer repeat unit), and DMPA (0.2 equivalents relative to the concentration of alkene functional groups in the polymer repeat unit) was dissolved in THF (2 mL). The reaction was irradiated with UV light and stirred at room temperature for 1 hour. The crude reaction mixture was exposed to air to quench, filtered through a silica plug and precipitated in pentane. The pure polymer was isolated after drying under vacuum at 60 °C, overnight.

Table S7: Comparison of Selected Characterisation Data of **P1**, **P10** and **P14** with **(*trans*)-P1**, **P1s**, **P10s** and **P14s**.

Polymer (#)	Anhyd.	Epoxide	M_n (Đ) [kg mol^{-1}]^a	M_n (Theo)^b	T_g (°C)^c	ΔT_g (°C)^d
P1	MA	BO	8.4 (1.16)	6.6	-19	-1
**(*trans*)-P1**	**FA**	**BO**	**7.9 (1.20)**	**6.6**	**-20**	
P1^e	MA	BO	6.7 (1.22)	6.6	-19	+5
**P1s**	**MA**	**BO**	**6.5 (1.16)**	**8.1**	**-12**	
P10	PA	ED	8.4 (1.21)	12.8	-42	+45
**P10s**	**PA**	**ED**	**10.4(1.37)**	**16.1**	**3**	
P14	MA	ED	10.4 (1.04)	11.2	-50	+65
**P14s**	**MA**	**ED**	**15.5 (1.21)**	**17.5**	**15**	

^{a}Determined by gel permeation chromatography (GPC), using THF as the eluent, and calibrated using narrow MW polystyrene standards (Fig. S90). ^{b}Theoretical M_n are calculated from the original polymer described in Table 1 after full post-functionalisation measured through ^1H NMR). ^{c}Glass transition temperature obtain from Differential Scanning Calorimetry (DSC, second heating cycle, 10 °C min^{-1} heating rate) (Fig. S70 and S91). ^{d}Difference in the glass transition temperature of pre- and post-functionalised polymer. ^{e}A different batch of P1 was used, molar mass and thermal properties are consistent with this sample.

6.2 NMR Spectra for (*trans*)-P1

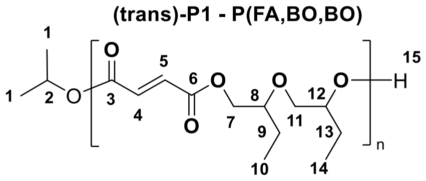

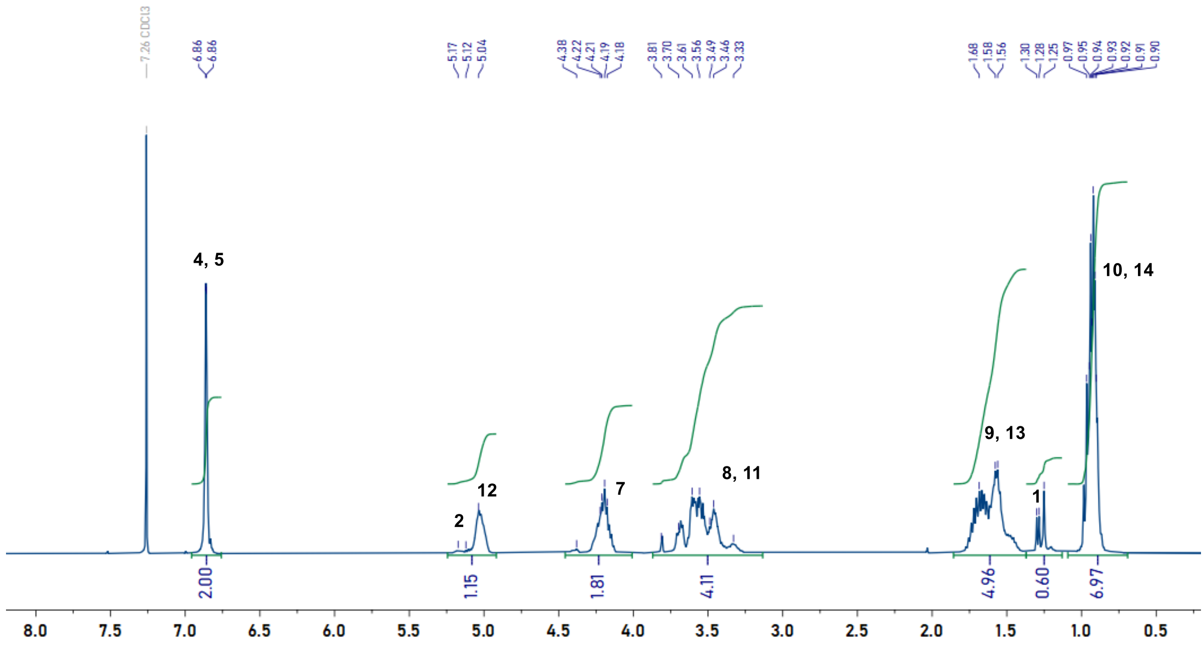

Figure S101: ^{1}H NMR spectrum from the reaction of **P1** with $HNEt_2$, (work-up in MeOH/ CH_2Cl_2). Spectrum corresponds to Scheme 3 and Table S3, (*trans*)-**P1** (400 MHz, $CDCl_3$).

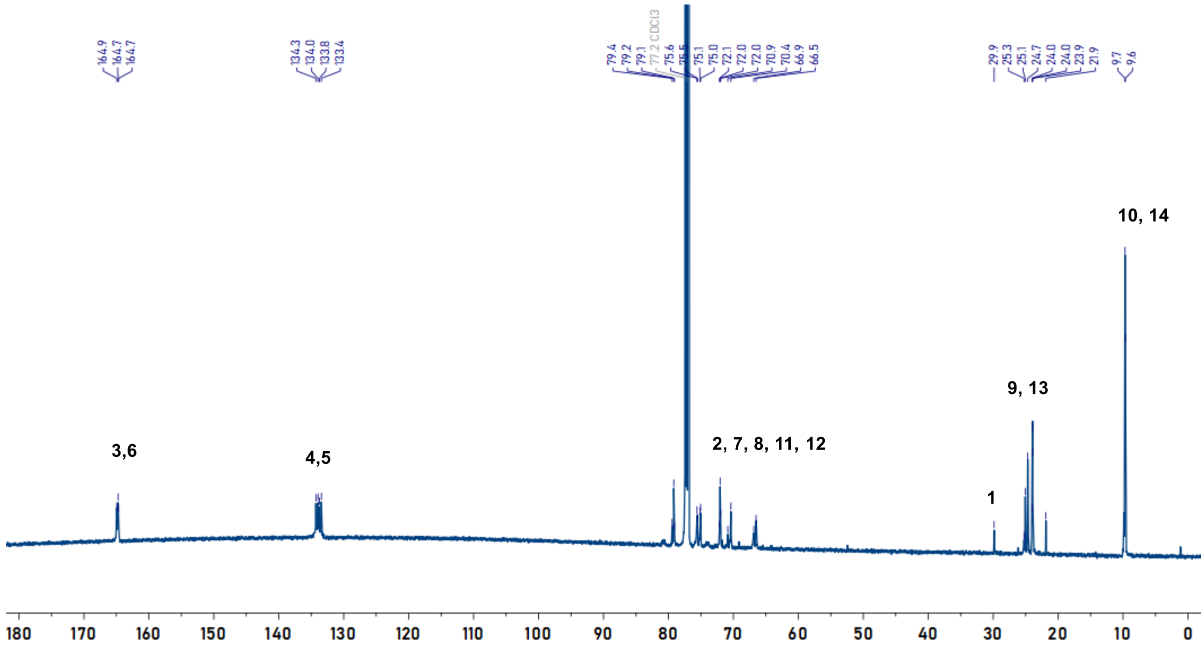

Figure S102: $^{13}C\{^{1}H\}$ NMR spectrum from the reaction of **P1** with $HNEt_2$, Scheme 3 and Table S3, (*trans*)-**P1** (151 MHz, $CDCl_3$).

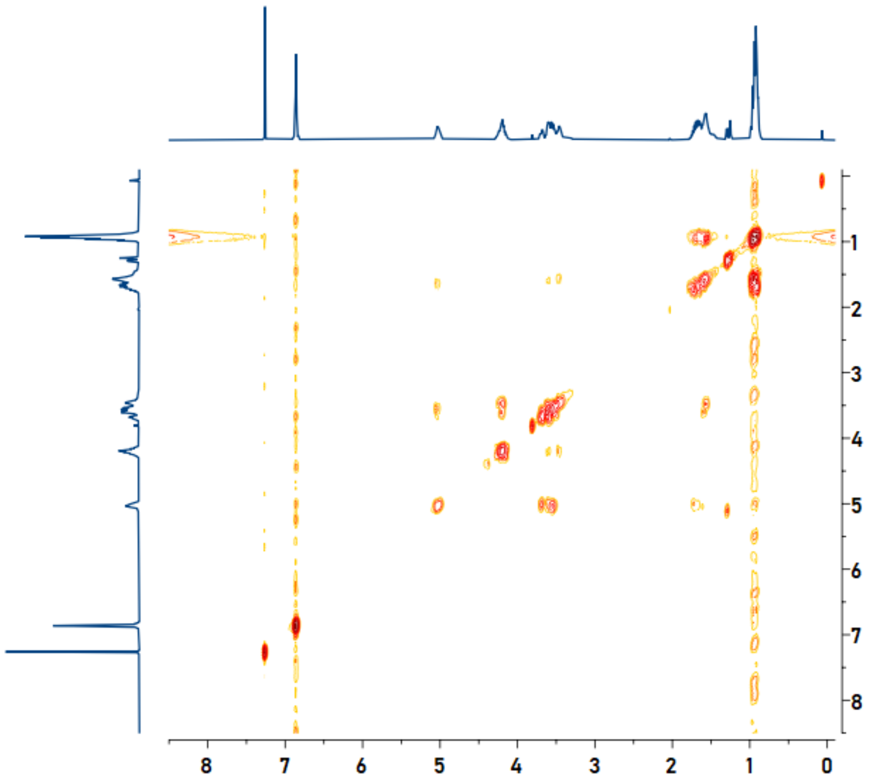

Figure S103: ^{1}H COSY NMR spectrum from the reaction of **P1** with $HNEt_2$, Scheme 3 and Table S3, (*trans*)-**P1** (400 MHz, $CDCl_3$).

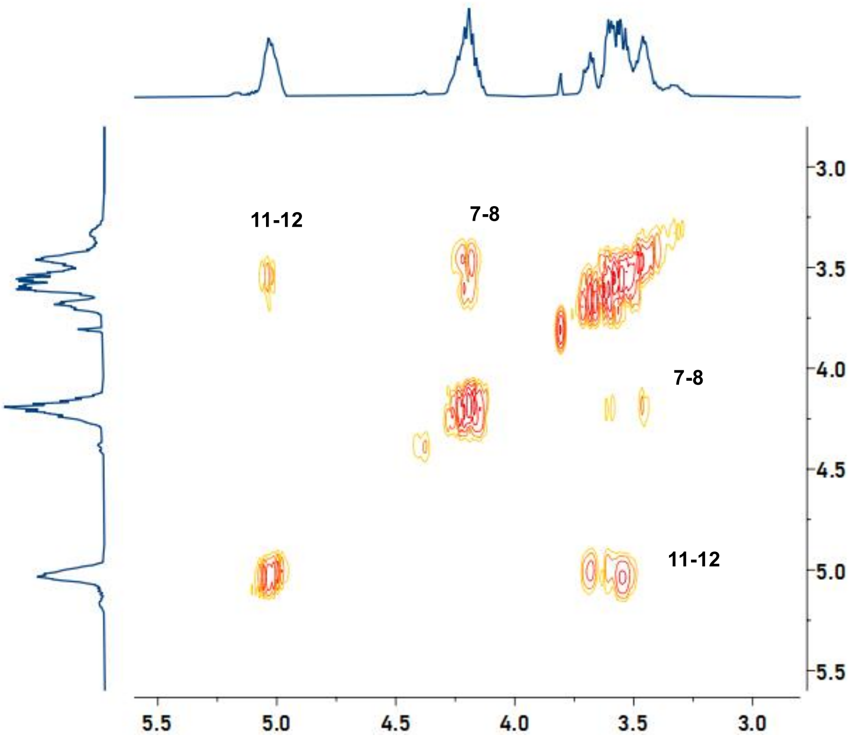

Figure S104: Magnified ^{1}H COSY NMR spectrum from the reaction of **P1** with $HNEt_2$, Scheme 3 and Table S3, (*trans*)-**P1** (400 MHz, $CDCl_3$).

6.3 NMR Spectra for Polymers Described in Table S3 and Scheme 3 – Post-Polymerization Functionalization

6.3.1 NMR Spectra for P1s

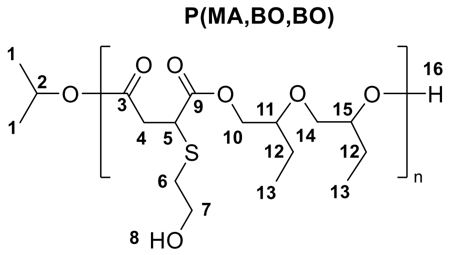

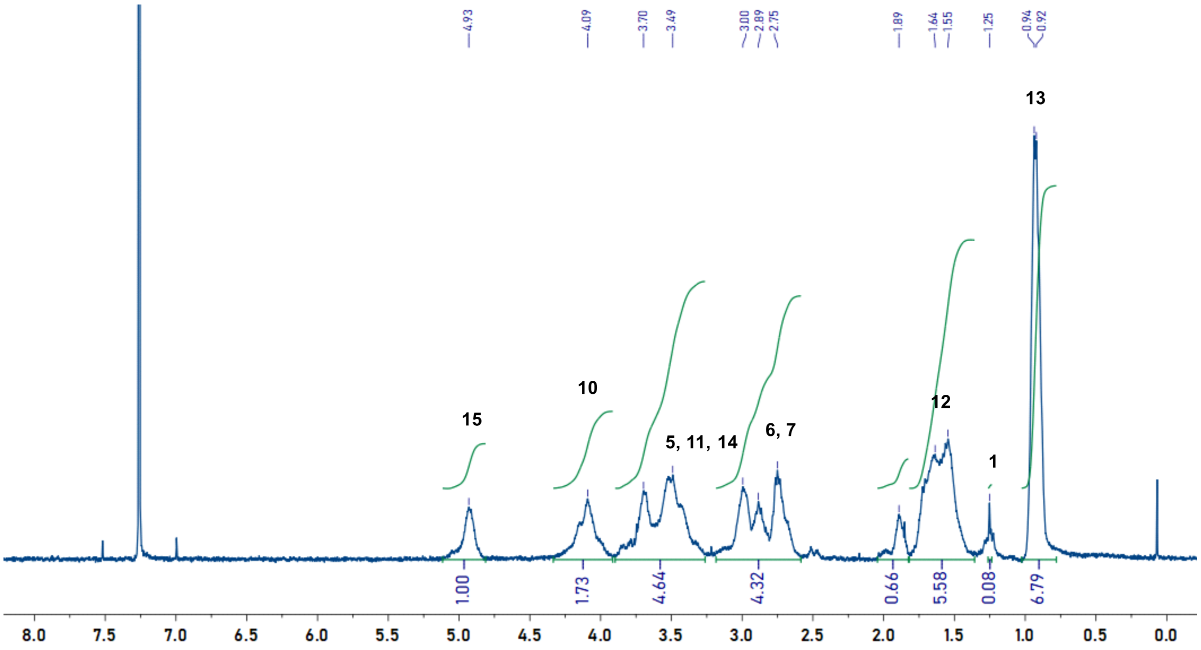

Figure S105: ^{1}H NMR spectrum from the reaction of **P1s** after work-up in pentane. Spectrum corresponds to Scheme 3 and Table S3, **P1s** (400 MHz, $CDCl_3$).

6.3.2 NMR Spectra for P10s

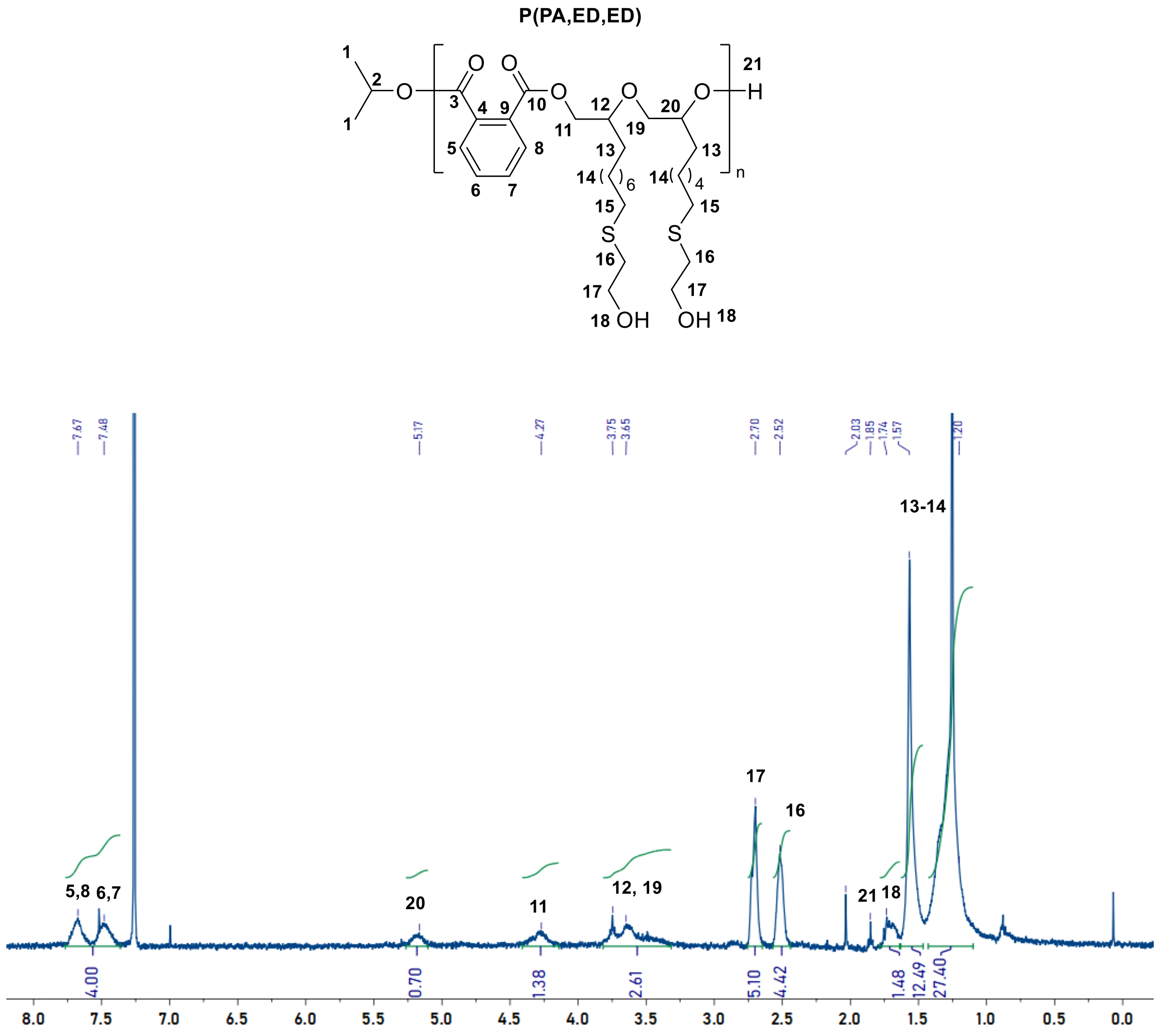

Figure S106: ^{1}H NMR spectrum from the reaction of **P10s** after work-up in pentane. Spectrum corresponds to Scheme 3 and Table S3, **P10s** (400 MHz, $CDCl_3$).

6.3.3 NMR Spectra for P14s

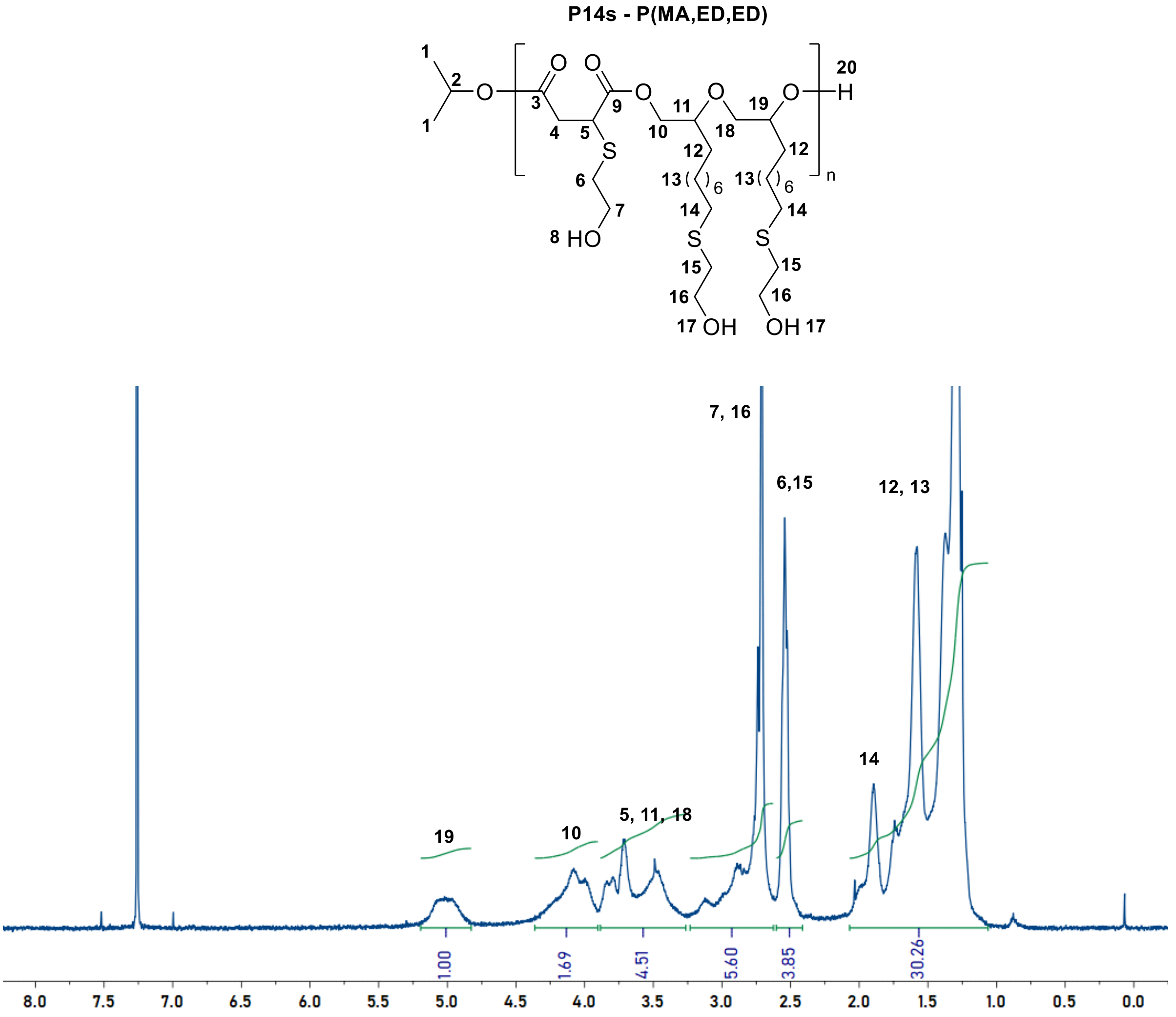

Figure S107: ^{1}H NMR spectrum from the reaction of **P14s** after work-up in pentane. Spectrum corresponds to Scheme 3 and Table S3, **P14s** (400 MHz, $CDCl_3$).

6.4 GPC Data for Polymers Described in Table S3 and Scheme 3 – Post-Polymerization Functionalization

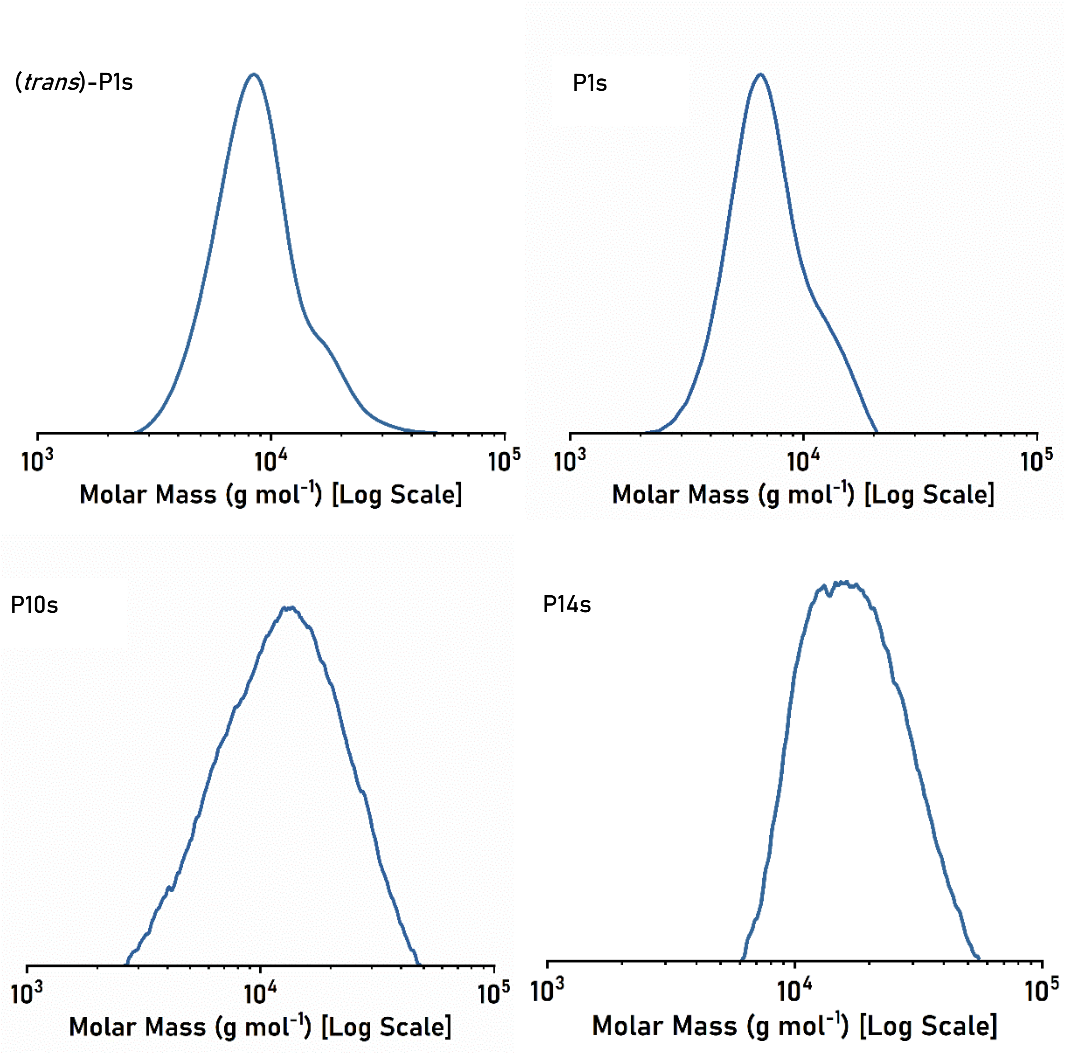

Figure S108: GPC chromatograms for polymers described in Scheme 3, ***(trans)*-P1**, **P1s**, **P10s** and **P14s**.

6.5 DSC Data for Polymers Described in Scheme 3 and Table S3

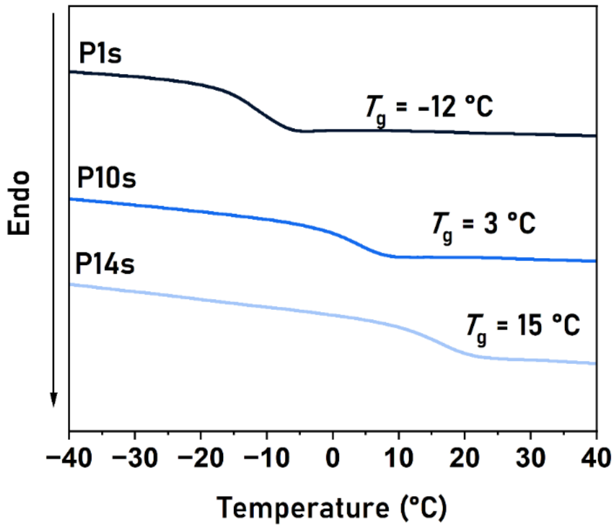

Figure S109: DSC Thermograms for polymers described in Scheme 3, **P1s**, **P10s**, and **P14s**. Data are presented at the glass transition temperatures

Note: DSC data for (*trans*)-**P1** is presented in Figure S70.

6.6 TGA Data of P1s, P10s and P14s .

Table S8: TGA Data for polymers described in Scheme 3, **P1s, P10s** and **P14s**

Polymer (#)	Anhyd.	Epoxide	$T_{d,5}$ (°C)	$T_{d,95}$ (°C)	$T_{d,5}$, *pre-functionalisation* (°C) [#]	$\Delta T_{d,5}$, *pre- and post-functionalisation* (°C)
**P1s**	MA	BO	222	482	306 [**P1**]	84
**P10s**	PA	ED	307	450	308 [**P10**]	1
**P14s**	MA	ED	319	251	344 [**P14**]	25

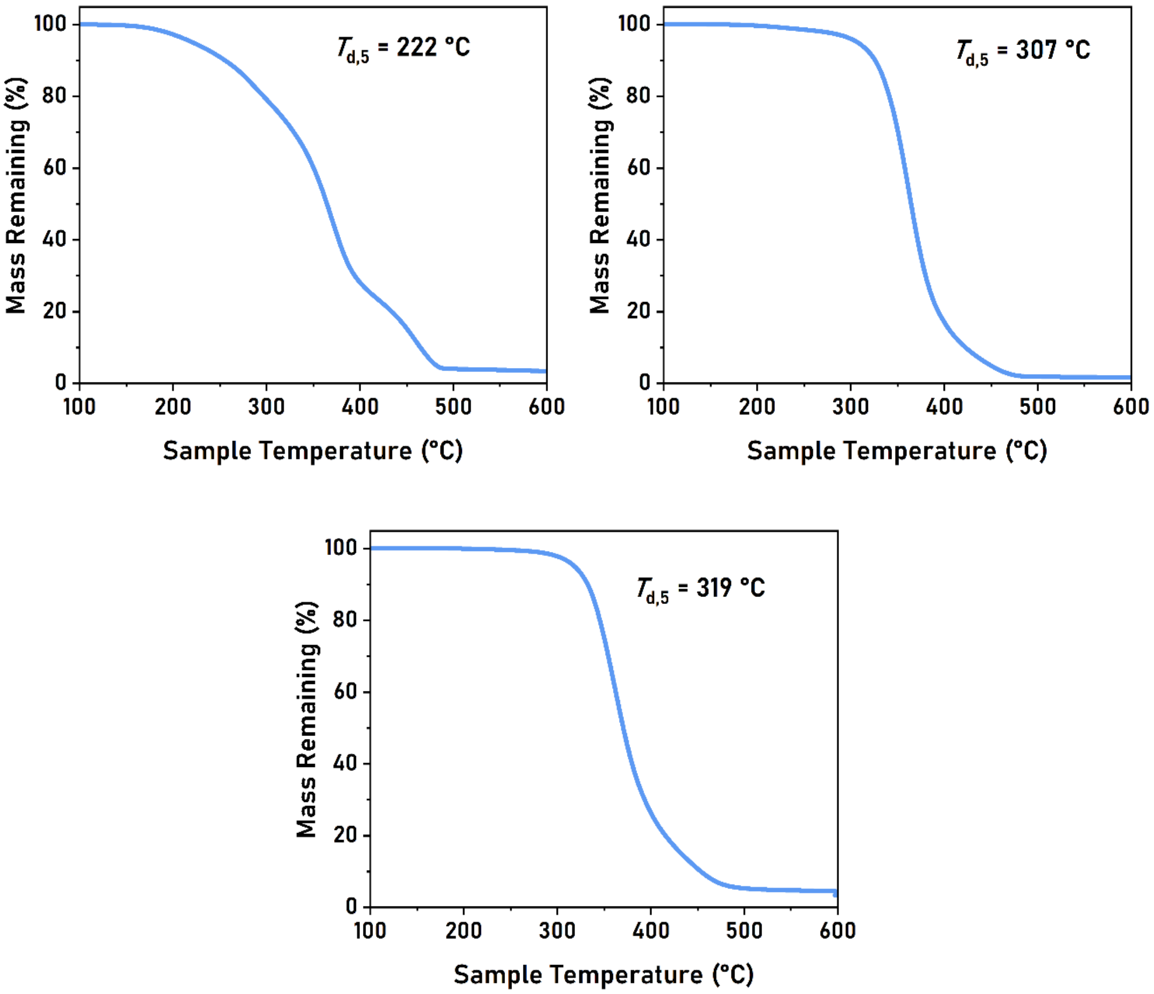

Figure S110: TGA data for polymers described in Scheme 3, **P1s**, **P10s**, and **P14s**.

6.7 Data of P1, P10, P14, P1s, P10s and P14s .

Table S9: Water Contact Angle Data for polymers described in Scheme 3, **P1s, P10s** and **P14s**

Polymer (#)	*Water contact angle* (°)	Polymer (#)	*Water contact angle* (°)	*Δ water contact angle pre- and post-functionalisation* (°)
**P1**	96 ± 2	**P1s**	77 ± 1	19
**P10**	98 ± 4	**P10s**	73 ± 3	25
**P14**	88 ± 3	**P14s**	37 ± 1	51

References

1. R. W. F. Kerr and C. K. Williams, *J. Am. Chem. Soc.*, 2022, **144**, 6882-6893.
2. S. Thiyagarajan, H. C. Genuino, M. Śliwa, J. C. van der Waal, E. de Jong, J. van Haveren, B. M. Weckhuysen, P. C. A. Bruijnincx and D. S. van Es, *ChemSusChem*, 2015, **8**, 3052-3056.
3. X. Shao, L. Su, J. Zhang, Z. Tian, N. Zhang, Y. Wang, H. Wang, X. Cui, X. Hou and T. Deng, *ACS Sustain. Chem. Eng.*, 2021, **9**, 14385-14394.
4. S. Kind, J. Becker and C. Wittmann, *Metab. Eng.*, 2013, **15**, 184-195.
5. A. Wurtz, *Justus Liebigs Ann. Chem.*, 1861, **117**, 136-140.
6. P. McClellan, *Ind. Eng. Chem.*, 1950, **42**, 2402-2407.
7. S.-W. Wan, *Ind. Eng. Chem.*, 1953, **45**, 234-238.
8. A. Wang and T. Zhang, *Acc. Chem. Res.*, 2013, **46**, 1377-1386.
9. J. Zhang, G. A. Lawrance, N. Chau, P. J. Robinson and A. McCluskey, *New J. Chem.*, 2008, **32**, 28-36.
10. P. S. Chowdhury and P. Kumar, *Eur. J. Org. Chem.*, 2013, **2013**, 4586-4593.
11. Y. Nakagawa, T. Kasumi, J. Ogihara, M. Tamura, T. Arai and K. Tomishige, *ACS Omega*, 2020, **5**, 2520-2530.
12. L. Tomaszewska, A. Rywińska and W. Gładkowski, *J. Ind. Microbiol. Biotechnol.*, 2012, **39**, 1333-1343.
13. Y. J. Ng, P. E. Tham, K. S. Khoo, C. K. Cheng, K. W. Chew and P. L. Show, *Bioprocess Biosyst. Eng*, 2021, **44**, 1807-1818.
14. P. Villo, L. Toom, E. Eriste and L. Vares, *Eur. J. Org. Chem.*, 2013, 6886-6899.
15. Y. Suseela and M. Periasamy, *Tetrahedron*, 1992, **48**, 371-376.
16. C. A. Walker, *Ind. Eng. Chem.*, 1949, **41**, 2640-2644.
17. R. S. Atapalkar, P. R. Athawale, D. Srinivasa Reddy and A. A. Kulkarni, *Green Chem.*, 2021, **23**, 2391-2396.
18. Guangxi Tiandong Dasheng Chemical Technology Co Ltd, *CN Pat.,* CN109879712A, 2020.
19. L. Hojabri, X. Kong and S. S. Narine, *Biomacromolecules*, 2010, **11**, 911-918.
20. G. Borsotti, G. Guglielmetti, S. Spera and E. Battistel, *Tetrahedron*, 2001, **57**, 10219-10227.
21. H. G. Alt and M. Jung, *J. Organomet. Chem.*, 1999, **580**, 1-16.
22. G. M. Lari, G. Pastore, C. Mondelli and J. Pérez-Ramírez, *Green Chem.*, 2018, **20**, 148-159.
23. R. J. Ouellette and J. D. Rawn, in *Organic Chemistry Study Guide*, eds. R. J. Ouellette and J. D. Rawn, Elsevier, Boston, 2015, ch. 16, pp. 277-297.
24. X. Li and Y. Zhang, *ACS Catal.*, 2016, **6**, 143-150.
25. H. Deng, R. He, M. Long, Y. Li, Y. Zheng, L. Lin, D. Liang, X. Zhang, M. a. Liao and X. Lv, *Front. Plant Sci.*, 2021, **12**, 728891.
26. S. Pugh, R. McKenna, I. Halloum and D. R. Nielsen, *Metab. Eng. Commun.*, 2015, **2**, 39-45.
27. E. Arceo, J. A. Ellman and R. G. Bergman, *J. Am. Chem. Soc.*, 2010, **132**, 11408-11409.
28. S. Dötterl, U. Füssel, A. Jürgens and G. Aas, *J. Chem. Ecol.*, 2005, **31**, 2993-2998.
29. J. Bomon, M. Bal, T. K. Achar, S. Sergeyev, X. Wu, B. Wambacq, F. Lemière, B. F. Sels and B. U. W. Maes, *Green Chem.*, 2021, **23**, 1995-2009.
30. J. Shi, M. Zhao, Y. Wang, J. Fu, X. Lu and Z. Hou, *Journal of Materials Chemistry A*, 2016, **4**, 5842-5848.
31. E. A. Fehnel, S. Goodyear and J. Berkowitz, *J. Am. Chem. Soc.*, 1951, **73**, 4978-4979.
32. P. Mäki-Arvela, T. Salmi, B. Holmbom, S. Willför and D. Y. Murzin, *Chem. Rev.*, 2011, **111**, 5638-5666.
33. T. A. Gokhale, A. B. Raut and B. M. Bhanage, *Mol. Catal.*, 2021, **510**, 111667.
34. J. G. Stevens, R. A. Bourne, M. V. Twigg and M. Poliakoff, *Angew. Chem. Int. Ed.*, 2010, **49**, 8856-8859.
35. N. Alonso-Fagúndez, M. L. Granados, R. Mariscal and M. Ojeda, *ChemSusChem*, 2012, **5**, 1984-1990.
36. E. Mahmoud, D. A. Watson and R. F. Lobo, *Green Chem.*, 2014, **16**, 167-175.
37. A. Alhanash, E. F. Kozhevnikova and I. V. Kozhevnikov, *Applied Catalysis A: General*, 2010, **378**, 11-18.
38. H. Luo, L. Ge, J. Zhang, J. Ding, R. Chen and Z. Shi, *Bioresour. Technol.*, 2016, **200**, 111-120.
39. B. Cao, J. Zhang, J. Zhao, Z. Wang, P. Yang, H. Zhang, L. Li and Z. Zhu, *ChemCatChem*, 2014, **6**, 1673-1678.
40. Y. Hu, N. Li, G. Li, A. Wang, Y. Cong, X. Wang and T. Zhang, *ChemSusChem*, 2017, **10**, 2880-2885.
41. A. Pan, M. Chojnacka, R. Crowley, L. Göttemann, B. E. Haines and K. G. M. Kou, *Chem. Sci.*, 2022, **13**, 3539-3548.
42. Zhengzhou Gecko Scient Inc., *CN Pat.,* CN108047089B, 2020.
43. M. Hanack, J. Metz and G. Pawlowski, *Chem. Ber.*, 1982, **115**, 2836-2853.
44. T. Maki and K. Takeda, in *Ullmann's Encyclopedia of Industrial Chemistry*, 2000, DOI: https://doi.org/10.1002/14356007.a03_555.

45. K. T. Ziebart and M. D. Toney, *Biochemistry*, 2010, **49**, 2851-2859.
46. S. Samadi, K. Jadidi and B. Notash, *Tetrahedron: Asymmetry*, 2013, **24**, 269-277.
47. W. Han, S. Qin, X. Shu, Q. Wu, B. Xu, R. Li, X. Zheng and H. Chen, *RSC Adv.*, 2016, **6**, 53012-53016.
48. M. Tarasenko, N. Duderin, T. Sharonova, S. Baykov, A. Shetnev and A. V. Smirnov, *Tetrahedron Lett.*, 2017, **58**, 3672-3677.
49. T. Rohand, J. Savary and I. E. Markó, *Monatsh. Chem.*, 2018, **149**, 1429-1436.
50. L. Y. Gur'eva, A. K. Bol'sheborodova and Y. L. Sebyakin, *Russ. J. Org. Chem.*, 2012, **48**, 1047-1054.
51. G. Kantin, E. Chupakhin, D. Dar'in and M. Krasavin, *Tetrahedron Lett.*, 2017, **58**, 3160-3163.
52. H. Niu, J. Luo, C. Li, B. Wang and C. Liang, *Ind. Eng. Chem. Res.*, 2019, **58**, 6298-6308.
53. R. Tesser, E. Santacesaria, M. Di Serio, G. Di Nuzzi and V. Fiandra, *Ind. Eng. Chem. Res.*, 2007, **46**, 6456-6465.
54. Hubei Greenhome Fine Chemical Co LTD, *CN Pat.,* CN104592166B, 2016.
55. Yeo Myung Biochem Co LTD, *KR Pat.,* KR101528751B1, 2015.
56. S. Miyano, L. D. L. Lu, S. M. Viti and K. B. Sharpless, *J. Org. Chem.*, 1985, **50**, 4350-4360.
57. A. Spyros, D. S. Argyropoulos and R. H. Marchessault, *Macromolecules*, 1997, **30**, 327-329.
58. Z.-H. Jiang, D. S. Argyropoulos and A. Granata, *Magn. Reson. Chem.*, 1995, **33**, 375-382.
59. A. M. DiCiccio and G. W. Coates, *J. Am. Chem. Soc.*, 2011, **133**, 10724-10727.
60. K. C. Poon, G. L. Gregory, G. S. Sulley, F. Vidal and C. K. Williams, *Adv. Mater.*, 2023, **35**, 2302825.
